# Branched-chain actin dynamics polarizes vesicle trajectories and partitions apicobasal epithelial membrane domains

**DOI:** 10.1126/sciadv.ade4022

**Published:** 2023-06-28

**Authors:** Gholamali Jafari, Liakot A. Khan, Hongjie Zhang, Edward Membreno, Siyang Yan, Graham Dempsey, Verena Gobel

**Affiliations:** ^1^ Mucosal Immunology and Biology Research Center, Developmental Biology and Genetics Core, MGH*f*C, Harvard Medical School, Boston, MA, USA.; ^2^ Faculty of Health Sciences, University of Macau, Taipa, Macau, China.; ^3^ Chemistry and Chemical Biology Department, Harvard University, Cambridge, MA, USA.

## Abstract

In prevailing epithelial polarity models, membrane- and junction-based polarity cues such as the partitioning-defective PARs specify the positions of apicobasal membrane domains. Recent findings indicate, however, that intracellular vesicular trafficking can determine the position of the apical domain, upstream of membrane-based polarity cues. These findings raise the question of how vesicular trafficking becomes polarized independent of apicobasal target membrane domains. Here, we show that the apical directionality of vesicle trajectories depends on actin dynamics during de novo polarized membrane biogenesis in the *C. elegans* intestine. We find that actin, powered by branched-chain actin modulators, determines the polarized distribution of apical membrane components, PARs, and itself. Using photomodulation, we demonstrate that F-actin travels through the cytoplasm and along the cortex toward the future apical domain. Our findings support an alternative polarity model where actin-directed trafficking asymmetrically inserts the nascent apical domain into the growing epithelial membrane to partition apicobasal membrane domains.

## Teaser

A basolateral-to-apical F-actin shift confers directionality to vesicles that asymmetrically insert the nascent apical membrane.

## INTRODUCTION

The prevailing polarity models place membrane- and junction-based apicobasal core polarity cues (e.g., the partitioning defective PARs) upstream of intracellular cues during the establishment and modulation of plasma membrane polarity in epithelial cells (*1*, *2*). Once these core polarity cues have demarcated the positions and determined the identities of apicobasal membrane domains, these domains are thought to polarize intracellular processes, for instance, by providing cognate recognition sites for the vesicle-based sorting of apicobasal cargo that expands and maintains these domains (*3*). Domain exclusion of membrane-based core polarity cues (e.g., the PARs) is also considered a principal mechanism for modulating membrane polarity in the tissue context (*4*). Downstream of extracellular signals that orient cells within the epithelium, membrane- and junction-based core polarity cues are thus thought to impart the initiating polarizing event at the cell membrane, during both the establishment and modulation of epithelial membrane polarity. In these models, the process of polarized membrane biogenesis is viewed as the addition of molecules to, or the exchange of molecules within, membrane domains previously demarcated by the membrane-based polarity cues.

Recent findings have challenged these prevailing polarity models: (i) membrane-based apicobasal polarity cues (e.g., the PAR, Crumbs, and Scribble polarity complexes) appear to be dispensable for the polarity of some epithelia (*5*); (ii) multiple vesicle-based endo- and transcytotic-recycling components were identified that maintain or alter the position of apical membrane components and polarity cues (e.g., the PARs) in different tissues and position the lumen in tubular epithelia (*6*–*12*). These findings raised the questions: (i) what, if any, are the redundant or alternative modes of membrane polarization, and (ii) how can intracellular processes such as vesicular trafficking determine the position of polarized membrane components in the absence of demarcated target membrane domains?

We previously identified several trafficking molecules from unbiased *Caenorhabditis elegans* tubulogenesis screens whose perturbation reversibly changed the position of the apical membrane domain (lumen) in already polarized intestinal cells, overriding the polarity established by the core polarity cues (apicobasal membrane polarity conversion). The analysis of this polarity phenotype revealed that vesicle membrane and coat components [glycosphingolipids (GSLs), clathrin, and its AP-1 adaptor] can determine the position of the apical domain, upstream of PARs, in still dividing cells of the early-embryonic intestine (clathrin) and in postmitotic but still growing cells of the larval intestine (AP-1, GSLs) (*7*, *10*). Our accompanying article (*13*) demonstrates that multiple early (pre-Golgi) and late (post-Golgi) components of the anterograde (membrane-directed) biosynthetic-secretory (secretory) trafficking pathway are also required to determine the position of the apical domain in the developing *C. elegans* intestine, operating on both, not-yet polarized and already-polarized, expanding membranes. On the basis of these findings, we proposed an alternative mode of polarized membrane biogenesis, where the vesicle-based asymmetric insertion of the nascent apical domain into the growing membrane partitions apical and basolateral membrane domains (*13*). It remained unclear, however, how the secretory pathway that supplies all sides of the membrane sorts apical cargo to a not-yet polarized membrane during polarity establishment and to a basolateral (nontarget) membrane domain in already polarized cells.

Here, we focus on three components of the branched-chain actin machinery that we identified by the same apicobasal polarity conversion phenotype in the same tubulogenesis screens that identified the vesicle-based polarity cues (*7*, *10*, *13*). Our analysis reveals that branched-chain actin dynamics powers a vectorial F-actin network that moves from the basolateral to the future apical domain in polarizing *C. elegans* intestinal cells. All components of the branched-chain actin machinery and actin itself are required to route pre- and post-Golgi vesicles to the nascent apical domain during de novo polarized membrane biogenesis. These findings support the proposed alternative mode of polarized membrane biogenesis in epithelia (*13*) and suggest a mechanism for how anterograde vesicle trajectories can acquire long-range directionality independent of sorting to previously demarcated membrane domains.

## RESULTS

### The branched-chain actin modulators UNC-60, ARX-2, and CAP-1 determine the position of the apical domain (lumen) in the embryonic and larval *C. elegans* intestine

**Fig. 1.**
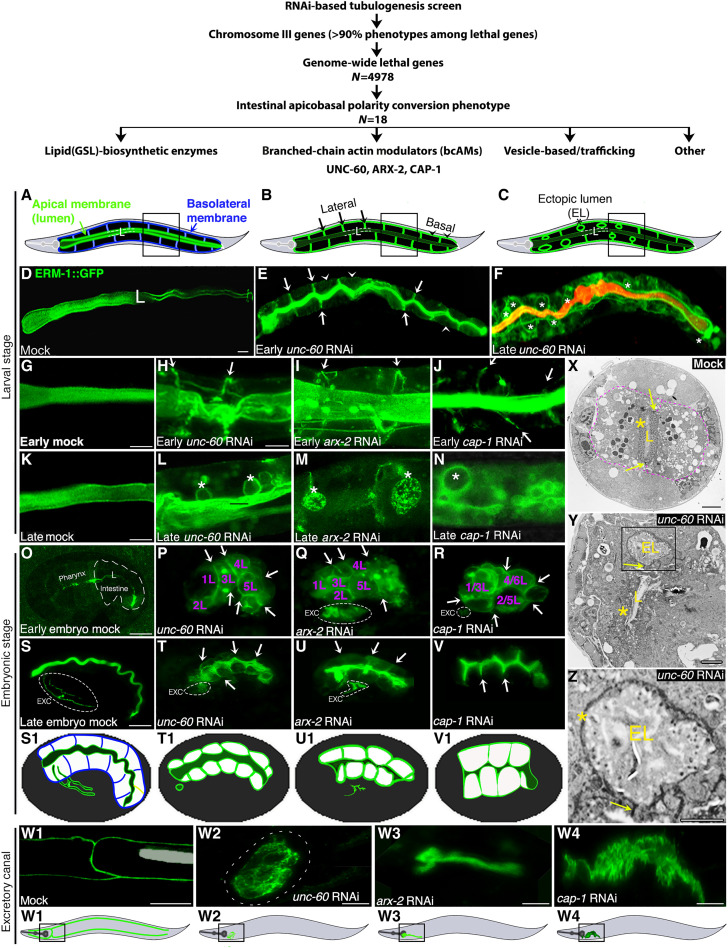
The three bcAMs UNC-60/cofilin, ARX-2/Arp2/3, and CAP-1/actin capping determine the position of the intestinal apical domain/lumen. (**A** to **C**) Schematic of apicobasal polarity conversion (20-cell intestine: black). (A) Wild-type, apical = lumenal membrane: green; BL (basolateral) membrane: blue, (B) early-stage-, (C) late-stage polarity conversion. Basolateral ELs (ectopic lumens; asterisks). Some of the lateral (arrows) and basal (arrowheads) BL membrane domains are indicated. Anterior: left, dorsal: up. (**D**) Wild-type (mock) ERM-1::GFP identifies the apical/lumenal intestinal membrane. (**E**) Basolateral (arrows/arrowheads) and cytoplasmic ERM-1 mislocalization at early-stage and (**F**) ELs (asterisks) at late-stage polarity conversion. Intralumenal DsRed bacteria do not fill the ELs. (**G** to **N**) Magnified view of boxes in (A) to (C). Basolateral ERM-1 displacement (arrows) and ELs (asterisks). (M) Speckled ERM-1::GFP in ELs suggests dysmorphic microvilli. (**O** to **R**) Failure of ERM-1 polarization during polarity establishment in pre-intercalation intestines (arrows: basolateral mislocalization; E/intestinal cell identities: purple). (**S** to **V**) Basolateral ERM-1::GFP post-intercalation (arrows). (O and S): ERM-1 is always apical in wild type. (S1 to V1) Cartoons for (S to V). (**W1 **to **W4**) EXC (excretory canal): (W1) The single-cell EXC extends four arms through the animal. (W2 to W4) No EXC lumen extension (EXC is circled in Q to U and W2). Schematics indicate area shown above (boxed). (**X** to **Z**) Transmission electron microscopic (TEM) cross-sections of larval intestines (two cells are outlined by purple line in (X): lumen (L) with dense microvilli (yellow star) in (X) versus disorganized microvilli in (Y), and ectopic lumen (EL; boxed) in (Y), magnified below. (Z) EL with sparse dysmorphic microvilli (yellow star). Yellow arrows: intact apical junctions. Scaled-intensity RNAi is used throughout. Confocal images are shown in A to W4. Additional defects are described in fig. S1. See fig. S3 for intestinal morphogenesis. Scale bars, 20 μm (D to F), 10 μm (G to N and O to W1), 5 μm (W2 to W4), and 2 μm (X to Z).

In an RNAi-based genome-scale tubulogenesis screen, we identified several genes whose knockdowns induced apicobasal membrane polarity conversion in the developing *C. elegans* intestine [Fig. 1; see (*7*, *10*) and (*13*) for the screen design]. Polarity conversion was defined as the basolateral displacement of apical membrane components in any or all of the 20 cells of this single-layered epithelium, with and without subsequent ectopic basolateral lumen formation (full transformation of the basolateral into a junction-bounded apical membrane with microvilli, a process that evolves over time). Polarity conversion can be visually tracked by the apical membrane identity marker ERM-1/ERM/ezrin-radixin-moesin [for the experimental approach, see the accompanying article (*13*): “An approach used to track de novo polarized membrane biogenesis in single cells in vivo”].

Among the 18 genes isolated in these screens by an intestinal polarity conversion phenotype, we identified all three components of the branched-chain actin machinery [here referred to as branched-chain actin modulators (bcAMs)]: *unc-60*/cofilin (actin depolymerizing), ARX-2/ARP-2/3 (actin nucleating), and CAP-1 (actin capping) (Fig. 1). To investigate these pleiotropic molecules’ function in polarized membrane biogenesis without disrupting their essential cellular functions, we generated a range of mild to moderately severe phenotypes using a scaled-intensity RNAi approach (Materials and Methods). This approach revealed that ERM-1 (i) was displaced to basolateral domains and ectopic lumens in *unc-60*, *arx-2, cap-1(mild-RNAi)* late-embryonic/larval intestinal cells (Fig. 1, D to N) and (ii) failed to be polarized to the apical domain in *unc-60, arx-2, cap-1(RNAi)* early-embryonic intestinal cells (Fig. 1, O to V). Thus, each of these three bcAMs is required to determine the position of the apical domain during de novo polarized membrane biogenesis in the developing *C. elegans* intestine [see fig. S3 for *C. elegans* intestinal morphogenesis (intercalation) and net apical membrane addition during development, glossary of terms for distinct membrane biogenesis defects, and criteria used to distinguish apical membrane biogenesis from positioning (polarity) defects]. bcAM loss also aborted apical membrane (lumen) expansion inside the single-cell excretory canal (Fig. 1, W1 to W4).

Germline deletions and intestine-specific RNAi confirmed the *bcAM(RNAi)* phenotypes, suggested that maternal products contribute to bcAMs’ polarity function, and demonstrated that bcAMs function cell-autonomously in the intestine [hence, it is presumed that the UNC-60 function is mediated by its intestine-specific isoform UNC-60A (*14*); fig. S1]. Confocal analysis of polarized membrane markers and functional studies in larval intestines revealed that mild interference with each of the three bcAMs changed the polarity of multiple apical, but not of basolateral, membrane components while preserving the integrity of apical junctions that secure the positions of apical and basolateral membrane domains [fig. S1; note that excess ectopic lateral junctions form around basolateral lumens at later stages of polarity conversion (Fig. 1, X to Z); see fig. S1 for additional bcAM effects on membrane and junction biogenesis]. Transmission electron microscopy (TEM) confirmed the formation of junction-bounded ectopic basolateral lumens with microvilli in *unc-60(RNAi)* larval intestines with late-stage polarity conversion (Fig. 1, X to Z). All these phenotypic features copy key aspects of apicobasal polarity conversion induced by the loss of each of multiple trafficking molecules previously identified by intestinal polarity conversion in the same tubulogenesis screens (*7*, *10*, *13*).

We conclude that UNC-60, ARX-2, and CAP-1 are required to determine the position of the apical domain and thus membrane polarity in the *C. elegans* intestine. The close similarity of the bcAM- and trafficking-dependent polarity phenotypes (*13*) suggested that bcAMs and trafficking function together in the regulation of epithelial membrane polarity by determining the position of the apical domain on the expanding membrane [see the accompanying article (*13*)].

### Actin itself is required to position the apical domain, and UNC-60, ARX-2, and CAP-1 interact with each other and with actin in this function

**Fig. 2.**
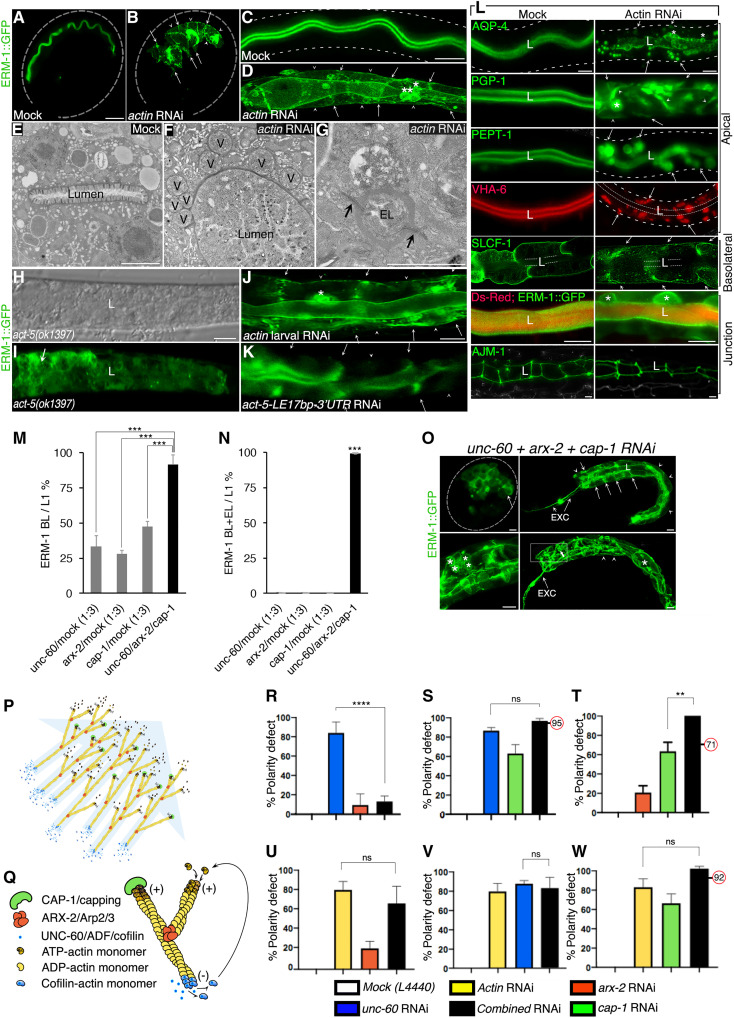
Actin determines the position of the apical domain and interacts with the bcAMs in polarity. (**A**) Apical ERM-1::GFP in wild-type embryonic intestine (dashed line: eggshell). (**B**) “Actin” RNAi [*act-5* clone/Ahringer Library (*114*); fig. S2B] prevents ERM-1 polarization during polarity establishment (arrows: lateral, arrowheads: basal domains of basolateral membranes; not all indicated). (**C** and **D**) Wild-type versus *actin(RNAi)–*induced polarity conversion in L1-larval intestines. (C) Dashed lines: ERM-1–negative basal membrane. (D) Note lumenogenesis defects, cytoplasmic ERM-1, apical vacuoles, ectopic lumens (ELs; asterisks). (**E** to **G**) TEM cross-sections (L1-larval intestine). (E) Lumen with intact microvilli, (F) dilated lumen with loss of microvilli (V: apical vacuoles), (G) magnified basolateral EL with intact junctions (arrows). (**H** and **I**) Cytoplasmic ERM-1::GFP displacement in *act-5(ok1397)* larval intestine. (**J** and **K**) Basolateral ERM-1::GFP mislocalization (arrows and arrowheads) in *actin(larval-RNAi)* and *act-5-LE17bp-* (*3′UTR-RNAi*) intestines. (**L**) *Actin* RNAi changes the polarized positions of apical, not basolateral, membrane components in the presence of intact junctions. Note SLCF-1 broadening (compare fig. S1). L: lumen; dashed line: basal intestinal border. (**M** to **W**) Genetic interactions. (M to O) Single versus triple *unc-60, arx-2, cap-1* RNAi. (M and N) Quantification. (O) Clockwise: early-embryonic, larval early-stage, larval late-stage (magnified left) polarity conversion. EXC: excretory canal. (P and Q) Filament dynamics. (P) Actin treadmilling. Blue arrow: directionality through concerted force of bcAMs. (Q) Capping (CAP-1), branch-nucleating (ARX-2), depolymerizing (UNC-60). (R to T) bcAM interactions (RNAi is titrated; see text). (U to W) The *actin(RNAi)* polarity conversion is not enhanced by bcAMs loss. More than three replicas were analyzed (R to W). Numbers in red circles: additive values. One-way ANOVA for significance: **P < 0.01. Scaled-intensity RNAi is used throughout. Confocal images are shown (H: Nomarski). Scale bars, 10 μm, except (C and D) 50 μm, (E to G) 2 μm, and (J and K) 20 μm.

Currently, actin is considered dispensable for *C. elegans* intestinal polarity (*15*, *16*) and ACT-5 is thought to be the sole intestinal actin (*17*) among the five almost identical *C. elegans* actins (encoded by the *act-1, act-2*, *act-3*, *act-4*, and *act-5* genes; designated actin isoforms below). Since actin is the expected downstream effector of bcAMs, we revisited the question of actin’s function in intestinal polarity. Actin’s presumed dispensability for polarity rests on: (i) *act-5(dt2017/dt2019*) mutant alleles (lethal homozygous progeny of heterozygote parents contain maternal *a*ct-5** product) and (ii) *act-5 * 3′UTR RNAi (mild depletion), which retain the ability to polarize intestinal junctions and cause L1-larval rather than embryonic lethality (*17*); and on (iii) chemical inhibition of actin polymerization that allows PAR-3 to assemble at the intestinal midline (future apical domain) (*15*). Here, we find that the double-stranded RNA (dsRNA) commonly used with the intent to target *act-5* mRNA (fig. S2, B and C) causes embryonic lethality and intestinal polarity defects (Fig. 2, A to G and L; note that this dsRNA targets all five actin isoforms—we thus refer to *act-5* RNAi below as “*actin*” RNAi).

Scaled-intensity *actin* RNAi confirmed the previously reported intestinal structural apical domain biogenesis defects caused by loss of ACT-5 (complete loss of microvilli) (*17*) and revealed that mild interference with actin copied the bcAM-dependent apicobasal polarity conversion with failure of ERM-1 polarization in intercalating embryonic intestinal cells (Fig. 2, A and B), basolateral mislocalization of ERM-1 and other apical membrane components (but no apical mislocalization of the basolateral membrane components) in expanding larval cells in the presence of intact junctions (Fig. 2, C, D, and L), and formation of junction-bounded ectopic basolateral lumens at later stages of polarity conversion (Fig. 2, C to G; also note apical membrane blebs and apical vacuoles; see fig. S3 for glossary of terms for distinct membrane biogenesis defects). Conditional *actin* RNAi, induced in L1 larvae, was sufficient to change the polarity of already polarized yet still expanding membranes (Fig. 2J), demonstrating that actin directly affects polarized membrane biogenesis. Thus, actin is required to position the apical domain throughout de novo polarized membrane biogenesis, on not yet polarized membranes of dividing and moving cells during polarity establishment, and on polarized but still expanding membranes of postmitotic cells that have fixed positions within the mature but growing tissue [see the accompanying article (*13*) for the larval *C. elegans* intestine as postmitotic polarity model].

In support of a specific requirement for ACT-5 for polarity, RNAi that predominantly, although not exclusively, targets the ACT-5 3′UTR (designated *act-5-LE17bp *3′UTR RNAi*; LE17bp* = 17 base pairs of the last exonic sequence upstream of the 3′UTR; fig. S2B) copied the *actin(RNAi)* polarity phenotype (Fig. 2K). *act-5(ok1397)*, a large germline deletion (including 500-bp promoter) in a balanced background (i.e., in the presence of maternal *act-5* product; fig. S2D) fully displaced ERM-1 from the membrane, masking possible effects on ERM-1’s positioning at the membrane (Fig. 2, H and I). *act-5-LE17bp *3′UTR RNAi and *act-5(ok1397)*, in contrast to *actin* RNAi, induced larval, not embryonic, polarity defects and lethality. We concluded that actin functions in *C. elegans* intestinal polarity and that ACT-5 requires maternal product and/or additional actin isoforms for its polarity function.

To determine if actin operated as a downstream effector of bcAMs in polarity and if the three bcAMs affected polarity via a joined function in branched-chain actin dynamics, we carried out genetic interaction experiments, using mild RNAi conditions to capture convergent effects on polarity and avoid disrupting essential cellular functions. Triple RNAi, combining all three bcAMs, enhanced the individual *unc-60, arx-2, cap-1(RNAi)* polarity conversion (Fig. 2: additive effect in M, enhancement in N and O), supporting the joined function of the three bcAMs in polarized membrane biogenesis (note that only mild RNAi is used in all genetic interaction experiments, here and below, and that only early polarity conversion on expanding L1-larval membranes is assessed; stronger depletion of *unc-60, arx-2, cap-1,* and *actin* causes a highly penetrant polarity phenotype, compounded by sterility and early lethality; fig. S2A).

Branched-chain actin operates (i) by cofilin(UNC-60)–mediated filament disassembly, (ii) by Arp2/3(ARX-2)–mediated branched filament assembly, and (iii) with capping (CAP-1) promoting both cofilin-dependent depolymerization at the pointed filament end (direct interaction) and Arp2/3-dependent branching by limiting filament elongation at the barbed end (indirect interaction; Fig. 2, P and Q) (*18*, *19*). Double RNAi (*unc-60/arx-2, unc-60/cap-1, arx-2/cap-1*; Fig. 2, R to T) reflected the three bcAMs’ distinct functions in filament modulation and revealed a requirement for stoichiometry, consistent with a joint function of bcAMs in polarity via branched-chain actin dynamics (treadmilling), known to require stoichiometric contributions of each bcAM (*20*): (i) *unc-60*–dependent polarity conversion was suppressed by RNAi with *arx-2* (Fig. 2R), consistent with the decrease of excess F-actin (assembled due to a loss in *unc-60*–dependent depolymerization) by reducing branched-chain actin nucleation (*arx-2* loss); (ii) *unc-60*–dependent polarity conversion was nonsignificantly enhanced by *cap-1* RNAi (Fig. 2S), consistent with a further increase in F-actin excess by a decline in depolymerization (cooperation of *unc-60* and *cap-1*); (iii) *cap-1–*dependent polarity conversion was enhanced by *arx-2* RNAi (Fig. 2T), consistent with a decrease in branched-chain actin nucleation (loss of *arx-2*) by filament elongation at the barbed end in the absence of capping (*cap-1*). Finally, interference with each bcAM failed to significantly enhance the *actin(RNAi)*-induced polarity conversion (Fig. 2, U to W), in agreement with actin operating as the downstream effector of bcAMs in polarized membrane biogenesis (note that bcAMs’/actin’s genetic interactions in membrane biogenesis are separable from their genetic interactions in lethality; fig. S2A).

We conclude that (i) actin determines the position of the apical domain (lumen), and hence polarity, on expanding membranes of embryonic and larval *C. elegans* intestinal cells; (ii) actin loss phenocopies the bcAM- and trafficking-dependent apicobasal polarity defect; (iii) genetic interactions in polarized membrane biogenesis between different bcAMs and between bcAMs and actin are consistent with these molecules’ combined function in polarity via branched-chain actin filament dynamics. Actin thus appeared as a promising candidate cytoskeletal component to route anterograde vesicle trajectories to the nascent apical membrane domain in polarizing *C. elegans* intestinal cells (see Introduction and our accompanying article) (*13*).

### bcAMs/actins and vesicle-based polarity cues determine the polarized distribution of apical PAR complex components on polarized and on not-yet polarized membranes

Actin and bcAMs have conserved structural functions in cell cortex modeling that include the apical domain and junctions of the *C. elegans* intestine (*21*–*23*). These canonical functions of branched-chain actin dynamics take place in an epithelium already polarized by the membrane-based core polarity cues, e.g., by PAR-3, PAR-6, and PKC-3 (apical PARs), whose polarization is therefore thought to predate these activities (*21*). In contrast, the here-identified function of actin and bcAMs in apical domain positioning are already active at the time of intestinal polarity establishment (Fig. 1, O to R) and operational in the presence of intact junctions (Figs. 1, X to Z, and 2, E to G and L, and fig. S1). These features (early and junction-independent activity) suggested that branched-chain actin dynamics’ function in apical domain positioning was distinct from and anteceded its canonical function in apical domain modeling. Moreover, the same features apply to the previously identified vesicle-based polarity cues’ function in apical domain positioning (*13*), suggesting that branched-chain actin dynamics—like vesicular trafficking—might act at an early step during membrane polarization, upstream of, or concomitant with, membrane-based core polarity cues.

**Fig. 3.**
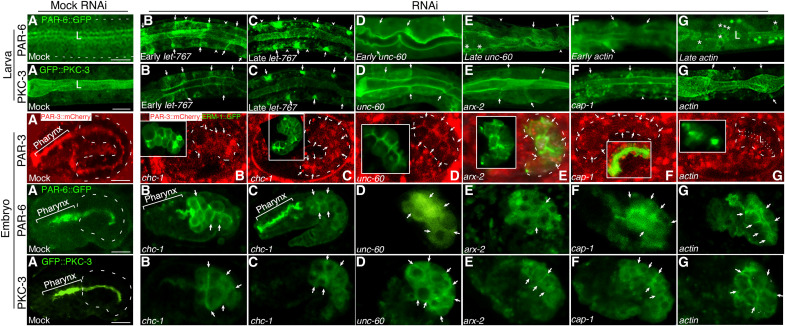
Branched-chain actin dynamics and vesicle-based polarity cues determine the polarized positions of PAR-3, PAR-6, and PKC-3 throughout de novo polarized membrane biogenesis. The upper two rows show portions of early larval intestines, and the lower three rows show embryonic intestines. *unc-60, arx-2, cap-1, and actin*: components of branched-chain actin dynamics. *let-767, chc-1*: trafficking-associated polarity cues. **First and second row, larva:** (**A**) PAR-6::GFP and GFP::PKC-3 at expanding apical membranes in wild-type L1-larval intestines (dashed line: GFP-negative basal membrane, L: lumen). (**B** to **G**) Basolateral and cytoplasmic PAR-6 and PKC-3 mislocalization during early and late polarity conversion. Here and below, arrows indicate some of the lateral, arrowheads some of the basal membrane domains, asterisks some of the basolateral ectopic lumens. **Third to fifth row, embryo:** (**A**) PAR-3, PAR-6, and PKC-3 at apical membranes and junctions in wild-type (mock) embryonic post-intercalation intestines. Note that apical PARs are directly recruited to apical/apico-lateral membranes during wild-type intestinal polarity establishment. (**B** to **G**) Failure of PAR-3, PAR-6, and PKC-3 polarization (cytoplasmic and pan-membranous localization) during polarity establishment in pre-intercalation and intercalating intestines: PAR-3 (B, D, E, and G), PAR-6 (B and D to F), PKC-3 (B, D, and G). Cytoplasmic and basolateral mislocalization (arrows) in post-intercalation intestines [PAR-3 (C and F), PAR-6 (C and G), and PKC-3 (C, E, and F)]. PAR-3::mCherry embryos are double-labeled with ERM-1::GFP that is also mislocalized to the cytoplasm and basolateral membranes [insets; sizes adjusted for clarity; (G) dotted line/L: lumen]. Dashed lines outline intestines. Scaled-intensity RNAi is used throughout. Confocal sections or projections of larvae (2 to 3 pairs of intestinal cells) or embryos (full intestines) are shown. All fluorophore fusions are expressed from their germline loci (table S2). Scale bars, 10 μm. See fig. S3 for schematics of embryonic and larval intestines, intestinal intercalation, and net apical membrane expansion during intestinal development.

To explore this possibility, we examined bcAMs’/actin’s relationship to the apical PARs during de novo polarized membrane biogenesis in the developing intestine and tracked the polarized distribution of endogenously tagged PAR-3, PAR-6, and PKC-3, with and without ERM-1::GFP (green fluorescent protein), throughout net polarized membrane addition in embryonic and larval intestinal cells depleted of bcAMs or actin (Fig. 3; see fig. S3 for intestinal development and net apical membrane addition in embryonic and larval intestinal cells).

Mild RNAi with *unc-60*, *arx-2*, *cap-1*, and *actin* mislocalized apical PAR-6 and PKC-3 to the cytoplasm and to basolateral membrane domains of postmitotic but still expanding larval intestinal cells and, at later stages of polarity conversion, to basolateral ectopic lumens (Fig. 3, first to second row). Stronger RNAi conditions with each of these three bcAMs and with actin prevented the polarization of PAR-3, PAR-6, and PKC-3 in still dividing and intercalating early-embryonic cells, at the time when the intestine’s definitive apicobasal polarity and lumen position are established (Fig. 3, third to fifth row; the expression of PAR-3, considered the earliest acting polarity cue in most epithelia, including the *C. elegans* intestine, weans at the larval stage) (*24*–*26*). PAR-3, PAR-6, and PKC-3 were retained in the cytoplasm of these early-embryonic cells and/or were located at all sides of a membrane that failed to be partitioned into an apical and basolateral domain. *unc-60*, *arx-2*, *cap-1*, and *actin* RNAi also perturbed intestinal intercalation and lumenogenesis, processes that depend on a polarized apical membrane [Fig. 3; (*26*)]. bcAMs’ and actin’s effect on PARs’ polarized distribution at expanding membranes of both larval and embryonic cells copied that of the previously identified vesicle-based apical polarity cues, here shown in *chc-1(RNAi)* early-embryonic, and *let-767(RNAi)* larval, intestinal cells (Fig. 3; *chc-1* encodes the clathrin heavy chain; *let-767* encodes 3-ketocacyl-CoA reductase, a GSL biosynthetic enzyme) (*7*, *10*, *13*).

We conclude that branched-chain actin dynamics, like vesicle-based polarity cues, is required for the polarized distribution of apical PAR complex components on expanding membranes of *C. elegans* intestinal cells, including PAR-3, whose initial recruitment to, and polarized position at, the membrane depends on bcAMs and actin at the time of polarity establishment. Branched-chain actin dynamics, like intracellular trafficking, thus determines the position of the apical domain at an early step during membrane polarization and regulates apicobasal polarity on not-yet polarized and on already polarized but still expanding membranes (see the accompanying article) (*13*).

### UNC-60, ARX-2, CAP-1, and F-actin concurrently shift from the basolateral to the nascent apical domain during intestinal polarity establishment

bcAMs have been associated with anterograde vesicle trajectories in nonpolarized cells (*27*), consistent with the hypothesis that they confer directionality to anterograde trafficking in polarized cells. However, the subcellular localization of bcAMs/ACT-5 at the apical domain of the *C. elegans* intestine (*17*, *21*, *22*) seemed an unlikely position from which to guide vesicles through the cytoplasm. We expressed UNC-60::GFP, ARX-2::GFP, and CAP-1::GFP from the intestine-specific promoter *elt-2* to determine the subcellular localization of these ubiquitously expressed molecules throughout intestinal development.

**Fig. 4.**
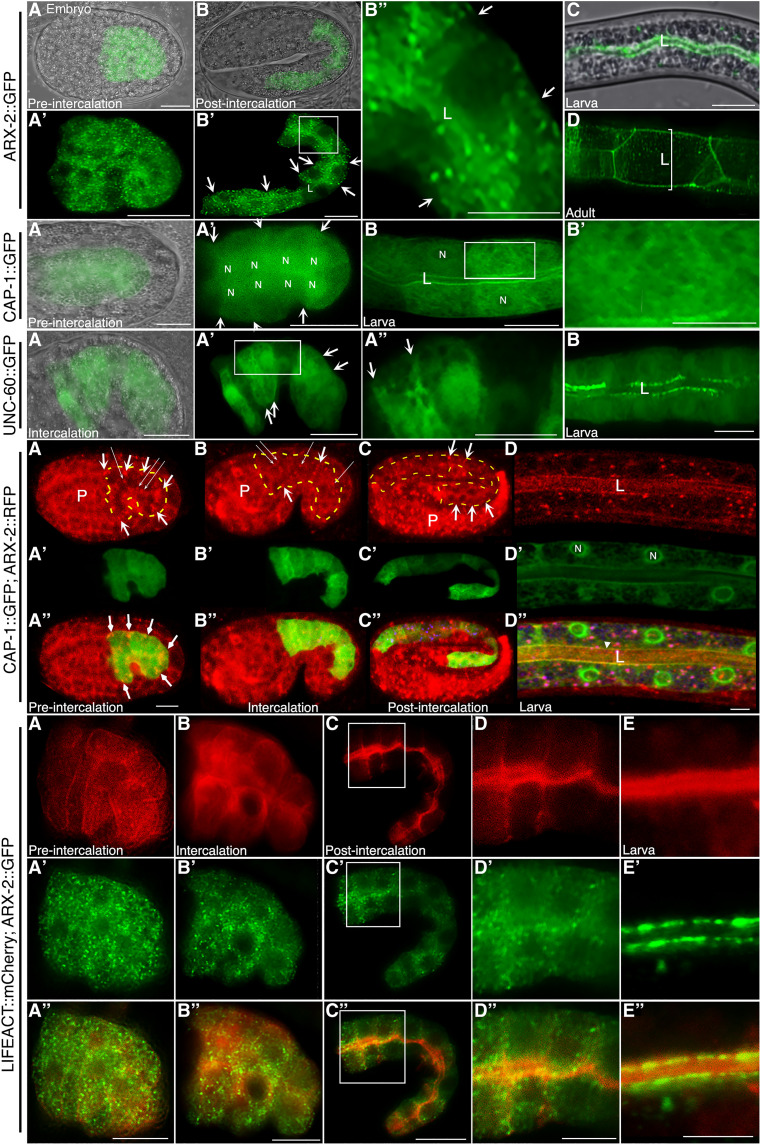
A basolateral-to-apical UNC-60, ARX-2, CAP-1, and F-actin shift during intestinal polarity establishment (developmental subcellular localization profile). **ARX-2::GFP localization:** (**A** and **A′**) Pre-intercalation embryonic intestine: in cytoplasmic and pan-membranous speckles. (**B** and **B′**) Post-intercalation: at apical/lumenal and basolateral membranes (some lateral domains indicated by arrows, here and below). (**B″**) Magnified from (B′). (**C**) Larval intestine: enrichment at the apical membrane (lumen). (**D**) Adult intestine: at the lumen and the peri-lumenal junction belt [magnified belt (bracketed) between two pairs of cells is shown]. L: lumen. **CAP-1::GFP localization:** (**A** and **A′**) Pre-intercalation intestine: cytoplasmic, pan-membranous, peri-nuclear (N: nucleus). (**B**) Larval intestine: enrichment at apical/lumenal membranes (definitive location). (**B′**) Cytoplasmic mesh; magnified from (B). **UNC-60::GFP localization:** (**A** and **A′**) Intercalating embryonic intestine: cytoplasmic, pan-membranous, perinuclear. (**A″**) Cytoplasmic mesh; magnified from (A′). (**B**) Larval intestine: enrichment at apical/lumenal membranes (definitive location). **CAP-1::GFP; ARX-2::RFP colocalization:** (**A** to **C″**) Colocalization: cytoplasmic and pan-membranous in embryonic intestines. (**D** to **D"**) Colocalization: at the apical membrane (L: lumen, arrowhead) but not around nuclei in larval intestine (N: nucleus). Thick arrows: basolateral membranes; thin long arrows: ARX-2::RFP speckles; dashed line: outline of intestine. Image brightness, and hence background, increased for the endogenously expressed ARX-2::RFP (*115*). **LifeACT::mCherry; ARX-2::GFP colocalization:** (**A** to **E″**) F-actin reiterates the three bcAMs’ dynamic developmental subcellular localization pattern during intestinal polarity establishment, shifting from the cytoplasm and all sides of the membrane in the pre-intercalation intestine to the nascent apical domain in the post-intercalation intestine. ARX-2::GFP speckles overlap with LifeACT::mCherry throughout [merged images in (A″) to (E″)]. (A) Pre-intercalation, (B) intercalating, (C) post-intercalation embryonic intestine; boxed areas in (C) to (C″) are magnified in (D) to (D″). (E) to (E″): Lumen between two pairs of larval cells. Confocal sections and Nomarski/confocal overlay images are shown. Scale bars, all panels 10 μm, except (B′, B″, A″, and E″): 5 μm.

In cells of the early-embryonic, pre-intercalation intestine, the three bcAMs were localized at all sides of the membrane (the future apico-basolateral membrane domains) and in the cytoplasm, UNC-60 and CAP-1 in a mesh-like, ARX-2 in a speckled, pattern (Fig. 4, ARX-2::GFP, CAP-1::GFP, and UNC-60::GFP panels: A to A"). During membrane polarity establishment, the three bcAMs shifted from the cytoplasm and the basolateral membrane to their definitive positions at the apical membrane, with ARX-2 additionally labeling apical junctions in the adult intestine (Fig. 4, ARX-2::GFP, CAP-1::GFP, and UNC-60::GFP panels: B to D). Endogenously expressed ARX-2::RFP (red fluorescent protein) overlapped with transgenic CAP-1::GFP during this basolateral-to-apical polarity shift (Fig. 4, CAP-1::GFP; ARX-2::RFP panels).

Next, we labeled intestinal cells with *elt-2p*–directed fluorescent LifeACT, an inert exogenous F-actin–binding molecule, to (i) optimize live imaging of filamentous actin (F-actin) throughout intestinal development, (ii) include all actin isoforms in the analysis, and (iii) avoid possible interference with filament assembly by actin fusion proteins or by endogenous actin-binding molecules. LifeACT reiterated the bcAMs’ dynamic developmental subcellular intestinal expression pattern, also shifting from a cytoplasmic and pan-membranous location to the nascent apical domain during polarity establishment. Double labeling of LifeACT::mCherry and ARX-2::GFP highlighted the synchronous basolateral-to-apical shift of F-actin and ARX-2 (Fig. 4, LifeACT::mCherry; ARX-2::GFP panels: A to E″).

We conclude that the developmental subcellular expression pattern of bcAMs and actin is compatible with a function of branched-chain actin dynamics in guiding vesicles through the cytoplasm to position the nascent apical domain during de novo polarized membrane biogenesis (see our accompanying article) (*13*). In such a function, branched-chain actin dynamics could operate from its transient location in the cytoplasm and at all sides of the membrane (future apico-basolateral membrane domains) of not yet polarized cells, rather than from its definitive location at the apical membrane domain.

### The three bcAMs and actin interact with vesicle-based apical polarity cues in apical domain positioning

**Fig. 5.**
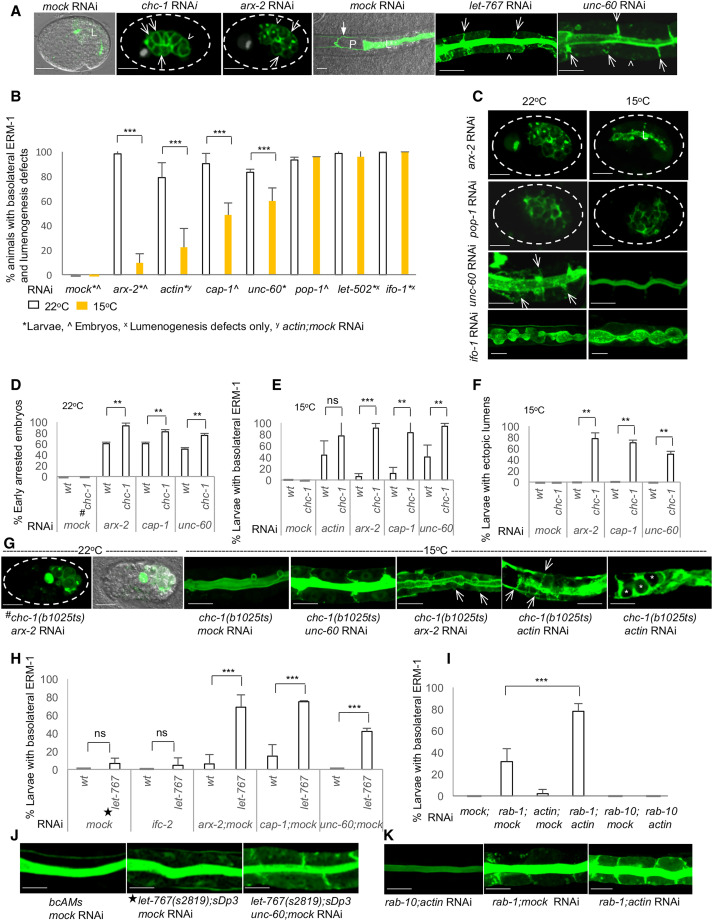
UNC-60, ARX-2, CAP-1, and actin genetically interact with vesicle-based polarity cues in membrane polarity. (**A**) Interference with bcAMs (*arx-2, unc-60*) and vesicle-based polarity cues (*chc-1, let-767*) causes the same polarity conversion phenotype. Arrows indicate: some of the lateral, arrowheads: some of the basal intestinal membrane domains (ERM-1::GFP is shown throughout). Closed arrow in wild-type (mock): excretory canal (grayed out for clarity elsewhere). Dashed lines outline eggshells. L: lumen; P: pharynx. (**B** and **C**) Cold suppression of the bcAM/actin-dependent polarity conversion. *pop-1*/β-catenin complex component, *let-502*/rho-dependent kinase, and *ifo-1*/intermediate filament regulator (142) serve as negative controls. (C) At 15°C, the apical domain/lumen is partially assembled in *arx-2(mild-RNAi)* embryos and the *unc-60(mild-RNAi)* larval polarity conversion is suppressed; no change in *pop-1(RNAi)* polarity and *ifo-1(RNAi)* lumenogenesis defects at 15°C. (**D** to **G**) *chc-1(b1025ts)* (22°C: late embryonic-, 15°C: no polarity conversion) enhances the *bcAM/actin(mildRNAi)* early-embryonic and larval polarity phenotypes at 22°C (D) and 15°C (E), respectively. Double mutant/RNAi generates a synthetic basolateral ectopic lumen (EL) phenotype at 15°C (F). (G) Left to right: failure of intestinal polarization and canal-lumen extension (GFP^+^ cyst) in arrested *chc-1(b1025ts);arx-2(RNAi)* embryo at 22°C; apical membrane blebs but no polarity defect in *chc-1(b1025ts)* at 15°C; enhancement of the *bcAM/actin(mild-RNAi)* polarity conversion by *chc-1(b1025ts)*; synthetic EL (asterisks) phenotype in *chc-1(b1025ts);actin(RNAi)* larval intestines at 15°C. (**H** to **K**) (H) *let-767(s2819);sDp3* (no polarity defects) induces polarity conversion in *unc-60, arx-2, cap-1(mild-RNAi)*, but not in *ifc-2(RNAi)* intestines (IFC-2: intermediate filament, used as negative control). (I) mild *actin* RNAi (no polarity defect) enhances the *rab-1(RNAi)* polarity conversion, but does not affect *rab-10(RNAi)* intestines (RAB-10: endocytic recycling component, used as negative control). Corresponding images in (J) and (K). Scaled-intensity RNAi was used throughout. Confocal and Nomarski/confocal images are shown. Values are mean ± SD; *n* ≥ 3, one-tailed *t* test is used: *P < 0.05; **P < 0.001; ***P < 0.0001. Scale bars, 10 μm.

The bcAM/actin-dependent intestinal polarity defect phenocopies the trafficking-dependent polarity defect (Fig. 5A; see the accompanying article) (*13*). Three lines of evidence further supported the hypothesis that branched-chain actin dynamics regulates membrane polarity via trafficking: (i) the previously noted reversibility of the *act-5(RNAi)–*dependent intestinal defects and larval lethality (not consistent with a structural morphogenesis defect) (*17*); (ii) the ability of actin depletion to induce apicobasal polarity conversion in the presence of intact junctions (Fig. 2J); and (iii) cold suppression of the bcAM/actin-dependent polarity conversion at temperatures expected to slow vesicle movement (Fig. 5, B and C).

To directly address the question if bcAMs/actins regulate polarity via trafficking, we examined genetic interactions between cytoskeleton- and vesicle-based polarity cues. Combining temperature-sensitive mutants (at the permissive temperature), balanced mutants (in the presence of the balancer) and mild RNAi conditions, we assessed interactions between animals with minor or no polarity defects (minimal depletion of either type of polarity cue). This approach avoided masking effects on membrane polarity (caused by strong depletion of these pleiotropic molecules) and allowed for the assessment of synthetic polarity phenotypes (induced by the combined depletion of cytoskeleton- and vesicle-based polarity cues).

CHC-1, the heavy chain of the post-Golgi vesicle coat clathrin, previously shown to function as polarity cue in the *C. elegans* intestine (*10*, *13*), was examined in the temperature-sensitive mutant *chc-1(b1025ts)* that arrests as embryo and fails to establish intestinal polarity at temperatures above 22°C but is viable and largely without polarity defects at 15°C. RNAi with bcAMs and actin was titrated in a wild-type background to produce (i) ≤^1^/_2_ progeny with early-embryonic lethality and intestinal polarity defects at 22°C (Fig. 5D), (ii) <^1^/_2_ progeny with larval basolateral ERM-1 mislocalization at 15°C (Fig. 5E), and (iii) no progeny with ectopic basolateral lumen formation at 15°C (Fig. 5F). *chc-1(b1025ts)* enhanced the bcAM/actin-dependent early-embryonic lethality and larval polarity conversion at the restrictive and permissive temperatures, respectively (Fig. 5, D, E, and G). All double *chc1(b1025ts) bcAM(RNAi)* conditions generated a synthetic ectopic lumen phenotype at 15°C (Fig. 5, F and G).

GSLs, Golgi- and post-Golgi endomembrane–based lipid raft components, also previously shown to function as polarity cues in the *C. elegans* intestine (*7*, *13*), were examined in the *let-767(s2819); sDp3* mutant that exhibits the polarity conversion/ectopic lumen phenotype in the absence of the duplication (*sDp3*) but appears wild type in its presence. RNAi with bcAMs was titrated to induce no or minimal basolateral ERM-1 displacement in a wild-type background. Double *let-767(s2819) bcAM(RNAi*) conditions generated a synthetic polarity phenotype in larval intestines (Fig. 5, H and J).

RAB-1 is an early secretory pathway molecule, located on pre-Golgi and Golgi endomembranes, whose depletion also induces polarity conversion in the *C. elegans* intestine (*13*)*. actin* RNAi, titrated to generate no polarity defect on its own, enhanced the basolateral mislocalization of ERM-1 on expanding membranes of larval intestines mildly depleted of RAB-1 (Fig. 5, I and K).

We conclude that the three components of the branched-chain actin machinery and actin itself functionally interact in apical domain positioning with the previously identified pre-Golgi–, Golgi-, and post-Golgi–based polarity cues (see the accompanying article) (*13*).

### Branched-chain actin dynamics confers apical directionality to pre- and post-Golgi vesicles during de novo polarized membrane biogenesis

**Fig. 6. F6:**
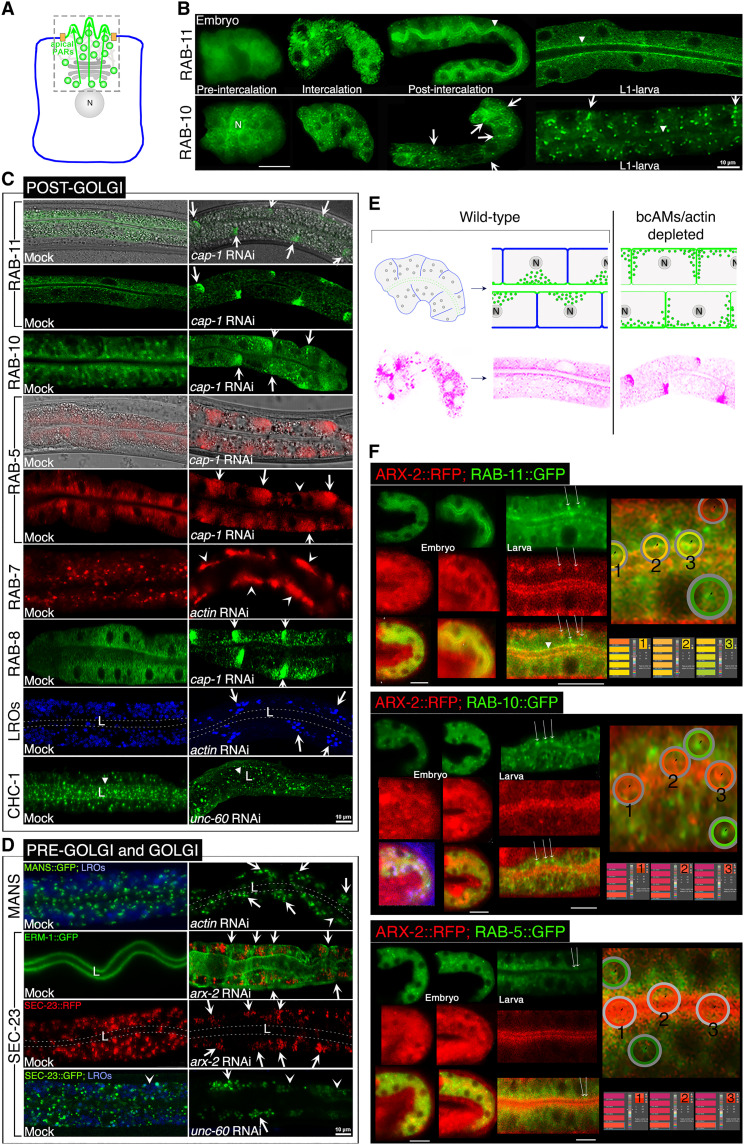
The apical positioning of pre- and post-Golgi vesicles depends on UNC-60, ARX-2, CAP-1, and actin during de novo polarized membrane biogenesis (see fig. S4 for additional results). (**A**) Proposed polarity model (side view of intestinal cell). The anterograde biosynthetic-secretory pathway is directionally constrained to asymmetrically insert the apical membrane. Box indicates endomembrane components proposed to acquire apical directionality. Apical endomembranes/plasma membranes/PARs: green; basolateral membrane: blue; junctions: orange; Golgi and cytoskeleton: gray; N: nucleus. (**B**) Developmental subcellular localization profile of *elt-2p*::RAB-11::GFP and *elt-2p*::RAB-10::GFP. Left to right: RAB-11 is recruited to vesicle membranes and moves to the apical/lumenal membrane (arrowhead) during intestinal polarity establishment; RAB-10^+^ vesicles remain mildly enriched at all membrane domains, including basolateral domains (arrows). (**C** and **D**) Post-Golgi, pre-Golgi, and Golgi endomembranes/vesicles fail to reach the apical membrane/lumen (L; dashed lines) and are mislocalized to basolateral membranes (arrows indicate some of the lateral, arrowheads some of the basal portions; Nomarski images reveal wild-type intestinal morphology). ERM-1::GFP; SEC-23::RFP double-labeled intestines in (D) show the concomitant basolateral mislocalization of the apical domain (ERM-1^+^) and of pre-Golgi vesicles (SEC-23+). (**E**) Schematic: deviation of apical vesicle directionality during bcAM/actin-impaired membrane polarization. Green: apical vesicles and apical membranes; blue: basolateral membranes. N: nucleus. Lower panels trace RAB-11 vesicles from (B) and (C) (pseudocolored to pink). (**F**) Spatiotemporal coexpression of ARX-2 with RAB-11^+^, RAB-10^+^, and RAB-5^+^ vesicles during intestinal polarity establishment. Merged images are measured by the Eyedropper tool: overlap = yellow (right; high magnification). Cytoplasmic overlap (early-embryonic intestine, left), but no vesicle-based ARX-2 overlap (some vesicles indicated by long arrows), except with apical membrane–close RAB-11^+^ vesicles (full arrowhead). Confocal/confocal-Nomarski overlay images of embryonic or larval intestinal cells are shown. Labeling by translational fluorescent fusion proteins, except for autofluorescent lysosome-related organelles (blue). *elt-2p* or *vha-6p* restrict the ubiquitous expression of vesicle components to the intestine. Scale bars, 10 μm.

If branched-chain actin dynamics asymmetrically routes vesicles with apical membrane components to the growing membrane to insert the nascent apical domain [Fig. 6A; proposed polarity model; compare (*13*)], the apical directionality of biosynthetic vesicles should depend on bcAMs and actin during de novo membrane biogenesis and polarity establishment. The polarization of vesicles (and nuclei) toward the future apical domain was indeed noted to predate membrane polarity establishment in the *C. elegans* intestine (*26*, *28*). It remains difficult in any in vitro or in vivo system to visually track the directionality of biosynthetic trafficking, since polarized cargo becomes successively enriched from the endoplasmic reticulum (ER) to the plasma membrane, while vesicle-membrane and coat components are consecutively recruited and shed (*29*). To map wild-type vesicle trajectories through polarity establishment in *C. elegans* intestinal cells, we directed RAB-11::GFP and RAB-10::GFP, marking vesicles with presumed apical versus basolateral directionality (*30*), to the intestinal E-cell lineage by the early-acting intestine-specific promoter *elt-2*. Consistent with RAB-11’s proposed function in apical domain positioning (*13*), RAB-11::GFP was recruited to vesicles in intercalating early-embryonic intestinal cells, and GFP^+^ vesicles became enriched at the apical plasma membrane during polarity establishment. In contrast, the spatiotemporal subcellular localization of RAB-10::GFP^+^ vesicles remained largely unaltered, with mild enrichment at all, specifically at basolateral, membrane domains (Fig. 6B).

We next assessed whether the positionings of these and other pre-, and post-Golgi vesicle populations was dependent on bcAMs and actin in late-embryonic/early-larval intestinal cells, a time of substantial net polarized membrane addition (fig. S3). Mild depletion of each bcAM or actin, but not of *sma-1*/spectrin (another component of the apical cytoskeleton; used as control), resulted in the basolateral mislocalization of apical subpopulations of RAB-11^+^ and RAB-10^+^ recycling, RAB-5^+^ early-endocytic, RAB-7^+^ late-endocytic/lysosomal, RAB-8^+^ presumed secretory vesicles, and of lysosome-related organelles (LROs) in intact-appearing intestinal cells without detectable vesicle biogenesis defects (Fig. 6C and fig. S4A). Mild *bcAM/actin* RNAi also prevented the apical enrichment of clathrin-coated (CHC-1+) post-Golgi vesicles, tubulated MANS^+^ Golgi membranes, and mislocalized COPII-coated (SEC-23+) pre-Golgi vesicles to basolateral domains (Fig. 6D and fig. S4B).

We concluded that UNC-60, ARX-2, CAP-1, and actin are required for the apical positioning of multiple pre- and post-Golgi vesicles during net polarized membrane expansion, including vesicles marked by polarity cues (e.g., RAB-11, CHC-1, and SEC-23) (*13*) and LROs, previously noted to move to the apical domain in the *C. elegans* intestine (*31*). The results were consistent with the hypothesis that branched-chain actin dynamics asymmetrically directs anterograde vesicle trajectories from the ER to the apical membrane domain during this domain’s biogenesis [Fig. 6, A and E; compare (*13*)]. The findings could also suggest that distinct and presumed specialized vesicle populations are co-opted for the delivery of apical membrane components during de novo polarized membrane biogenesis.

To explore if bcAMs might assemble actin on specific vesicles for forward propulsion, we also searched for colocalization of bcAMs and post-Golgi vesicle markers during intestinal development. This analysis failed to detect any direct overlap with the tested vesicle markers, except for ARX-2::RFP colocalization at the apical membrane with RAB-11::GFP^+^ vesicles (Fig. 6F and fig. S4, C and D). These findings suggested that bcAMs, rather than propelling specific vesicles, e.g., via actin plumes, might direct entire vesicle populations by powering broader actin structures.

### ACT-5 functionally interacts with other actin isoforms in intestinal membrane polarity

To understand how ACT-5/actin might route vesicles through the intestinal cytoplasm to the apical domain, we returned to the question if ACT-5, thought to be exclusively localized at the apical domain (*17*), was the only actin in the *C. elegans* intestine and was therefore mediating actin’s polarity function. Several lines of evidence suggested that this was not the case: (i) Single-cell combinatorial indexing RNA sequencing (sci-RNA-seq) detects *act-1*, *act-2*, *act-3*, and *act-4* expression in the intestine, albeit at low levels (fig. S6); (ii) LifeACT labeling of F-actin was not restricted to the apical intestinal membrane [Fig. 4 and (*23*)]; (iii) *actin* RNAi (also known as *act-5* RNAi, commonly used with the intent to target *act-5* but in fact targeting all actins) and *act-5-LE17bp *3′UTR RNAi (possibly targeting other actins), but not *act-5(ok1397)* or *act-5 *3′UTR RNAi, affected intestinal polarity [see above, fig. S2B, and (*17*)].

**Fig. 7.**
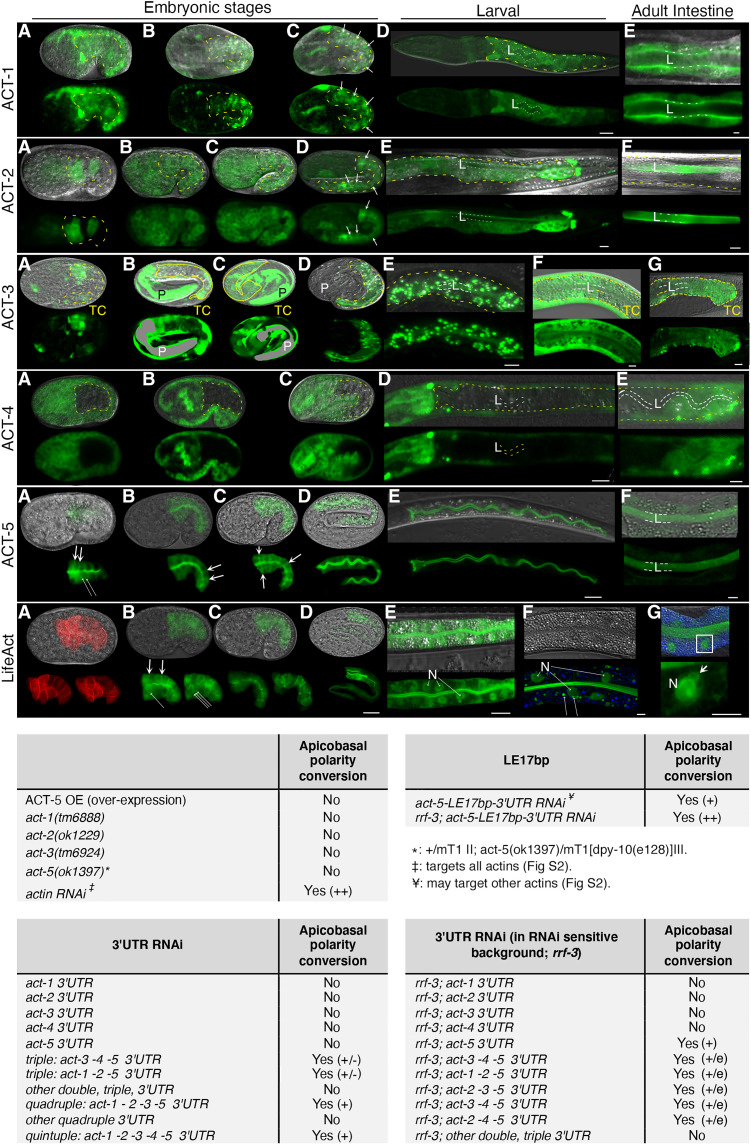
Multiple actin isoforms are expressed in the *C. elegans* intestine and function with ACT-5 in polarity. Developmental intestinal expression profile of ACT-1, ACT-2, ACT-3, ACT-4, ACT-5, and LifeACT. ACT-1, ACT-2, ACT-3, and ACT-4 brightness is increased pre- and post-image acquisition to visualize low expression. ACT-1, ACT-2, and ACT-4 are dominant body wall-muscle actins (fig. S5: extra-intestinal expression). Pre-intercalation and intercalating early-embryonic, post-intercalation late-embryonic, expanding L1-larval, and mature adult intestines are shown from left to right. Some intestines are outlined by yellow dashes; some of the basolateral membranes are indicated by arrows. L: lumen or dashed line. **ACT-1::GFP:** (**A** to **E**) Cytoplasmic, pan-membranous (apico-basolateral: B and C), and increasingly apical location (C to E). **ACT-2::GFP:** (**A** to **F**) Cytoplasmic, pan-membranous (apico-basolateral) (B to D), and increasingly apical location (D to F). **ACT-3::GFP:** (**A** to **C**, **F**, and **G**) TC: transcriptional *act-3p::*GFP. (**D** and **E**) Translational *act-3p*::ACT-3::GFP is increasingly located at the apical domain and at vesicles (expression induces L3-larval lethality). Pharynx = P is grayed out in (B) and (C). **ACT-4::GFP:** (**A** to **D**) No ACT-4 expression in the embryonic and early-larval intestines. (**E**) Faint cytoplasmic localization in mature posterior intestine. **ACT-5::GFP (dominant intestinal actin):** (**A** to **F**) Cytoplasmic, pan-membranous (apico-basolateral) (A to D), and increasingly apical location (A to F). Long thin arrows: ACT-5 patches. **Intestinal LifeACT::mCherry and LifeACT::GFP:** (**A** to **G**) Cytoplasmic, pan-membranous, and increasingly apical location. (A) Note cytoplasmic mesh and (A to C) patches (long thin arrows). Two confocal sections are shown in (A to C). Predominantly apical, and peri-vesicular [long thin arrows in (F)] and perinuclear localization (N: nucleus) in larval (E) and adult intestine (F and G). (G) Nuclear appendix (arrow). Blue: autofluorescent LROs. Confocal sections of intestinal midplane and Nomarski/confocal overlay images are shown. Scale bars, 10 μm. **Actin isoforms interact with ACT-5 in polarity:** apicobasal polarity conversion was assessed via basolateral ERM-1::GFP mislocalization (Yes/No). (+/−) indicates very mild polarity defect, and (+/e) indicates mild enhancement of polarity defect.

We generated isoform-specific ACT-1, ACT-2, ACT-3, and ACT-4 translational GFP fusions, in addition to ACT-5::GFP, and analyzed their developmental expression pattern. This analysis revealed that (i) ACT-1, ACT-2, ACT-3, and ACT-4 are also expressed in the intestine (in addition to their extra-intestinal expression; Fig. 7 and fig. S5); (ii) ACT-5—as well as ACT-1, ACT-2, and ACT-3—is also located in the cytoplasm and at future basolateral membranes in the early-embryonic intestine before and during polarization; (iii) ACT-5—and ACT-1, ACT-2, and ACT-3—undergoes a synchronous basolateral-to-apical polarity shift during intestinal polarity establishment (ACT-4::GFP is only detected in the mature intestine).

To determine if ACT-5 was even necessary for polarity, we titrated *act-5 *3′UTR RNAi conditions up, avoiding strong ACT-5 depletion expected to displace ERM-1 from the membrane and thus impede the ability to assess ERM-1’s polarized position at the membrane. Although unable to induce polarity conversion in wild-type, *act-5* 3′UTR RNAi mispositioned ERM-1 to the basolateral membrane in an RNAi-sensitive *rrf-3* background (table S1, Fig. 7). This background also augmented the penetrance of apicobasal polarity conversion in *act-5-LE17bp(3′UTR-RNAi)* and *actin(RNAi)* larval intestines. We concluded that *act-5* is indeed necessary, but that additional actin isoforms may be needed, for actin’s full polarity function. The results also validated the 3′UTR RNAi approach.

To assess the requirement of non–ACT-5 actin isoforms for intestinal polarity, we examined *act-1(tm6888)*, *act-2(ok1229),* and *act-3(tm6924)* germline deletion alleles that remove those actin isoforms that copied the basolateral-to-apical ACT-5::GFP polarity shift during polarity establishment. These presumed null alleles (fig. S6B) are homozygous viable and superficially wild type, consistent with expected redundancies among actin isoforms (fig. S7). All three alleles failed to mislocalize ERM-1::GFP to the basolateral intestinal membrane, yet caused mild, isoform-specific apical domain biogenesis defects (fig. S8). We concluded that, if isoforms other than ACT-5 function in polarity, these isoforms operate redundantly, require ACT-5, and/or require more than one non–ACT-5 isoform for their polarity function.

The genetic analysis of actins has been complicated by large families of almost identical, redundant actin isoforms and the susceptibility of filament assembly to imbalances in actin stoichiometries. To deplete non–ACT-5 isoforms only mildly yet distinguish isoform-specific functions, we trialed a combinatorial 3′UTR-directed RNAi approach and targeted the non–ACT-5 isoforms *act-1*, *act-2*, *act-3*, and *act-4* separately and together (transcripts are closely similar, while 3′UTRs diverge; fig. S6A). All single, double, and triple combinations of *act-1*, *act-2*, *act-3*, and *act-4* 3′UTR RNAi, with or without enhanced RNAi conditions (*rrf-3*), failed to mislocalize ERM-1 to the basolateral membrane or to generate obvious other phenotypes (table S1, Fig. 7). In marked contrast, quadruple *act-1 + act-2 + act-3 + act-4 *3′UTR RNAi caused embryonic and L1-larval arrest with pronounced body morphology defects, yet also failed to affect polarity. However, quintuple *act-1 + act-2 + act-3 + act-4 + act-5 *3′UTR RNAi induced intestinal polarity conversion (*act-5 *3′UTR RNAi alone does not induce polarity conversion, see above) and enhanced polarity conversion in intestines treated with *act-5 *3′UTR RNAi in an *rrf-3* RNAi-sensitive background or with *act-5-LE17bp* ′UTR RNAi (Fig. 7; note that *act-5* produces >85% of intestinal actin, suggesting that the effect of *act-1*, *act-2*, *act-3*, and *act-4* 3′UTR RNAi on the total amount of actin is minor; fig. S6D and Fig. 7). These results revealed that non–ACT-5 actin isoforms (i) contribute to ACT-5’s function in intestinal polarity, (ii) likely require ACT-5 for their function in polarity, and (3) function redundantly in intestinal polarity and extra-intestinal morphogenesis or require several isoforms for these functions. The results also validated a multiple (up to quintuple) RNAi approach.

To distinguish between the contributions of different non–ACT-5 isoforms to intestinal polarity, we also asked if the combined depletion of two or more non–ACT-5 isoforms could induce polarity conversion in *act-5(3′UTR-RNAi)* intestines. All triple 3′UTR RNAi combinations with either *act-1*, *act-2*, *act-3*, or *act-4* and double 3′UTR RNAi with *act-1 + act-2* and *act-3 + act-4*, but no other combination, induced mild basolateral ERM-1 mislocalization in an *act-5(3′UTR-RNAi)* background (table S1 and Fig. 7).

We conclude that ACT-5 is required for intestinal polarity, but that at least two more, almost identical actin isoforms may be needed in addition to ACT-5 for actin’s full polarity function. The concomitant basolateral-to-apical shift of ACT-5 and of ACT-1, ACT-2, and ACT-3 in polarizing cells furthermore suggested that these different actin isoforms might contribute to an F-actin structure that confers apical directionality to vesicle trajectories during polarity establishment.

### F-actin moves from the basolateral to the apical domain during polarity establishment

**Fig. 8.**
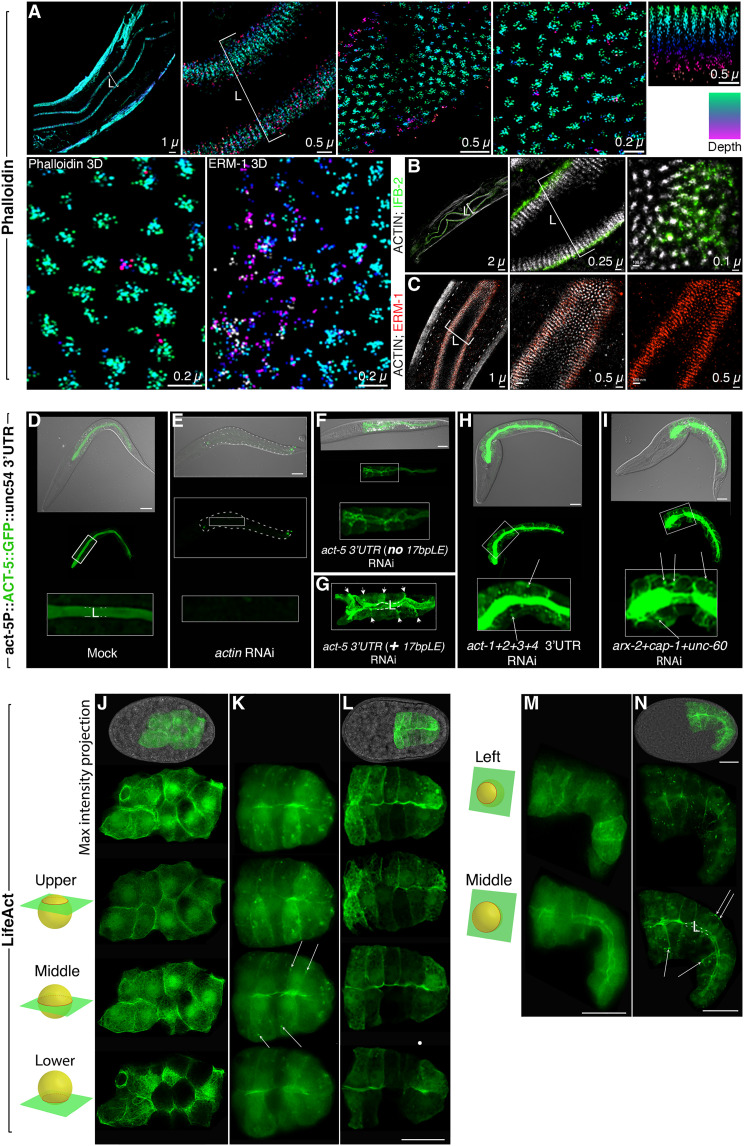
Actin determines its own polarity. (**A** to **C**) **Phalloidin panels:** the submembranous apical F-actin assembles into microvillar bundles. (A) 3D STORM (stochastic optical reconstruction microscopy) analysis of apical/lumenal intestinal membrane. L1-larva mid-portion, outlined by muscle actin (left). Upper row, left to right: increasing magnification of microvillar F-actin. Color-coding (right): green = top, purple = root. L: lumen. Lower row: microvillar cross-sections: F-actin versus ERM-1. F-actin is labeled with Alexa Flour 647–phalloidin. (B and C) Double-color STORM with Cy3B anti–IFB-2 and Cy3B anti–ERM-1. (**D** and **E**) ***act-5p*::ACT-5::GFP::*unc-54*-3′UTR panels:** ACT-5’s polarity depends on itself, other actin isoforms, and bcAMs. Apical/lumenal *act-5p*::ACT-5::GFP::*unc-54-*3′UTR is knocked down by *actin* RNAi. (**F** and **G**) *act-5 * 3′UTR RNAi and *act-5-LE17bp* 3′UTR RNAi, targeting the 3′UTR (missing in *act-5p::*ACT-5::GFP::*unc-54 *-3′UTR; figs. S2B and S6A), fail to knock down *act-5p*::ACT-5::GFP::*unc-54-*3′UTR (fig. S8, M to O) and mislocalize it to basolateral membranes (arrows). (**H** and **I**) Quadruple *act-1 *to* act-4* 3′UTR RNAi and triple bcAMs RNAi mislocalize ACT-5 to basolateral membranes. Note additional ACT-5 displacement to: the cytoplasm (F to I), a cytoplasmic mesh, and patches (long arrows). Body morphology defects in (E), (H), and (I) [note “big-head” in (H) and (I)], demonstrate efficacy of 3′UTR RNAi. Confocal/Nomarski-confocal overlay images of L1 larvae are shown. Dashed line (E): intestine. (**J** to **N**) **LifeACT panels:** a cytoplasmic and cortical F-actin mesh shifts to the apical domain during intestinal polarity establishment. F-actin is labeled by *elt-2p-*LifeACT::GFP. Nomarski/confocal overlay images in top panels, confocal projections beneath, followed by sections. Schematic spheres: focal planes. (J) Pre-intercalation (pre-bean stage embryo): F-actin in cytoplasmic mesh, perinuclear, and pan-membranous (cortical). Middle section demonstrates that mesh traverses the cytoplasm. (K and L) Intercalating intestine (early bean stage). F-actin increase at the midline (future apical/lumenal domain). Long arrows: F-actin patches. (M and N) Post-intercalation (comma-stage): F-actin enrichment at the apical domain.Long arrows: F-actin patches. Images in (J), (L), and (N) are 1× deconvolved (2D-blind; numerical aperture 1.4; refraction 1.51; iteration 1; thickness: thin; noise: clear). Scale bars, 10 μm.

To visually identify a filamentous actin (F-actin) structure suitable to give direction to vesicles during membrane polarization in the developing *C. elegans* intestine, we searched for F-actin assemblies with documented roles in directional trafficking. Neither by phalloidin, nor by fluorophore fusions with ACT-5 or other actin isoforms, nor by LifeACT, could we detect obvious actin cables that might serve as long-range tracks to move vesicles via atypical myosins (*32*). However, short-range, apical membrane–close F-actin tracks, implicated in apical secretion in other epithelia (*33*, *34*), might not have been resolved by conventional microscopy. To image the submembranous apical cytoskeleton at molecular resolution, we examined L1 larvae by stochastic optical reconstruction microscopy (STORM) and used phalloidin to detect all actins (Fig. 8, A to C). This approach resolved single actin filaments in apical microvilli at nanometer resolution (average, 26 filaments/microvillus; Fig. 8A), extending high-resolution confocal and immunoelectron microscopy studies (*35*). Double-color STORM revealed that ERM-1 traces actin filaments into the microvilli (Fig. 8, A to C), but neither phalloidin^+^ nor ERM-1^+^ tracks were found to emanate from a peri-lumenal F-actin belt postulated to root microvillar actin. Unexpectedly, no such belt, nor junctional F-actin, was identified. Instead, double-color STORM of F-actin and IFB-2 revealed that actin filaments were rooted in a peri-lumenal intermediate filament belt (Fig. 8B).

bcAM-powered F-actin networks can produce force with and without myosins, and actin filament turnover (treadmilling) can self-organize to create directional momentum that propels membranes forward (*19*). We next examined actin’s ability to determine its own polarity, i.e., its polarization to the apical membrane. We took advantage of the transgenic ACT-5::GFP, expressed from a construct missing the *act-5* 3′UTR (*act-5p::*ACT-5::GFP*::unc54-*3′UTR; Fig. 8, D to I). The depletion of endogenous ACT-5 by *act-5* 3′UTR RNAi (figs. S2B and S6A) interfered with the polarity of transgenic *act-5p::*ACT-5::GFP*::unc54-*3′UTR, whose expression was not decreased since it misses the *act-5* 3′UTR (Fig. 8D; expression levels were, conversely, increased; see fig. S8M and legend for further discussion of this only partially rescuing transgene). In contrast, *actin* RNAi, used as control, effectively removed *act-5p::*ACT-5::GFP*::unc54-*3′UTR (Fig. 8E). Both *act-5 *3′UTR and *act-5-LE17bp *3′UTR RNAi mislocalized *act-5p::*ACT-5::GFP*::unc54-*3′UTR to the cytoplasm and to basolateral membrane domains (Fig. 8, F and G). Moreover, single and combinatorial *act-1*, *act-2*, *act-3*, and *act-4 *3′UTR RNAi also mislocalized ACT-5::GFP (although not ERM-1::GFP, see above) to the basolateral membrane, to the cytoplasm, and to cytoplasmic and cortex-associated patches (Fig. 8H). Similarly, the single and combined depletion of UNC-60, ARX-2, and CAP-1 displaced ACT-5::GFP to basolateral membrane domains and to a mesh-like structure in the cytoplasm (Fig. 8I). We concluded that ACT-5, other actin isoforms, and the three bcAMs are nonredundantly required for ACT-5’s own polarization to the apical domain in the developing intestine. Furthermore, non–ACT-5 actin isoforms are required to polarize ACT-5. Together, these findings were consistent with a bcAM-powered vectorial F-actin network that moves through the cytoplasm and along the cortex to position the nascent apical domain, and that might combine several, almost identical actin isoforms with ACT-5. However, bcAMs/actins could contribute in other ways to the positioning of the apical domain, a domain that also supports the de novo nucleation of F-actin that could be displaced to basolateral domains during polarity conversion.

To search for a putative vectorial F-actin network and track its dynamics during cell polarization, we used positional recording and time-lapse imaging of LifeACT, the most sensitive and actin isoform unbiased F-actin tracer (*36*). In not yet polarized cells of the pre-intercalation embryonic intestine, high-resolution confocal microscopy of LifeACT::GFP revealed a cytoplasmic F-actin mesh, enriched at all sides of the membrane (Fig. 8J). At the time of polarity establishment, this F-actin mesh appeared to move toward the midline (future apical domain/position of the lumen), forming transient, cortex-enriched F-actin patches in cells of the intercalating intestine, with a successive increase of F-actin at the emerging apical domain (Fig. 8, K and L; see fig. S3 for schematic of intestinal intercalation). Apical F-actin enrichment continued in postmitotic but still growing intestinal cells in the post-intercalation late-embryonic and larval intestine (Fig. 8, M and N; see Fig. 7 for developmental intestinal subcellular localization profile of ACT-1 to ACT-5 and LifeACT). These findings were consistent with a directional F-actin shift across the cytoplasm and along the cortex to the apical domain during this domain’s coincident biogenesis and positioning. However, these experiments could not distinguish whether F-actin, rather than moving to the apical domain, was successively excluded from the basolateral, and newly nucleated at the apical, domain.

**Fig. 9.**
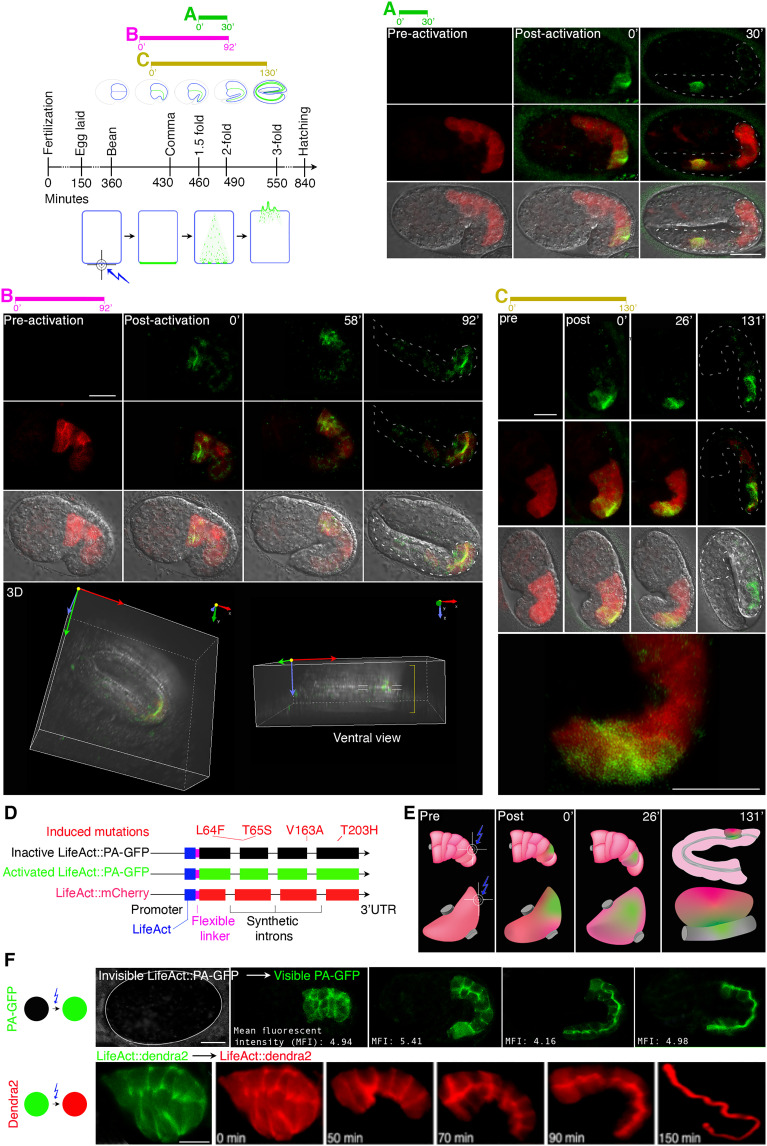
F-actin moves across the cell and along the cortex toward the nascent apical domain during de novo polarized membrane biogenesis (see fig. S9 for additional results). Upper left schematic: Colored horizontal bars indicate timing of recording after photoactivation (=minute 0). Schematic beneath shows subcellular PA (photoactivatable)–GFP tracking after targeting the basal membrane (lightning bolt). See fig. S9 for photoconvertible Dendra2. (**A**) LifeACT::PA-GFP (not visible pre photoactivation) is activated at the basolateral membrane angle of a posterior intestinal cell (post-intercalation), using LifeACT::mCherry (visible) as a guide. At minute 30, PA-GFP (green) is retrieved at the apical domain. (**B**) Lateral membrane of the E2Lintestinal cell is photoactivated during intercalation (see fig. S3 for intestinal morphogenesis). Fifty-eight and 92 min later, LifeACT::PA-GFP has shifted toward the midline (future apical domain). Rotated 3D images: activated LifeACT::PA-GFP at the apical membrane at minute 92. (**C**) Basal domains of two posterior cells are photoactivated during intercalation. LifeACT::PA-GFP has shifted to the apical membrane post-intercalation (131 min later; image is rotated). (**D**) LifeACT constructs. Four mutations were introduced to make the GFP photoactivatable. A flexible linker region distances the fluorophore from the protein. Synthetic introns are optimized for brighter expression. (**E**) Schematic, tracking events shown in (C) (embryo is rotated 90^o^ counterclockwise). Lightning bolt: area targeted. LifeACT::PA-GFP dynamics (green) are followed from the basolateral to the apical membrane (lumen) in one cell (lower panel). (**F**) LifeACT photoactivation and LifeACT::Dendra2 photoconversion of whole pre-intercalation intestine (left), with subsequent tracking of fluorophores (up to threefold embryo for LifeACT::PA-GFP; up to L1-larva for LifeACT::Dendra2). Only activated/converted, not newly made, LifeACT::PA-GFP (green) and LifeACT::Dendra2 (red) are shown. Note that the quantity of activated LifeACT::PA-GFP remains constant. Confocal and Nomarski/confocal overlay images are shown throughout. Intestines are outlined by dashed lines in (A) to (C). Scale bars, 10 μm.

**Fig. 10.**
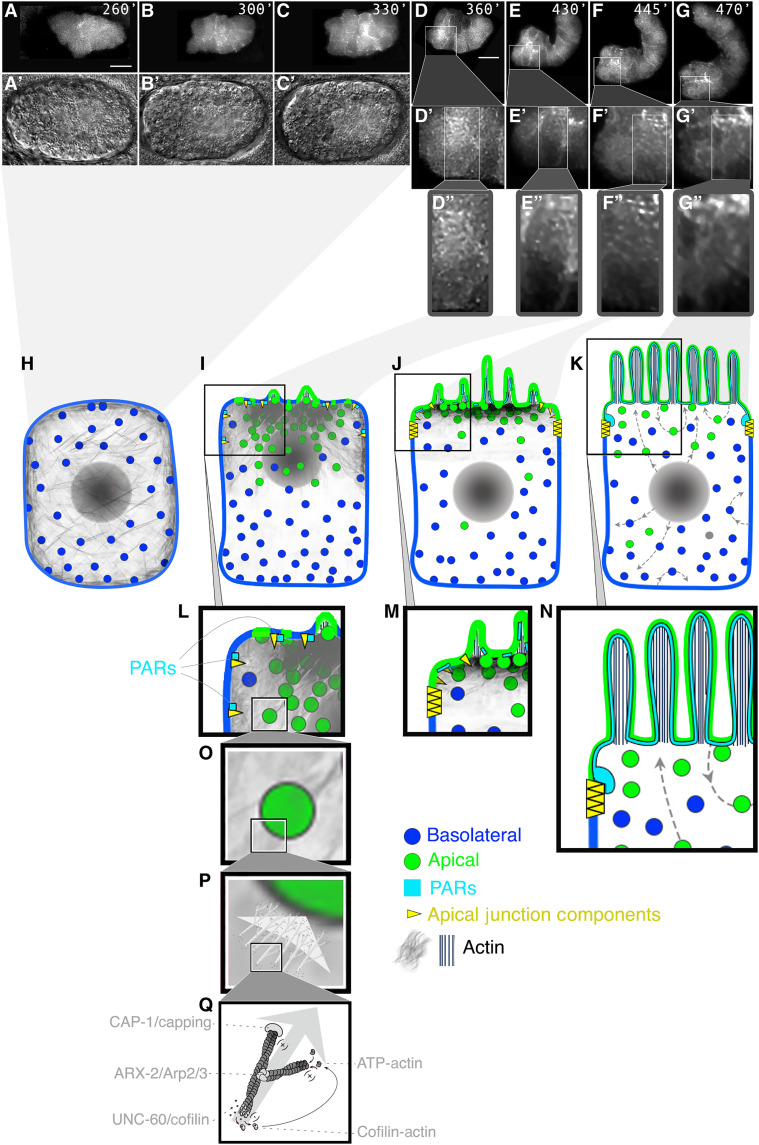
Proposed polarity model. A bcAM-powered vectorial F-actin network confers asymmetric directionality to anterograde vesicle trajectories that insert the nascent apical domain into the growing membrane. (**A** to **G"**) Time-lapse imaging of the basolateral-to-apical LifeACT::GFP shift during intestinal polarity establishment. Pre-bean (A to C), bean (D), comma, and 1.5-fold stage embryo (E to G). Anterior: left, dorsal: down. Cytoplasmic and cortical F-actin (A to E) shifts (C to G) to the nascent apical domain (lumen) (G to G″). Confocal sections and Nomarski/confocal overlay images are shown. A single cell (E2L), intercalating in (D), is magnified in (D′) to (G″). Scale bars, 10 μm. (**H** to **N**) Schematic of an intestinal cell: polarization proceeds from left to right, magnification from top to bottom. (H to K): Cytoplasmic and cortex-associated F-actin (gray mesh; H and I) shifts to the apical domain (I to K), conferring directionality to biosynthetic vesicles [nucleus also moves (*26*, *116*)]. Vesicles may attach to F-actin via the previously identified vesicle-based polarity cues [see the accompanying article (*13*)]. Green: apical plasma membranes/vesicle membranes; blue: basolateral plasma membranes/vesicle membranes. (L to N) Apical PAR (turquoise) and junction components (yellow) are recruited to the apical domain concomitantly with, or subsequently to, the delivery of apical membrane components and secure the expanding apical domain by junction assembly (J and M). The mode of PAR recruitment is unknown, and only the demonstrated lateral-to-apical cortical PAR movement is shown (*15*, *26*). (K and N) F-actin becomes almost fully restricted to microvillar bundles. The polarization of the plasma membrane allows for apicobasal sorting (arrows). (**O** to **Q**) Branched-chain actin dynamics. Actin filament modulation via UNC-60/cofilin (depolymerization), CAP-1 (capping), and ARX-2/Arp2/3 (branch nucleation) induces vectorial F-actin dynamics that either directly or indirectly (via higher-order polymeric F-actin structures) moves vesicles to the nascent apical domain (compare Fig. 2, P and Q).

To directly address whether F-actin physically moved across the polarizing cell, we generated photoactivatable LifeACT::PA-GFP and photoconvertible LifeACT::Dendra2 fusion proteins and expressed them in the developing intestine (Fig. 9 and fig. S9). Activated LifeACT::PA-GFP (green) or converted LifeACT::Dendra2 (red) only detect F-actin generated before, but none generated after, activation or conversion. We examined the location of the activated GFP and the converted Dendra2 during intestinal development in single intestinal cells and in the whole organ by time-lapse confocal microscopy (compare Fig. 10, A to G″, for time-lapse images of nonmodulated LifeACT::GFP). The photomodulated fluorophores traced the movement of LifeACT through the cytoplasm and along the cortex to the apical domain in polarizing early-embryonic, and in postmitotic but still expanding late-embryonic/larval, intestinal cells (Fig. 9, fig. S9, and movies S1 and S2). Activated LifeACT::PA-GFP (green) and converted LifeACT::Dendra2 (red) did not disperse randomly to other areas of cellular F-actin, as demonstrated by the counter labeling of newly generated F-actin by LifeACT::mCherry (red) or nonconverted LifeACT::Dendra2 (green), respectively. We conclude that F-actin physically moves toward the nascent apical domain throughout de novo polarized membrane biogenesis in single cells of the developing intestine. These findings are consistent with a scenario where the apical shift of a dynamic F-actin network confers directionality to biosynthetic vesicle trajectories that insert the nascent apical domain into the growing membrane [see our accompanying article (*13*)].

## DISCUSSION

### Branched-chain actin dynamics determines apicobasal membrane polarity

Here, we identify UNC-60/cofilin, ARX-2/Arp2/3 component, CAP-1/capping (designated bcAMs), and actin itself as intracellular polarity cues that act upstream of the membrane-based core polarity cues in the process of epithelial membrane polarization. Branched-chain actin dynamics is required to establish and maintain the position of the apical domain (lumen) throughout de novo polarized membrane biogenesis in the developing *C. elegans* intestine: The loss of any of its component results in apicobasal polarity conversion in the expanding larval intestine and in a failure to partition polarized membrane domains in the early-embryonic intestine during polarity establishment. We suggest that bcAMs operate in this polarity function from a transient cytoplasmic and pan-membranous location in cells poised to polarize, where they power a basolateral-to-apical F-actin shift that asymmetrically drives anterograde vesicle trajectories to the nascent apical domain. A scaled-intensity RNAi approach allowed us to separate actin’s role in polarized membrane biogenesis from its many other essential cellular functions. The independent identification of all three components of branched-chain actin dynamics in unbiased tubulogenesis screens by the same apicobasal polarity conversion phenotype strongly endorses the requirement of branched-chain actin dynamics for this specific polarity function.

### Actin positions the apical membrane domain by polarizing intracellular trafficking

The following evidence suggests that bcAMs and actin function in polarity by conferring apical directionality to anterograde vesicle trajectories to position the apical domain, rather than by their canonical functions in apical membrane or junction modeling: (i) the bcAM/actin-dependent polarity defect phenocopies the polarity defect induced by interference with vesicle-based polarity cues that direct biosynthetic trafficking to the apical domain (*13*); (ii) bcAMs/actins genetically interact with vesicle-based apical polarity cues in polarity; (iii) all components of branched-chain actin dynamics are required to direct pre- and post-Golgi vesicles to the apical domain during de novo polarized membrane biogenesis; (iv) both systems (actin dynamics and trafficking) are required to polarize PAR-3, PAR-6, and PKC-3 during polarity establishment; (v) the components of both systems, branched-chain actin dynamics (bcAMs; several actin isoforms; the actin network itself), and vesicles shift from the cytoplasmic and basolateral to the apical domain during polarity establishment; (vi) a bcAM-driven F-actin network physically moves to the nascent apical domain during net polarized membrane expansion. We therefore propose that branched-chain actin dynamics determines membrane polarity by asymmetrically directing the biosynthetic-secretory pathway from the ER to the plasma membrane to insert the nascent apical domain, thereby partitioning the growing membrane into apical and basolateral domains. This proposition supports the idea that the directional delivery of bulk membrane can determine epithelial membrane polarity (*13*) and provides a mechanism for a directional regulation of membrane delivery that is independent of cargo sorting to already polarized target domains [see our accompanying article (*13*)].

### A vectorial F-actin network, powered by bcAMs, confers apical directionality to vesicles

The classical mode of actin-guided vesicle movement is the MyoV-dependent forward propulsion of vesicles along long-range cytoplasmic F-actin cables with predetermined directionality (*32*). Branched- or straight-chain actin dynamic–dependent apical trafficking, guided by short, apical domain–associated actin tracks, has also been described: the Arp2/3-dependent trafficking of the Notch ligand Delta into apical microvilli of *Drosophila* sensory organ precursors (*37*); the formin-dependent apical trafficking that expands but does not position the lumen in *Drosophila* tubular epithelia (*33*); apical secretion in the mouse pancreas (*34*). We failed to identify F-actin cables for directed vesicle propulsion, nor did we find apical domain–linked actin tracks in the *C. elegans* intestine, but instead, we identified a dynamic vectorial F-actin network. Straight- or branched-chain powered F-actin networks can also move vesicles and organelles and may operate with greater flexibility than predetermined tracks, responding to different modes of vesicle dynamics. For instance, the formin-dependent propulsion of RAB-11^+^ vesicles to the cortex of nonpolarized mouse oocytes (*38*) can be directionally altered by vesicle density to support asymmetric spindle positioning (*39*). Mitochondria, whose directional movement during cell division is Arp2/3-dependent in yeast (*40*), can both be “shuffled” and spatially directed in HeLa cells by a combination of a subcortical actin meshwork and organelle-based actin clouds with the ability to transform into comet tails (*41*). We suggest that the bcAM-powered F-actin network in the *C. elegans* intestine transiently acquires apical directionality in polarizing cells, with apical membrane–biosynthetic vesicles connecting to it via those vesicle components previously identified as apical polarity cues [e.g., COPI, COPII, and clathrin coat and coat assembly components (*13*)].

### Cytoplasmic actin structure and dynamics

#### Actin mesh

The vectorial F-actin network identified in polarizing cells of the *C. elegans* intestine is of a mesh-like structure that appears better suited to moving groups of vesicles than single vesicular carriers, typically shuttled along F-actin cables by myosins (*32*) or directly propelled by F-actin plumes, comets (*42*), contractile coats, or rings (*43*). Unlike the formin-dependent static and isotropic F-actin mesh of similar appearance that maintains the polarity of microtubules in *Drosophila melanogaster* oocytes (*44*), the bcAM-dependent F-actin mesh in *C. elegans* intestinal cells is dynamic and anisotropic. It remains to be shown whether this mesh is contractile, and, if so, if it is retracted from or pushed to the apical domain and if its contractility is myosin-dependent. Contractility could depend on *unc-60*/cofilin activity alone, as shown in starfish oocytes, where the disassembly-driven contractility of a nuclear actin mesh becomes asymmetrically determined by cortex affixation (*45*, *46*). Little is known about the role of highly similar actin isoforms in such polymeric F-actin structures. We here find that several other, almost identical, intestinal actin isoforms recapitulate the basolateral-to-apical shift of ACT-5, the dominant *C. elegans* intestinal actin, and are nonredundantly required to optimize F-actin’s directional shift and its polarity function. These actin isoforms may therefore be functionally relevant structural F-actin mesh components.

#### Actin treadmilling

Treadmilling, powered by branched or straight-chain filament assembly modifiers, is the conserved mode of actin’s self-organization into vectorial F-actin networks (*18*, *19*), used for directed forward movement from bacteria and parasitic fungi to human cells (*47*). bcAM-powered treadmilling can directly generate force on intracellular organelles (*48*, *49*), and it drives lamellipodial membrane protrusions to direct cell migration, where it strictly depends on stoichiometries between bcAMs, especially Arp2/3 and capping proteins (*20*). We find the stoichiometric requirement of the distinct roles of UNC-60/cofilin, ARX-2/Arp2/3, and CAP-1/capping in filament assembly/disassembly reflected in their genetic interactions during polarized membrane biogenesis in *C. elegans* intestinal cells, consistent with a shared function of these three bcAMs in polarity via actin treadmilling. However, a physical basolateral-to-apical F-actin shift, endorsed by the movement of photomodulated LifeACT fluorophores, suggests that filaments also assemble into a dynamic higher-order polymeric F-actin structure, yet to be characterized [treadmilling itself, with continuous disassembly at one filament end and reassembly at the other end, only gives the appearance of filament movement (*19*)].

#### Actin biomechanics

Cytoplasmic actin flow or streaming, first linked to organelle movement in plants (*50*), is another actin-dependent mechanism to polarize intracellular processes. F-actin flow dynamics have taken center stage over canonical leading-edge F-actin dynamics (treadmilling) in directed cell migration (*51*), where gradients of actin network compression and destruction, regulated by myosin and cofilin, were shown to operate in the cytoplasm. In zebrafish oocytes, directional bulk F-actin dynamics induces phase segregation of ooplasm and yolk granules that in turn induces F-actin comet assembly at the yolk granules, which pushes them into the opposite direction (*52*). Actin clouds, phases, gel condensation and fluidation, and passive and active diffusion have been characterized as biomechanical forces that can combine with branched-chain actin or actomyosin dynamics to confer directionality to organelles (*53*). The Formin2- and MyoVb-dependent dynamics of an F-actin mesh combined with vesicles, for instance, can create a gel-like cytoplasmic pushing mechanism (“active diffusion”) that positions organelles as large as nuclei in mouse oocytes (*54*, *55*) and centers spindles in the mouse zygote to allow for the switch from asymmetric to symmetric cell division (*56*). The biomechanical analysis of distinct dynamic F-actin assemblies (flow, mesh, patches) in polarizing *C. elegans* intestinal cells may reveal yet another mode of actin’s uncanny ability to generate spatial organization and directional movement within a cell.

### Cortical actin dynamics

Our findings suggest that F-actin moves coincidentally through the cytoplasm and along the cortex from the basolateral to the emerging apical domain of polarizing *C. elegans* intestinal cells. Rho-dependent cortical actomyosin dynamics is a key polarity cue in nonepithelial cells, and it also determines polarity in the *C. elegans* one-cell embryo (zygote) (*57*), where it restricts PAR-3, PAR-6, and PKC-3 to the anterior cortex (*58*). Anterior-posterior PAR asymmetries and polarity itself are thought to be first determined at the cortex (*59*, *60*), with cortical F-actin flows able to asymmetrically self-propagate and later self-align along the equatorial cortex during cytokinesis (*59*, *61*). Cortical polarity establishment by membrane domain boundary definition via cross-inhibition of anterior-posterior PARs is a process considered to be principally mirrored by PARs and other apicobasal membrane-based polarity cues in epithelia (*62*, *63*). A cortical (apical) actomyosin network was recently also implicated in the polarization, albeit not in the positioning, of the apical domain in epithelial cells (*64*).

In contrast to the well-characterized process of cortical PAR polarization, it is not clear how PARs are recruited toward the cortex and whether this recruitment itself is polarized. Although not considered drivers of polarity, intracellular processes, such as vesicle- and actin-based dynamics, are known to affect *C. elegans* zygotic polarity: Vesicle asymmetries are present (*65*); Arp2/3-dependent recycling maintains PAR-6 at the anterior cortex (*9*); and Arp2/3 dependent cytoplasmic actin dynamics moves the male pro-nucleus to the anterior domain (*66*). Modeled on the analysis of zygotic PAR polarity, the analysis of *C. elegans* intestinal polarity has remained focused on boundary definitions at the cortex, including the polarized positioning of apical PARs. However, unlike anterior PARs in the zygote (restricted to the anterior from an initial pan-membranous location), apical PARs (and junctions) are directly recruited as foci to the nascent apical domain and its lateral membrane boundaries in the intestinal cells (*15*, *24*, *26*, *67*). It is not clear how lateral PAR foci are consolidated into a contiguous apical PAR lining (and how apical spot junctions are consolidated into lateral junctions). If directly recruited from the cytoplasm, lateral PARs and apical spot junctions might be the results of “imprecise” delivery. On the other hand, F-actin, here observed to shift along the cortex to the nascent apical domain, could also provide the postulated cytoskeletal support for a regulated lateral-to-apical shift of PAR-3, currently considered to initiate polarity in the *C. elegans* intestine (*15*, *24*). A concomitant function of cytoplasmic and cortical F-actin dynamics in apical PAR polarization would provide robustness to membrane polarity establishment in the epithelial tissue context.

### An alternative mode of epithelial membrane polarization

This study, in conjunction with the accompanying article (*13*), proposes a model for apicobasal membrane polarity where the actin-directed, vesicle-based delivery and insertion of the apical domain (here identified by the apical membrane identity marker ERM-1) partitions apicobasal membrane domains and positions apical membrane– and junction-based polarity cues (e.g., PAR-3, PAR-6, and PKC-3; Fig. 10). This proposed model places intracellular polarity cues—bcAMs/actin (this study); multiple components of biosynthetic-secretory vesicles (the accompanying study)—upstream of membrane-based polarity cues within the process of de novo polarized membrane biogenesis. Early-acting intracellular polarity cues, as identified here, raise the possibility that apical PARs, as well as junction components, can be polarized in epithelia by their recruitment from the cytoplasm. PARs might be directly delivered by vesicles or by other modes of polarized transport, or they might be secondarily recruited by components of the newly inserted apical membrane (e.g., by GSLs or their local enzymes) (*7*, *68*), in which case they could travel either through the cytoplasm or along the cortex.

The establishment of epithelial membrane polarity is a multistep process that depends on reinforcement, requiring a succession of molecular cues that position, assemble, and expand the membrane, and build its domain-specific microdomains (*2*). In a model of membrane polarization via apical domain insertion, apical PARs could operate as early reinforcers of apical polarity by acting as platforms for the recruitment of submembranous apical molecules that in turn act as platforms at later stages [e.g., ERMs at the stage of apical microvilli assembly (*69*)]. Apical PARs could also reinforce apical polarity by securing the insertion of an expanding apical domain via their canonical functions in (i) separating the emerging apical from the basolateral domain by apico-lateral junction assembly (*70*), (ii) defining apicobasal domain borders within the signaling network of mutual inhibition of apical and basal polarity cues (*63*), and (iii) tethering the polarized biosynthetic vesicle trajectories to the expanding apical domain (*71*).

A role of vesicular trafficking as intracellular polarity cue is supported by observations in nonepithelial, flat-epithelial, and tubular-epithelial tissues from worms to humans, where various trafficking molecules continue to be identified by their contribution to the positioning of the anterior, apical, or lumenal membrane domain, respectively (*6*–*12*). Perturbations of some of these molecules induce phenotypes closely resembling the here-observed apicobasal polarity conversion phenotype of the *C. elegans* intestine. For instance, a Rab11-dependent transcytotic-recycling defect causes polarity inversion in Madin-Darby canine kidney (MDCK) cysts that mirrors polarity conversion in the *C. elegans* intestine, which can also be induced by depleting RAB-11 (*6*, *8*, *13*). The identification of additional, foremost secretory trafficking components as apical polarity cues in the *C. elegans* intestine could suggest that recycling (MDCK) and secretion (*C. elegans* intestine) converge on an actin-guided anterograde vesicle trajectory that traverses the recycling compartment to position the apical domain [see the accompanying article (*13*)].

The role of actin-guided secretory trafficking as a polarity cue has precedence in single-cell organisms (*62*, *72*). ACT1, the single actin of budding yeast, mediates polarized cell division by directing the secretory pathway to the bud site to insert the nonpolarized membrane for the daughter cell. The formin-dependent actin cables that route secretory vesicles to the bud site can also operate along the yeast cortex (*73*). Actin-guided secretion is likewise required for CDC-42–dependent spontaneous single-cell polarization in yeast (*74*) and for polarity site relocation during yeast mating (*75*). However, unlike the bcAM-powered basolateral-to-apical F-actin shift that appears to drive vesicles toward the apical domain in *C. elegans* intestinal cells, the prior polarization of cortical actin in yeast “pulls” vesicles toward itself on actin cables that were previously radiated into the cytoplasm. In a similar “pulling” movement, the polarized Arp2/3-rich cortical actin cap in mouse oocytes maintains the spindle position by generating an actin flow that results in “retrograde” cytoplasmic streaming toward itself (*76*).

It remains to be determined how the actin-dependent anterograde vesicle trajectory is oriented in epithelial cells and what might trigger its asymmetric orientation. The midbody, a remnant of cell division, serves as landmark for apical transcytosis during polarity inversion in MDCK cysts (*77*). In the *C. elegans* intestine, the midbody, although not a candidate for the cortex-based lateral-to-apical movement of PAR-3 (*15*, *67*), might orient vesicle trajectories directly or indirectly via actin, a process that could predate or accompany the cortical PAR movement (see above, cortical actin dynamics). The midbody moves to and remains at the apical membrane in the early-embryonic *C. elegans* intestine, where RAB-11^+^ vesicles can be detected before PARs are polarized (*78*). Extracellular signals—e.g., basement membrane–derived extracellular matrix (ECM) signals—are conserved triggers for epithelial polarity that can directly or indirectly confer directionality to F-actin dynamics or vesicle trajectories (*79*). In MDCK cells, ECM signals initiate polarity inversion and induce the basolateral endocytosis of future apical membrane components, including ERMs/ezrin (*80*). No ECM-derived polarity signals have yet been identified in the *C. elegans* intestine (*26*, *81*), but multiple basement membrane components have now been shown to be expressed in the early-embryonic intestine (*82*). Lateral cortical polarity complexes, consisting of PAR-3 and the junction component HMR-1/cadherin, were recently suggested as intra-intestinal, albeit extracellular, polarity triggers in the *C. elegans* intestine (*83*). They must, however, like PARs and junctions, themselves be polarized to apical and apico-lateral domains, respectively (discussed above).

The here-proposed alternative mode of membrane polarization may be conserved at the earliest stages of cell polarization in mammals. Polarity in the eight-cell mouse embryo is established by the asymmetric insertion of the apical domain, an event likewise characterized by the apical recruitment of PARs and ERMs/ezrin (*84*). Moreover, the process of apical domain insertion is developmentally regulated by Tfap2c and Tead4, transcription factors that induce the expression of regulators of branched-chain actin dynamics such as Arp2/3 complex components. While Rho-dependent cortical actin dynamics was found to laterally expand the apical domain along the embryo cortex, actin dynamics was also noted to be required for the recruitment of apical membrane components toward the apical domain, a process not yet understood (*85*). It is therefore tempting to speculate that branched-chain actin dynamics also establishes polarity in the mammalian embryo by asymmetrically routing anterograde vesicle trajectories to the nascent apical domain. Recent reports on the polarization of human pluripotent stem cells via intracellular lumenogenesis by the “apicosome” (*86*) could furthermore suggest that the here proposed alternative mode of membrane polarization is conserved from *C. elegans* to humans.

## MATERIALS AND METHODS

### Experimental model

Extended Methods for the in vivo analysis of polarized membrane biogenesis in *C. elegans* tubular epithelial cells are provided in (*87*, *88*).

### *C. elegans* strains, culture conditions, and genetics

Wild-type (N2 Bristol) and mutant *C. elegans* strains were cultured, and genetic crosses were performed using standard methods (*89*). Worms were generally maintained at 20° to 22°C (unless otherwise noted) on Nematode Growth Medium (NGM) plates seeded with *Escherichia coli* OP50 (*90*). The list of strains used in this study is provided in table S2.

### RNA interference

#### General

Methods have been described in our accompanying article (*13*). Briefly, RNAi was carried out by feeding worms *E. coli* HT115 (DE3), producing double-stranded RNA (dsRNA) of the gene of interest, as previously described (*7*, *91*). Here, before seeding worms onto RNAi plates, animals were washed three times with M9 (*89*) and swirled on the plate in drops of carbenicillin solution (500 mg/ml) to avoid any OP50 contamination. For standard RNAi, bacterial feeding clones were inoculated from LB plates into 1 ml of LB liquid medium containing ampicillin (50 μg/ml) and incubated for 8 to 18 hours at 37°C. Cultured RNAi bacteria (200 μl) were seeded onto agar plates supplemented with 2 mM isopropyl-β-d-thiogalactopyranoside (IPTG) and carbenicillin (25 μg/ml). dsRNA was induced at room temperature for at least 6 hours before picking four to six L4 larvae onto each RNAi plate. Most bacterial clones were derived from the Ahringer genome-wide RNAi feeding library (J. Ahringer, Welcome Trust/Cancer Research UK Gurdon Institute, Cambridge, UK). The integrity of all RNAi clones was verified by sequencing.

#### Scaled-intensity RNAi

##### Rationale

Most genes examined in this study have essential, pleiotropic, maternal-effect, and dose-dependent functions in basic cellular processes (e.g., in intracellular trafficking and cellular morphogenesis). Germline mutant or strong tissue-specific (e.g., intestinal) loss-of-function conditions mask these genes’ specific effects on polarized membrane biogenesis by disrupting these basic cellular processes. RNAi was the method of choice for loss-of-function studies of these genes since it can be titrated to produce a range of mild to severe phenotypes. It also effectively targets maternal RNA.

##### Procedures

RNAi conditions were empirically determined for any given gene and experiment by modulating IPTG concentrations in RNAi plates, diluting the RNAi clone of interest with different amounts of mock RNAi bacteria, choice of age of the parental strain in which RNAi was induced (L2 to adult), and using the RNAi-sensitive strain *rrf-3(pk1426)* (*87*). In addition, conditions were varied by temperature (15°C, 22°C, and 25°C) and time of interference, i.e., RNAi was induced either in parents (evaluating the F1 progeny), in larvae (evaluating the same generation; conditional larval RNAi), or in adults. Conditional larval RNAi was carried out by bleaching 30 to 50 gravid adults in one drop bleaching solution (a 1:4 mix of 10 M NaOH and household sodium hypochlorite) on the edge of an RNAi plate and allowing hatched larvae to crawl to the bacterial lawn (*87*). Conditional RNAi was also induced at other stages of development by transferring L1, L2, L3, and L4 larvae or adult animals to RNAi plates and scoring the same generation. Appropriate controls were added to ensure that RNAi was effective when induced at later time points during development (e.g., by assessing the ability of *gfp* RNAi to remove fluorescence in a GFP-expressing strain). All experiments were repeated three or more times for each dataset. See table S3 for specific conditions used in each experiment.

#### Analysis of actin isoforms by 3′UTR RNAi

##### Rationale

The genetic analysis of actin has been complicated by actin’s essential and pleiotropic functions, the sensitivity of filament assembly to any changes in actin stoichiometries, and the large families of almost identical and often redundant actin isoforms. Loss-of-function analyses of actin isoforms typically fail to produce effects if targeting single isoforms. On the other hand, loss-of-function strategies that remove several or all actins induce sterility/early lethality when used globally or severe cellular morphogenesis defects when used in a tissue-specific manner. The mutational analysis of actins has largely recovered dominant alleles that typically induce dominant negative, but also neomorphic, changes [e.g., a dominant *act-2* germline mutation, but not an *act-2* germline deletion, produces embryonic morphogenesis defects in *C. elegans* (*92*)]. We trialed a 3′UTR-directed RNAi approach to (i) test the loss of function of various single and multiple combinations of isoforms without disrupting basic cellular functions and (ii) separately target almost identical isoforms by RNAi (*act-1*, *act-2*, *act-3*, *act-4*, and *act-5* transcripts are closely similar, while 3′UTRs diverge; fig. S6A).

##### Procedures

The 3′UTR of each isoform was targeted starting from the stop codon (fig. S6A). The Clone Mapper tool excluded any possible “off-target” regions (*93*). The desired (specific) 3′UTR region was generated by polymerase chain reaction (PCR) and inserted (Gibson method) (*94*) into the multiple cloning site of the L4440 backbone (a modified version of the pBlueScript plasmid) and sequence-validated. The plasmid was transfected into the *E. coli* strain HT115 (DE3), which carries a defective ribonuclease (RNase) III and an IPTG-inducible T7 polymerase gene, to generate dsRNA (*91*).

##### *Act-5-LE17bp *3′UTR* RNAi (LE17bp: last exon plus 17 bp)*

*act-5-LE17bp *3′UTR RNAi includes a small stretch of the *act-5* exonic sequence beyond the 3′UTR and targets *act-5* more effectively than *act-5 *3′UTR RNAi while also mildly affecting other actin isoforms (fig. S2B and legend). It potently induces intestinal polarity conversion and was used to assess the subcellular localization of ACT-5::GFP::*unc-54-*3′UTR (missing the *act-5* 3′UTR; Fig. 8, *a*ct-5p::**ACT-5::GFP*::unc-54-3’*UTR panels, and fig. S8, M to O).

##### Double, triple, or multiplex RNAi

RNAi bacteria were prepared by mixing the proportional liquid volumes of each desired combination (e.g., 1:1 volumes, or 50% each) before seeding onto agar plates supplemented with IPTG and carbenicillin. All experiments included appropriate controls (corresponding dilutions with mock RNAi bacteria) for combinatorial RNAis. The ability of quadruple *act-1 + act-2 + act-3 + act-4* 3′UTR RNAi (1:4 dilution of each isoform) to induce lethality and morphogenesis defects serves as internal control for *act-1*, *act-2*, *act-3*, and *act-4* single (each isoform is undiluted), double, and triple 3′UTR RNAi (1:2 and 1:3 dilutions), none of which induce obvious phenotypes.

#### Genetic interactions (double mutant/RNAi analysis)

##### Rationale

To bypass the inability to capture convergent genetic interactions between maternal-effect early lethal genes via full null alleles, we assessed the ability of mild double mutant/RNAi conditions to enhance a minimal phenotype or to generate a synthetic phenotype. Using the scaled-intensity RNAi approach, conditions were empirically determined for each gene that induced no phenotype on their own (or that only generate a minimal phenotype) and combined with temperature-sensitive or balanced mutant alleles that appeared wild-type in the presence of a balancer or at the permissive temperature.

##### Procedures

For detailed procedures, please refer to the correspong sections, “*C. elegans* strains, culture conditions, and genetics” and “RNA interference.”

### In vivo analysis of polarized membrane biogenesis in single cells

The expanding, single-layered postmitotic intestine of the late *C. elegans* embryo or early (L1) larva was used as an in vivo model for the analysis of de novo polarized (apical) membrane biogenesis. It allows for the separation of polarized membrane biogenesis from polarized cell division and migration that occur concomitantly during polarized tissue morphogenesis [see our accompanying article (*13*); see (fig. S3) for net apical membrane expansion in the *C. elegans* embryonic and larval intestine]. To distinguish membrane biogenesis defects from sequelae of preceding tissue morphogenesis defects, perturbations were introduced after completion of intestinal morphogenesis (e.g., by conditional larval RNAi). To distinguish defects in polarized membrane addition from defects in polarized membrane maintenance, the effects of perturbations introduced in the expanding larval intestine were compared to the effects of perturbations introduced in the no-longer expanding adult intestine. See above, RNAi, for procedures.

### Measurement of apical membrane expansion in the developing intestine

Using confocal images, the length of the apical (lumenal) membrane of the intestine was measured at different stages of development, from the embryo to the adult, with the help of the “Annotation and Measurement tool: polyline length tool” provided in the NIS-Elements of the confocal microscope. The results were computed by a simple statistics table and exported into an Excel sheet. Triplicates of 15 animals each were examined. Image pixels: 512 × 512. Objectives used: for embryo and L1: 60×; for L2 to L3 larvae: 20×; for L4 larvae and adults: 10×.

### DsRed feeding

Methods have been described in our accompanying article (*13*). DsRed HT115 RNAi bacteria were generated with a DsRed-expressing plasmid. Animals were fed on plates containing RNAi bacteria targeting the gene of interest and control RNAi bacteria for 2 days. More than 50 animals were transferred to plates containing a 1:1 mixture of gene-specific and DsRed-containing RNAi bacteria at least 15 hours before evaluation.

### Temperature shift experiments

Experiments were performed in a temperature-controlled incubator or temperature-controlled room. Same stage animals were evaluated at different time points at 15°C versus 22°C to account for slower development at the lower temperature.

### Fluorescent fusion proteins

All strains with fluorescently labeled fusion proteins are described in table S2. The subcellular localization of most fusion proteins used in this study to identify polarized membrane domains and junctions was previously confirmed by us and others by various labeling procedures (e.g., antibody staining, chemical staining, germline knock-ins). Many were also internally controlled by a panel of different transgenes, fluorophores, and by both N- and C-terminal fusions. Although not critical for their use as markers, most were also shown to be functional by the rescue of the corresponding mutant phenotype (see text and figure legends for references).

#### Rationale for generating exogenously tagged fusion proteins and extrachromosomal transgenes

The bcAMs UNC-60, ARX-2, CAP-1, and the five actin isoforms were exogenously tagged to (i) avoid the risk of generating phenotypic effects by modifying these molecules’ germline loci, given the sensitivity of actin filament assembly to minor changes in actin modulation and stoichiometries; (ii) support a high-resolution subcellular analysis of these ubiquitously expressed molecules by restricting their expression to the intestine (actin isoforms were expressed from their own promoters to assess whether they were expressed in the intestine); and (iii) achieve optimal expression levels by modulating transgene copy number (see procedures below). All bcAMs were previously shown to be expressed in the intestine (references in text). They were used in this study to determine subcellular localization changes during development. Fig. 4 and fig. S4C[Fig F6] document identical expression of exogenously and endogenously tagged fusions for ARX-2::GFP and ARX-2::RFP and demonstrate the superior resolution of subcellular structures labeled by the exogenously tagged ARX-2::GFP.

#### Procedures

Methods were previously described (*87*, *88*).

##### Promoters

To examine expression from an early time of intestinal development, most fusion proteins were directed to the intestine by the *elt-2* promoter, exclusively expressed in the clonal intestinal *E*-lineage (*95*–*97*). To optimize expression and facilitate cloning procedures, the length of this promoter was empirically determined for each construct and varied over a range of 600 to 5000 bp (table S2).

##### Cloning

Genes were tagged at their 3′ or 5′ ends and cloned in frame with GFP, tagRFP, or other fluorophores, using standard cloning procedures or Gibson Assembly (*94*). We generated all bcAMs (UNC-60, ARX-2, CAP-1) and several vesicle-associated markers (e.g., RAB-11) as N-terminal and C-terminal fluorescent fusions to confirm subcellular localization. Promoters (either *elt-2p* or the endogenous promoter) were amplified by PCR from wild-type genomic DNA. Some cDNAs were amplified from their respective cDNA plasmid clones (gifts from Y. Kohara, National Institute of Genetics, Mishima, Japan). GFP and tagRFP DNA fragments were amplified from ppD95.75 and pPD284, respectively [(*98*); Addgene]. For 3′UTRs, *unc-54, elt-2*, or the endogenous 3′UTR of the gene were used, as indicated. Plasmids were either cloned by standard procedures or generated using the PCR-stitching method (*99*). All recombinant plasmids and PCR-stitched chimeric DNAs were sequence-verified.

##### Transgenesis

To optimize expression levels, we generated extrachromosomal transgenes by germline transformation using standard procedures (*100*), as it allowed for testing a range of different DNA concentrations. With the goal to use the lowest concentration possible to achieve good subcellular expression, a concentration of 1 to 2 ng/μl DNA was used for germline transformation for most genes, with the expectation to generate low transgene copy numbers (*101*, *102*). All strains were examined for the absence of overexpression-induced artifacts. To generate transgenic lines with extrachromosomal arrays, DNAs were microinjected into either wild-type, *unc-119*, or other mutant *C. elegans* gonads for germline transformation, with or without the pRF4 plasmid that encodes a mutant collagen [*rol-6(su1006)*] marker, and/or the *unc-119^+^* rescuing construct, using standard techniques (*100*, *101*). Multiple lines were generated for each transgene. Primers for cloning are available on request. See table S2 for strain genotypes.

#### Specific fusion proteins

##### ERM-1::GFP

ERM-1 is used in this study as an apical membrane identity marker [see our accompanying article (*13*)]. To avoid any possible disturbance of the *erm-1* germline locus that might interfere with apical membrane biogenesis, an integrated transgenic line is used in this study (VJ610, table S2). This strain was previously characterized, is devoid of overexpression artifacts, and accurately tracks ERM-1’s subcellular localization, confirmed by a spectrum of different transgenic strains (high and low copy number, fused to different fluorophores), labeling approaches, rescue of the *erm-1* mutant phenotype, and an ERM-1::GFP germline knock-in (*7*, *10*, *103*, *104*).

##### GFP, mCherry, or tagRFP fusions with components of endomembranes or vesicle membranes and coats

GFP, mCherry, or tagRFP fusions with components of endomembranes or vesicle membranes and coats are directed to the intestine either by the *elt-2* (this study) or *vha-6* promoter (the latter strains are gifts from B. Grant; table S2).

##### PAR-3::mCherry, PAR-6::GFP, and GFP::PKC-3

PAR-3::mCherry, PAR-6::GFP, and GFP::PKC-3 are CRISPR knock-ins (gift from K. Kemphues; table S2). Two different ACT-3::GFP strains were used in this study: one carries a translational GFP fusion, and the other a transcriptional fusion (Fig. 7; see table S2).

##### ACT-3::GFP

The translational ACT-3::GFP fusion was not compatible with survival beyond the embryonic stage, although robustly expressed in a variety of different lines, even when the plasmid was introduced at low concentration (1 ng/μl).

##### LifeACT::GFP

To minimize the risk of interference with actin filament assembly, the exogenous LifeACT was chosen over endogenous small F-actin–binding molecules such as the C-terminal actin binding sites of *C. elegans* ERM-1 or of the closely related *Drosophila* MOE (*9*, *105*). LifeACT strains were backcrossed four times and integrated using ultraviolet (UV) irradiation to establish nonmosaic transgenic lines (*106*).

##### Photo-convertible Dendra2 and photoactivatable PA-GFP fusions

A pFG102 vector was constructed, containing 566 bp of *elt-2* promoter followed by LifeACT and Dendra2 or PA-GFP, the *elt-2* 3′UTR, and a kanamycin selection marker.

##### Dendra2

Dendra2 was PCR extracted from pEG412 (Addgene) and sequence-optimized for worm expression (three synthetic introns were inserted) (*107*), gel-extracted, type IIM restriction enzyme (DPNI)-treated, and used to replace the GFP in pFG102, using Gibson cloning.

##### PA-GFP

Initially described by Patterson and Lippincott-Schwartz (*108*), PA-GFP was previously used by Mijalkovic *et al*. (*109*) in *C. elegans*. We engineered a photoactivatable GFP using Genewiz DNA block and Gibson cloning to introduce all four known PA-GFP mutations (L64F, T65S, V163A, and T203H) into the Fire vector pPD95.75 GFP. The LifeACT coding sequence (encoding only 17 amino acids) is separated by a flexible linker (encoding seven inert amino acids) from the protein, and synthetic introns are optimized for brighter expression in *C. elegans* (Fig. 9D). Constructs were microinjected, and transgenic strains were generated, as described above.

### Immunohistochemistry

Methods have been previously described (*87*, *88*). Briefly, L1 larvae were collected in M9 medium (*89*) onto slides coated with 1 to 2% poly-l-lysine (Sigma, P5899), covered with overhanging coverslips, and then permeabilized by flash freezing in liquid nitrogen and subsequent flicking off of the coverslip. Fixation was performed by sequential incubation in methanol and acetone at −20°C. Immunofluorescent staining was carried out as described [procedures are demonstrated in (*87*)]. For MH33 staining, slides were exposed to the first antibody (1:10 dilution) overnight at 4°C, washed, and then exposed to the secondary antibody for 1 hour at room temperature. Permount (Fisher, SP15-100) was used as a coverslide mounting medium.

### Confocal and dissecting microscopy

Methods are described in our accompanying article (*13*). Briefly, differential interference contrast (Nomarski) and confocal images were acquired using a Nikon Eclipse-Ti inverted microscope equipped with a C2 confocal system. Most confocal images were obtained with a 63× objective. Exposure to fluorescent light was minimized to avoid bleaching, and images were obtained within minutes of mounting. Images were captured as single sections or a series of sections along the *z* axis with differing thicknesses (generally 0.1 to 1.0 μM). For multichannel images, individual channel intensity was adjusted and the samples were scanned sequentially to exclude the possibility of bleed-through between channels. Confocal imaging parameters, such as pinhole size and laser intensity, were empirically determined based on fluorophore intensity and experimental setting (avoiding phototoxicity, photobleaching, bleed-through). Deconvolution software was only used in Fig. 8, where indicated, and images were not further edited except for adjustment of brightness and contrast (Adobe Photoshop).

### The eyedropper tool

To measure the intensity of the merged channels of two fluorophores, we took advantage of the eyedropper tool in Adobe Photoshop 2022. The eyedropper tool provides color information and displays the intensity value of each RGB (red, green, blue) band of any single pixel under the pointer. In addition, it displays the measured color of that particular pixel in a large circle that is normally indistinct to the eye. The values in the info panel are the original color values without any adjustment. The eyedropper tool’s instruction is provided on the Adobe website as Histogram and Pixel Value.

### Fluorescence intensity measurement during time-lapse imaging of activated GFP

Fluorescence intensity of the entire image was measured using ImageJ (*110*) software. Since all images recording photoactivated PA-GFP during one time-lapse imaging experiment were taken with the identical confocal image acquisition criteria and settings (e.g., laser intensity, gain, pixels, and pinhole), background noise subtractions were not performed. Results were reported as integrated density. Triplicates of 15 animals were analyzed for each set.

### Transmission electron microscopy (TEM)

Methods have been previously described (*13*). Briefly, larvae were washed off in standard M9 medium (*89*) and collected into 1.5-ml Eppendorf tubes. They were then fixed in 2.5% glutaraldehyde and 1.0% paraformaldehyde in 0.05 M sodium cacodylate buffer (pH 7.4) plus 3.0% sucrose. Before fixation, the cuticles were “nicked” with a razor blade in a drop of fixative under a dissecting microscope to allow the fixative to penetrate. After an initial 2-hour fixation at room temperature, the specimens were transferred into fresh fixative and stored overnight at 4°C. Specimens were rinsed several times in 0.1 M cacodylate buffer and then postfixed in 1.0% osmium tetroxide in 0.1 M cacodylate buffer for 2 hours on ice. After postfixation, specimens were rinsed several times in 0.1 M cacodylate buffer and then embedded in 2.0% agarose in phosphate-buffered saline (PBS) for ease of handling. The agarose blocks were dehydrated through a graded series of ethanol to 100%, dehydrated briefly in 100% propylene oxide, and pre-infiltrated overnight on a rocker in a 1:1 mixture of propylene oxide:Eponate resin (Ted Pella, Redding, CA). The following day, the agarose blocks were infiltrated in 100% Eponate resin for several hours, then embedded in flat molds in fresh Eponate resin, and allowed to polymerize for a minimum of 24 hours at 60°C. Thin sections were cut on a Leica UC7 ultramicrotome and collected on formvar-coated grids, poststained with uranyl acetate and Reynold’s lead citrate, and viewed in a JEOL 1011 TEM at 80 kV equipped with an AMT digital imaging system (Advanced Microscopy Techniques, Danvers, MA).

### Stochastic optical reconstruction microscopy

#### Mounting

Coverslips, not glass slides (length, 50 mm; thickness, 0.16 to 0.19 mm; Fisherbrand, 12-544-EP), were used to mount the worms. Thirty microliters of 2% poly-l-lysine (Sigma, p5899) was added per two coverslips, sandwiched, separated, and air-dried for 30 min. Five microliters to 10 μl of 1× PBS was added, and 50 to 100 animals were mounted onto the slide (in 1× PBS). The second coverslip (length, 22 mm; thickness, 0.13 to 0.17 mm; Fisherbrand, 12-542-BP) was added on top, and with gentle pressure, all animals were flattened. The sandwich was placed on a −80°C metal block (using liquid nitrogen) and frozen for 5 min. Once frozen, one coverslip was flicked off.

#### Fixation and permeabilization

All procedures were carried out on an iced metal block. Five microliters of 3% glutaraldehyde (Ricca, R3281800) and 1.75 μl of 10% Triton X-100 in 43.75 μl of CS buffer [10 mM MES (pH 6.1), 150 mM NaCl, 5 mM EGTA, 5 mM glucose, 5 mM MgCl_2_] were applied for 2 min, followed by 10 min of 30 μl of 3% glutaraldehyde and 15 μl of CS buffer, followed by 7-min incubation in freshly made 0.1% NaBH_4_ (Sigma, 213462) in 1× PBS. Samples were washed three times using 1× PBS.

#### Staining

The desired antibody was applied overnight at 4°C or for 1 to 2 hours at room temperature. The following chemicals and antibodies were used: Alexa Flour 647–conjugated phalloidin (Fisherbrand, A22287) 9:100 in 1× PBS; Cy3B and Dy750-conjugated anti-GFP nanobodies, 1:100 in blocking solution; IFB-2 primary mouse antibody MH33 1:10 and secondary Dy750-conjugated donkey anti-mouse antibody. Phalloidin was used to detect all actins and to optimize resolution (incorporation into actin filaments is superior compared to actin fluorophore fusions) (*111*). Staining as above (immunohistochemistry).

#### Imaging

Animals were imaged in PBS buffer containing 100 mM cysteamine (Sigma), 5% glucose (Sigma), glucose oxidase (0.8 mg/ml) (Sigma), and catalase (40 μg/ml) (Roche Applied Science).

### In vivo time-lapse imaging of photoconverted and photoactivated GFP

#### Photo-conversion (Dendra2)

##### Mounting

Early bean, bean, comma, or 1.5-fold stage embryos were selected and transferred onto agarose pads. A square-shaped 3 mm × 3 mm agar pad (5% noble agar) was created with enough O_2_ ventilation to avoid hypoxia. Four to five transgenic embryos were mounted onto this pad into a 1-μl drop of M9 and covered with a standard glass coverslip.

##### Photo-conversion

Photo-conversion was carried out using a laser scanning confocal microscope equipped with a 63× objective. Before conversion, a z-stack image was taken using fluorescein isothiocyanate (FITC) and tetramethylrhodamine isothiocyanate (TRITC) channels and customizing parameters to avoid bleed-through between the channels (TRITC laser wavelength: 488.0, Photomultipliers High Voltage (PMT HV): 70, PMT offset: 0, and FITC laser wavelength: 561.0, 6.0, PMT HV: 78, PMT offset: −15, and the Transmission Detector (TD) light channel PMT HV: 89, PMT offset: 0). The region of interest (ROI) was selected using the ROI tab, and stimulation was applied (UV 405 nm, C2plus stimulation as 0.36; Near Diffraction (ND) limited stimulation: 1 s, 3 loop). Another postconversion z-stack image was taken at minute 0, followed by images at desired time intervals during further embryonic and larval development.

#### Photo-activation (PA-GFP)

Equipment and mounting were as above (photoconversion). Before photoactivation, an image of the worm was acquired as described above for photoconversion. Next, using a 405-nm UV laser, the ROI was photoactivated using the customizable ROI bleaching tool of the laser scanning confocal acquisition software. Here, optimal photoactivation was achieved using the 405-nm laser at 10% power. Worms were imaged immediately following photoactivation. The image acquisition settings (e.g., exposure, gain, laser power, and binning) were strictly maintained between intervals in time-lapse imaging experiments.

Images were not further edited to remove movement artifacts (background of activated GFP or converted Dendra2 outside the targeted area) induced by rapid embryo movement within the eggshell during later stages of embryonic development.

### Statistics

Statistical analyses were performed by GraphPad Prism 9.3.0 (Mac) software. All values are mean ± SEM of three or more independent experimental data sets. *P* values were calculated by analysis of variance (ANOVA) or Student’s *t* test, as indicated in the legend of each figure. *n* (sample size) is indicated in the text and in the figure legends.

### Software

Clone Mapper (*93*), Transfac T-coffee (*112*), ImageJ (*110*), Clustal Omega (*113*) were used.

## Supplementary Materials

### This PDF file includes:

Figs. S1 to S9 Legends for tables S1 to S3 Legends for movies S1 and S2 References

## Notes

View/request a protocol for this paper from *Bio-protocol*.
