# Supplementary material for: Branched-chain actin dynamics polarizes vesicle trajectories and partitions apicobasal epithelial membrane domains
Source: Sci Adv. 2023 Jun 28;9(26):eade4022. doi: 10.1126/sciadv.ade4022 (PMC10306301; doi:10.1126/sciadv.ade4022)
Supplement: Supplementary file 1 — Figs. S1 to S9 Legends for tables S1 to S3 Legends for movies S1 and S2 References [file sciadv.ade4022_sm.pdf]

Science Advances
AAAS

Supplementary Materials for

Branched-chain actin dynamics polarizes vesicle trajectories and partitions apicobasal epithelial membrane domains

Gholamali Jafari *et al.*

Corresponding author: Verena Gobel, vgobel@mgh.harvard.edu; Gholamali Jafari, gjafari@mgh.harvard.edu

Sci. Adv. **9**, eade4022 (2023)
DOI: 10.1126/sciadv.ade4022

The PDF file includes:

Figs. S1 to S9
Legends for tables S1 to S3
Legends for movies S1 and S2
References

Other Supplementary Material for this manuscript includes the following:

Tables S1 to S3
Movies S1 and S2

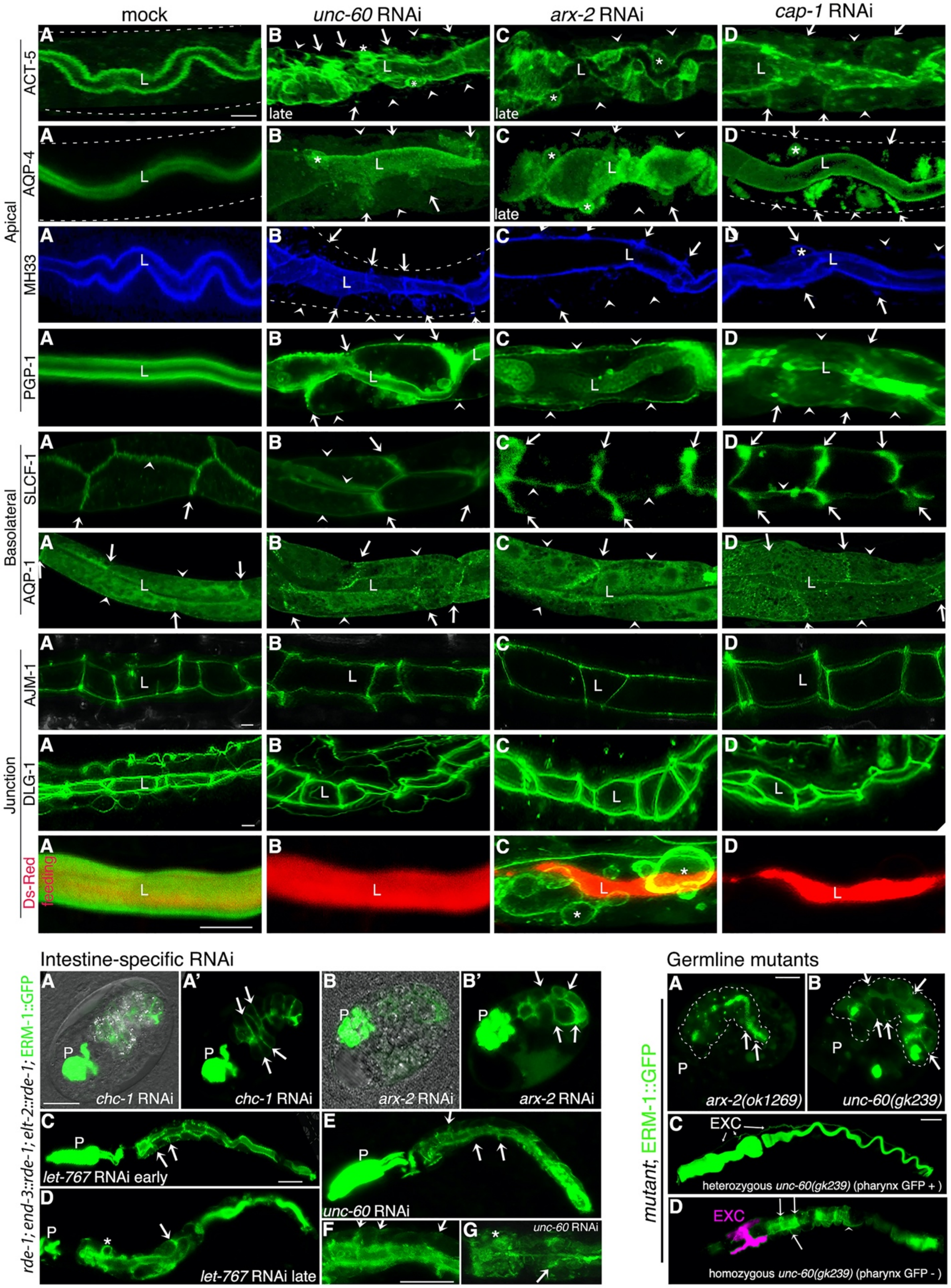

mock
unc-60 RNAi
arx-2 RNAi
cap-1 RNAi
Apical
ACT-5
AQP-4
MH33
PGP-1
Basolateral
SLCF-1
AQP-1
Junction
AJM-1
DLG-1
Ds-Red feeding
late
Intestine-specific RNAi
rde-1; end-3::rde-1; elt-2::rde-1; ERM-1::GFP
chc-1 RNAi
arx-2 RNAi
let-767 RNAi early
let-767 RNAi late
unc-60 RNAi
Germline mutants
mutant; ERM-1::GFP
arx-2(ok1269)
unc-60(gk239)
EXC
heterozygous unc-60(gk239) (pharynx GFP +)
homozygous unc-60(gk239) (pharynx GFP -)

Figure S1. The branched-chain actin modulators (bcAMs) UNC-60, ARX-2, and CAP-1 determine the polarized positions of apical, but not basolateral membrane components on expanding membranes of L1-larval intestinal cells. Intestine-specific RNAi and germline mutants (related to Figure 1).

Analysis of apical, basolateral and junctional membrane components. Confocal sections of two pairs of cells surrounding the lumen of L1-larval intestines at early stages of polarity conversion are shown (otherwise marked as 'late'). L (Lumen) = apical membrane domain. Arrows point to some of the lateral portions, arrowheads to some of the basal portions of basolateral membranes, and asterisks indicate ectopic lumens. For clarity, the outline of the intestine is traced by dashed lines in some images (note that the intestine is single-layered: its outline runs alongside the basal portions of basolateral membranes of intestinal cells). A scaled-intensity RNAi approach was used throughout (Materials and Methods). All membrane-/junction markers are translational GFP fusions (table S2), except for IFB-2, immunostained with MH33/Cy5.

Apical membrane panel: *unc-60* (**B**), *arx-2* (**C**), *cap-1* (**D**) RNAi misposition apical submembranous (ACT-5/actin; MH33/IFB-2/intermediate filament) and integral membrane components (AQP-4/aquaporin; PGP-1/P-glycoprotein-related) to basolateral membrane domains and ectopic lumens (**A**: wild-type localization). Ectopic lumens (ELs, asterisks) develop on prior basolateral membrane domains at later stages of polarity conversion ('late'). Apical membrane components are also displaced to the cytoplasm, to cytoplasmic patches, vacuoles and to apical and basolateral membrane blebs. Some of these changes may be caused by conserved structural functions of bcAMs and actin in endo- and plasma membrane modeling (see Figure S3 for glossary of terms for different membrane biogenesis defects).

Basolateral membrane panel: *unc-60* (**B**), *arx-2* (**C**), *cap-1* (**D**) RNAi have no detectable effect on the polarized positions of the integral basolateral membrane components SLCF-1/solute-carrier-family-member and AQP-1/aquaporin (**A**: wild-type localization). The recruitment of SLCF-1 and AQP-1 to the basolateral membrane (arrows) is, however, affected. Note that UNC-60A functions in basolateral recycling in the mature *C. elegans* intestine (*117*). Images for SLCF-1 are focused on the basal portion of the basolateral membrane (arrowheads), not the apical membrane domain/lumen (not visible in these images).

Apical junction panel: *unc-60* (**B**), *arx-2* (**C**), *cap-1* (**D**) RNAi maintain the peri-lumenal ladder pattern of apical junctions during polarity conversion (AJM-1 and DLG-1/discs-large of the junctional DAC complex (*118*) are shown; **A**: wild-type localization; note that a scaled-intensity RNAi approach is used). Junction integrity is demonstrated by the failure of ingested DsRed bacteria to leak in-between cells or into basolateral ectopic lumens (asterisks). Excess junctions form at later stages of polarity conversion around ectopic basolateral lumens (compare Figure 1 X to Z; early polarity conversion is shown here). Note that AJM-1 extends into the lateral membrane in *unc-60(RNAi)* intestines. All three bcAMs and actin have previously described structural functions in apical and basolateral membrane and junction biogenesis, with some of these functions being dependent on vesicular trafficking (*21-23, 117, 119*).

Intestine-specific RNAi: bcAMs, like vesicle-based polarity cues, function cell-autonomously in intestinal polarity. *arx-2(RNAi)* embryonic (**B** to **B'**), and *unc-60(RNAi)* larval (**E** to **G**), intestinal polarity defects phenocopy the *chc-1(RNAi)* embryonic (**A** to **A'**), and the *let-767(RNAi)* larval (**C** to **D**), polarity defects, respectively (*chc-1* encodes the clathrin heavy chain and *let-767* a glycosphingolipid-biosynthetic enzyme; both function as vesicle-based polarity cues in the *C. elegans* intestine (*7, 10*)). UNC-60's cell-autonomous function in the intestine implies that this function is mediated by the intestine-specific isoform UNC-60A (*14*). The intestine-

specific RNAi strain (*rde-1; end-3::rde-1; elt-2::rde-1*) is marked with a MYO-2::GFP+ pharynx (P: _P_harynx; table S2).

Confocal projections and Nomarski/confocal overlay images of embryos (A to B'), whole L1-larvae (C to E), and portions of L1-larval intestines (F and G) are shown. Some of the basolateral membranes are indicated by arrows. See Figure 1 D, G, K, and O, S for wild-type larval and embryonic intestinal polarity (apical ERM-1 location), respectively.

Germline mutants. (**A**) Embryonic polarity defect in *arx-2(ok1269*). Note that ERM-1 is entirely displaced from the apical membrane in the anterior intestine in (A). See Figure 1 O and S for apical localization of ERM-1 in wild-type embryos. (**B**) Embryonic and (**D**) L1-larval polarity defects in *unc-60(gk239)*. Wild-type and heterozygotes animals are distinguished from the homozygous mutant by a MYO-2::GFP-positive pharynx (P) (**C**). Excretory canal (EXC) in (D) is pseudo-colored (purple) to distinguish it from the intestine.

Scale bars: 10 µm (indicated in the far-left panel for the corresponding row or set).

See Figure S2D for genotypes of the balanced mutant strains and Figure S3 for anatomy and morphogenesis of the embryonic and larval *C. elegans* intestine.

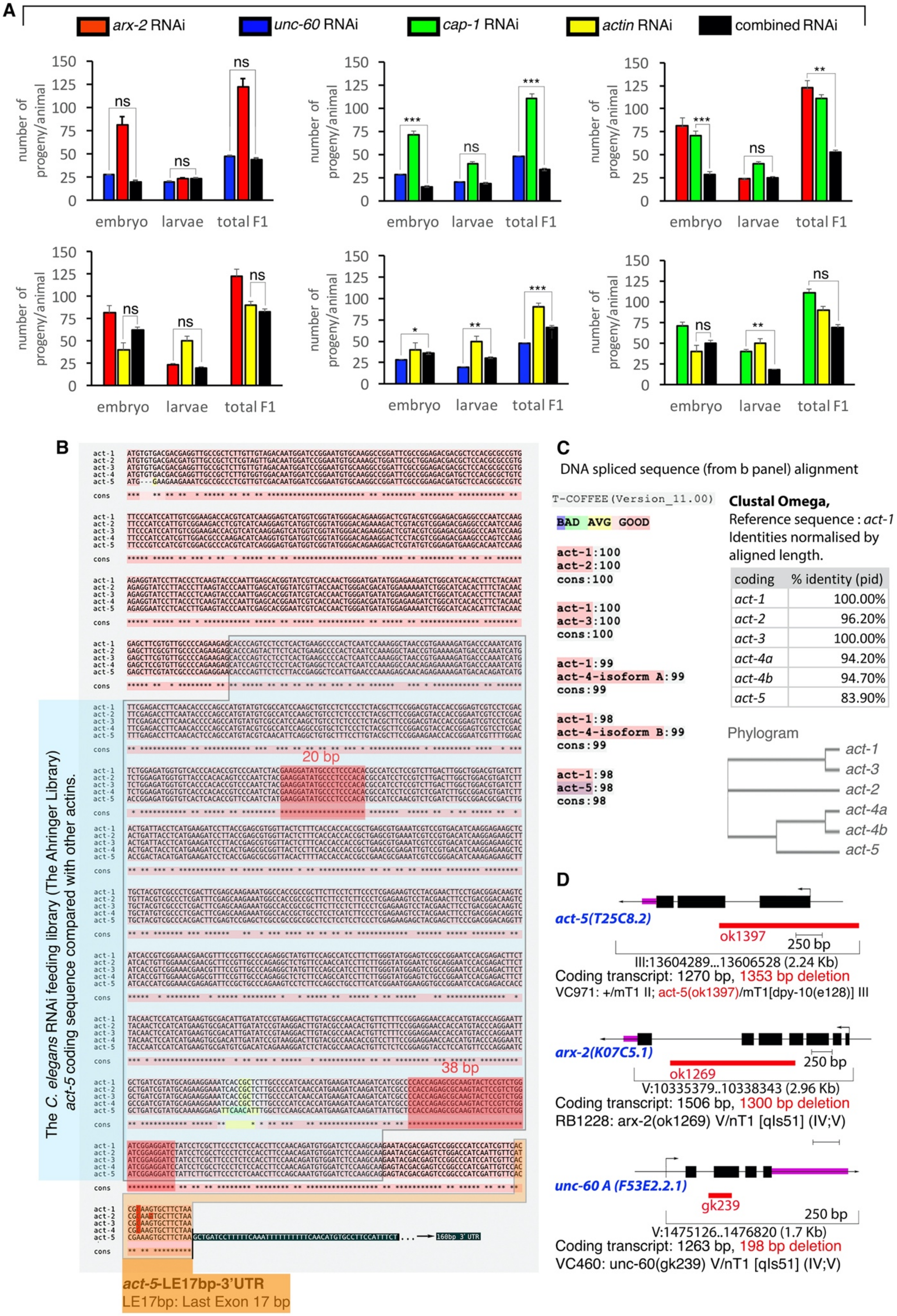
A
arx-2 RNAi
unc-60 RNAi
cap-1 RNAi
actin RNAi
combined RNAi
number of progeny/animal
0
25
50
75
100
125
150
embryo
larvae
total F1
ns
***
**
*
B
The C. elegans RNAi feeding library (The Ahringer Library) act-5 coding sequence compared with other actins.
act-1
act-2
act-3
act-4
act-5
cons
20 bp
38 bp
160bp 3' UTR
act-5-LE17bp-3'UTR
LE17bp: Last Exon 17 bp
C
DNA spliced sequence (from b panel) alignment
T-COFFEE (Version_11.00)
BAD AVG GOOD
act-1:100
act-2:100
cons:100
act-1:100
act-3:100
cons:100
act-1:99
act-4-isoform A:99
cons:99
act-1:98
act-4-isoform B:99
cons:99
act-1:98
act-5:98
cons:98
Clustal Omega,
Reference sequence : act-1
Identities normalised by aligned length.
coding | % identity (pid)
act-1 | 100.00%
act-2 | 96.20%
act-3 | 100.00%
act-4a | 94.20%
act-4b | 94.70%
act-5 | 83.90%
Phylogram
act-1
act-3
act-2
act-4a
act-4b
act-5
D
act-5(T25C8.2)
ok1397
250 bp
III:13604289...13606528 (2.24 Kb)
Coding transcript: 1270 bp, 1353 bp deletion
VC971: +/mT1 II; act-5(ok1397)/mT1[dpy-10(e128)] III
arx-2(K07C5.1)
ok1269
250 bp
V:10335379..10338343 (2.96 Kb)
Coding transcript: 1506 bp, 1300 bp deletion
RB1228: arx-2(ok1269) V/nT1 [qIs51] (IV;V)
unc-60 A (F53E2.2.1)
gk239
250 bp
V:1475126..1476820 (1.7 Kb)
Coding transcript: 1263 bp, 198 bp deletion
VC460: unc-60(gk239) V/nT1 [qIs51] (IV;V)

Figure S2. Genetic interactions in lethality between branched-chain actin modulators (bcAMs) and between bcAMs and actin. Comparison of exonic sequences of *act-1, -2, -3, -4, -5* and areas targeted by a commonly used *act-5* dsRNA. Genomic organization of *act-5*, *arx-2* and *unc-60*, germline deletions and genotypes of germline mutant strains (related to Figure 2).

(**A**) RNAi with each bcAM and actin causes sterility, embryonic lethality, and early larval lethality. Note that the total number of progeny is shown, the majority of which will die as embryos or larvae (wild-type number of progeny is N=220 at 20^0C (*89, 120*)). Compare Figure S2A to Figure 2, R to W (same color coding): genetic interactions among bcAMs in intestinal polarity do not simply reflect their genetic interactions in lethality. bcAMs' interactions in lethality are, however, also compatible with bcAMs' differential effects on actin filament assembly/disassembly (Figure 2, P and Q). Upper row panels: *unc-60*-dependent lethality is not significantly suppressed by *arx-2* RNAi, but is enhanced by *cap-1* RNAi (corresponding to a lower number of progeny), which also enhances the *arx-2*-dependent lethality (as well as the *arx-2*-dependent intestinal polarity conversion; Figure 2, R to T). Lower row panels: *arx-2* and *cap-1* RNAi fail to enhance the actin-dependent lethality (as well as intestinal polarity conversion; Figure 2, U and W). Actin loss suppresses the lethality of *unc-60* RNAi (corresponding to a higher number of progeny), consistent with the alleviation of excess filament polymerization in the absence of actin depolymerization (*unc-60* loss) that may be the dominant effect on the organismal level. >3 replicas were analyzed for each set of experiments; one-way ANOVA was used to determine significance; ***$p < 0.01$

(**B**) Spliced DNA sequence alignment by T-COFFEE reveals almost full identity between *act-1, act-2, act-3, act-4* and *act-5* (yellow = relevant discrepancies; see C for color-coding: bad-average-good). The area targeted by the broadly used *C. elegans* bacterial RNAi feeding clone *act-5* (derived from The Ahringer Library) (*121*)) is indicated by a light-blue overlay. The dark pink-red overlay shows contiguous stretches of identical bases among all five actin isoforms that are longer than 18-22 base pairs, considered necessary for effective RNAi in *C. elegans*. The area targeted by the *act-5-LE17bp-3'UTR* RNAi clone is indicated by a light-brown overlay (discrepant bases highlighted in red). The *act-5-LE17bp-3'UTR* dsRNA targets additional 17 base pairs of the last exonic sequence, of which 14-16 base pairs match *act-1, -2, -3, -4*. *act-5-LE17bp* 3'UTR RNAi and *actin* RNAi (the Ahringer RNAi library clone *act-5*), but not *act-5* 3'UTR RNAi, reduce ACT-1::GFP and ACT-2::GFP expression, consistent with cross-targeting (see Figures 7 and S5 and S8 for comparative analysis of actin isoforms).

(**C**) Comparison of *act-1, -2, -3, -4, -5* exonic sequence conservation scored by T-COFFEE and Clustal Omega (the latter scoring full identities) (*113*) and maximum-likelihood phylogenies. *act-1* and *act-3* are identical, while *act-5* is the most, but only slightly, divergent gene.

(**D**) Genomic organization of *act-5*, *arx-2,* and *unc-60*; germline deletions; and genotypes of germline mutant strains used in this study. All genes have exonic deletions and are predicted, or have been demonstrated, to be genetic nulls. *act-5(ok1397)* also deletes the *act-5* promoter; *unc-60(gk239)* affects the UNC60A isoform that is expressed in embryonic and non-muscle tissues, including the intestine (*14*). Note that all genes are maternal-effect embryonic/larval lethal, hence require balancers that will provide maternal product to the homozygous mutant progeny.

Anterior-posterior apical membrane extension during intestinal development

A

	Length	SEM
Bean	21.62	0.3
L1	80.26	2.822
L2/3	243.6	8.303
L4	684.6	15.49
Adult	735.1	32.31

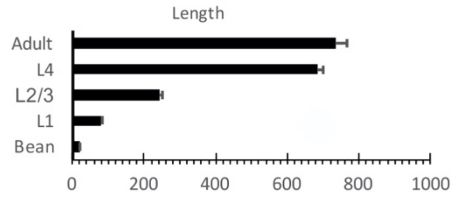

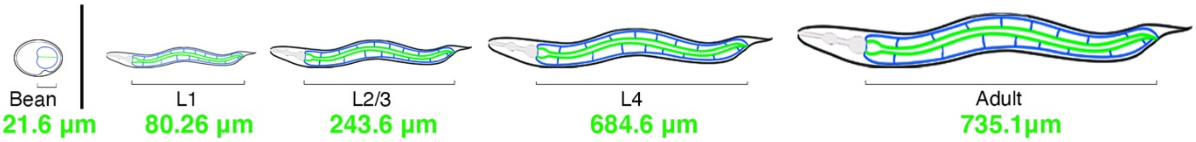

B

	Length	SEM
Bean	21.62	0.3
Comma	24.71	0.288
1.5 fold	35.92	0.27
2-fold	54.1	0.608
3-fold	67.05	0.894

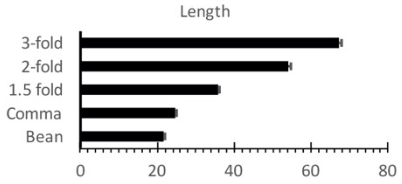

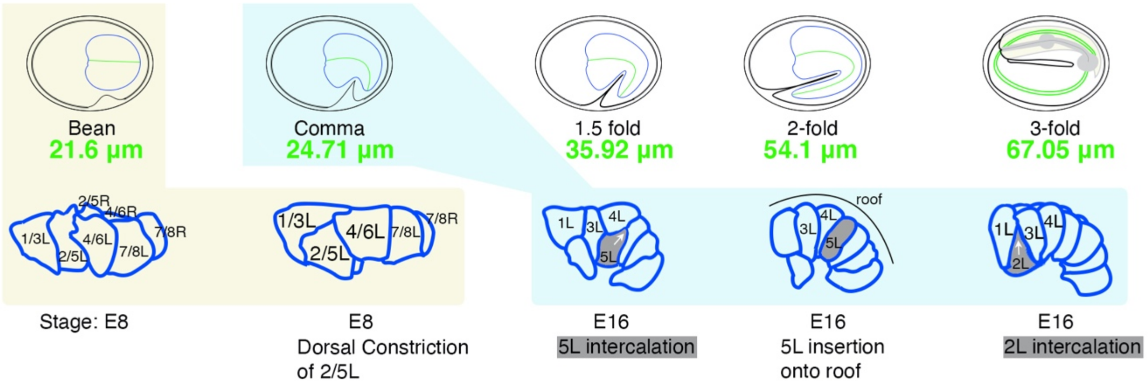

Glossary of terms used for different defects in apical membrane modeling.

Defects in apical membrane positioning: apicobasal membrane polarity defects.

**Polarity conversion**	Basolateral mislocalization of integral or submembranous apical membrane or junction components (e.g., Fig.1B, E).
**Ectopic basolateral lumens**	Full transformation of basolateral into apical membrane, with microvilli, submembranous undercoat (ERM-1+), and surrounding junctions (e.g., Fig. 1.C ,F, Y, Z: surrounding junctions require confirmation, e.g., by TEM).

Defects in apical membrane structure, assembly and expansion: apical membrane biogenesis defects.

**Ectopic intracellular lumens**	Cytoplasmic inclusions surrounded by full apical membrane with microvilli and submembranous undercoat (ERM-1+), no surrounding junctions (not identified in this study; requires confirmation by TEM).
**Apical vacuoles**	Cytoplasmic, typically ERM-1+ apical membrane-bound inclusion, no microvilli, no junctions (e.g., Fig.2F ; requires TEM for confirmation).
**Apical membrane blebs**	Membrane blisters contiguous with main apical (lumenal) membrane and all its characteristics (microvilli, ERM-1+ undercoat), no junctions (e.g., Fig.1F: contiguity of "bleb" with lumen is shown by red color = lumenal dsRed bacteria).

Figure S3. Embryonic and larval development of the *C. elegans* intestine and net extension of the intestinal apical (lumenal) membrane. Glossary of terms for distinct apical membrane domain biogenesis and positioning defects.

(A) Apical membrane expansion from L1-larva to adult. Anatomy of the mature post-mitotic intestine. In the mature intestine, 20 cells form 9 INT (*int*estinal) rings, consisting of two pairs of cells each that surround the lumen (except for four cells in INT1) (*26, 116*).

We measured an increase in apical membrane length from 80.26µm (L1) to 735.1µm (adult), corresponding to an almost 10-fold net increase in apical membrane from 8.9µm per cell to 81.7µm per cell (9 cells per row; Material and Methods).

Schematics show the mature single-layered intestinal epithelium in the hatched animal, with an invariant set of cells that do not further divide but continue to grow through four larval stages (only minimal further growth in adults). Here and below: apical membrane: green, basolateral membrane: blue; anterior left, posterior right; dorsal up, ventral down. Head with pharynx is indicated.

(B) Apical membrane expansion during embryogenesis. Intestinal morphogenesis (intercalation). During embryogenesis, most cells lose volume to accommodate cell divisions in the eggshell. Intestinal cells, clonally derived from the progenitor E cell, become larger relative to other cells but lose size relative to their early progenitors. The mature, bilaterally symmetrical intestinal tube is formed and elongated by one intercalation step of a lower tier of cells into an upper tier of cells, a step that occurs in parallel on the right and left side (*26, 116*).

We measured an increase in apical membrane length from 21.6µm in the bean-stage embryo (pre-intercalation intestine at the E8 to E16 stage = 5 cells per row; cells will still divide and move) to 67.05µm in the 3-fold embryo (post-intercalation intestine at the E16 to E20 stage; final set of 9 cells per row; cells no longer divide, nor move), corresponding to a net increase in apical membrane from 4.32µm to 7.45µm per cell (Material and Methods). The length of the apical membrane in cells of the hatched L1 larva (8.9µm per cell) has more than doubled compared with the apical membrane of cells in the bean stage embryo. These measurements do not consider the amount of membrane required to extend the increasing length and number of apical-membrane-specific microdomains (microvilli).

The schematics show intestinal morphogenesis from the pre-bean/bean to the 3-fold embryonic stage, with the pre-intercalation intestine (E8 stage; shaded yellow) and the intercalating intestine (E16 stage; shaded light blue) shown beneath (*116*). The *L*eft (L) row of cells is shown. Cells in the process of intercalation are shaded in dark grey. Orientation is indicated above (in A); dorsal side=roof.

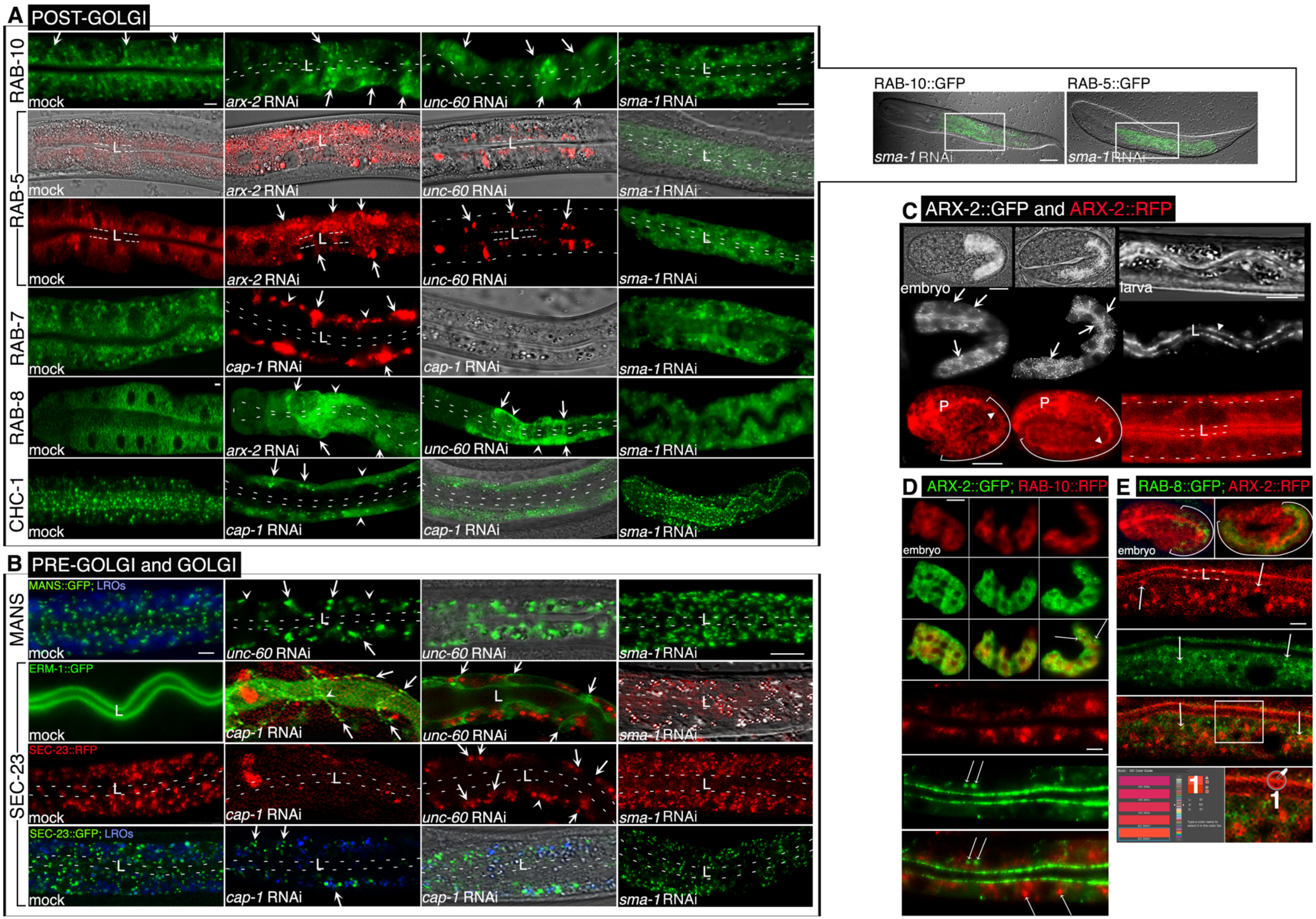
A POST-GOLGI
RAB-10
RAB-5
RAB-7
RAB-8
CHC-1
mock
arx-2 RNAi
unc-60 RNAi
sma-1 RNAi
cap-1 RNAi
RAB-10::GFP
RAB-5::GFP
B PRE-GOLGI and GOLGI
MANS
SEC-23
MANS::GFP; LROs
ERM-1::GFP
SEC-23::RFP
SEC-23::GFP; LROs
C ARX-2::GFP and ARX-2::RFP
embryo
larva
D ARX-2::GFP; RAB-10::RFP
E RAB-8::GFP; ARX-2::RFP

Figure S4. The apical positioning of pre- and post-Golgi vesicles during *de novo* polarized membrane biogenesis depends on UNC-60, ARX-2, CAP-1, and actin (related to Figure 6).

(**A** and **B**) Post-Golgi, pre-Golgi, and Golgi endo-membranes/vesicles fail to reach the apical domain and are mispositioned at basolateral membranes in late-embryonic/early-larval cells of *unc-60-, arx-2-, cap-1-,* and *actin(RNAi)*, but not of *sma-1(RNAi)*, intestines (compare Figure 6, C to E; *sma-1*/spectrin is another component of the apical cytoskeleton, used as control).

Here and below, arrows point to some of the lateral, arrowheads to some of the basal, portions of basolateral membranes; apical domain/lumen is indicated by dashed lines and/or L (*L*umen). Nomarski or Nomarski/confocal overlay images show wild-type morphology of intestines. ERM-1::GFP; SEC-23::RFP double-labeled intestines demonstrate the concomitant apical-to-basolateral mispositioning of pre-Golgi vesicles (SEC-23+) and apical membrane (ERM-1+) in *bcAM(RNAi)* intestines. Images of whole bodies of *sma-1(RNAi)* L1-larvae show typical *sma-1* elongation and head morphogenesis defects (*122*). The *sma-1* (*sma*ll worm) phenotype serves as a positive control for effective RNAi and as a negative control for the bcAM-dependent vesicle positioning defects.

(**C** to **E**) Spatiotemporal co-expression of ARX-2 with RAB-8+ and RAB-10+ vesicles during intestinal polarization (controls and additional results for Figure 6F).

(C) Comparison of ARX-2::GFP, expressed from an extrachromosomal transgene and directed to the intestine by the intestine-specific promoter *elt-2*, and ARX-2::RFP, expressed from its germline locus: matching developmental intestinal expression profile, but improved resolution of subcellular localization (membrane-associated speckles) by the extrachromosomal transgene. P: *P*harynx. The intestine is outlined by brackets and dashed lines in ARX-2::RFP animals. The increase in ARX-2::RFP background is due to a pre- and post-image acquisition increase in brightness (endogenous ARX-2 is expressed ubiquitously and at low levels). Arrows point to some of the basolateral membrane-associated ARX-2::GFP speckles, L: *L*umen, dashed line, or arrowhead.

(D) ARX-2::GFP; RAB-10::RFP double labeling fails to show a direct overlap of ARX-2 speckles with RAB-10+ vesicles (long arrows; control for ARX-2::RFP; RAB-10::GFP; Figure 6F).

(E) ARX-2::RFP speckles fail to colocalize with RAB-8::GFP+ vesicles (long arrows). The lower panel shows a magnified merged portion of the double-labeled intestine, measured by the Eyedropper tool (Adobe-22.4.3 DIC color guide, Materials and Methods; compare with Figure 6F). ARX-2::RFP brightness is increased pre- and post-image acquisition (compare Figure 6F).

Where not indicated otherwise, confocal and Nomarski/confocal overlay images of full embryonic and partial late-embryonic/early-larval intestines (two pairs of cells surrounding the lumen) are shown. See table S2 for genotypes of strains with translational fluorescent fusion proteins. Scale bars: 10 µm.

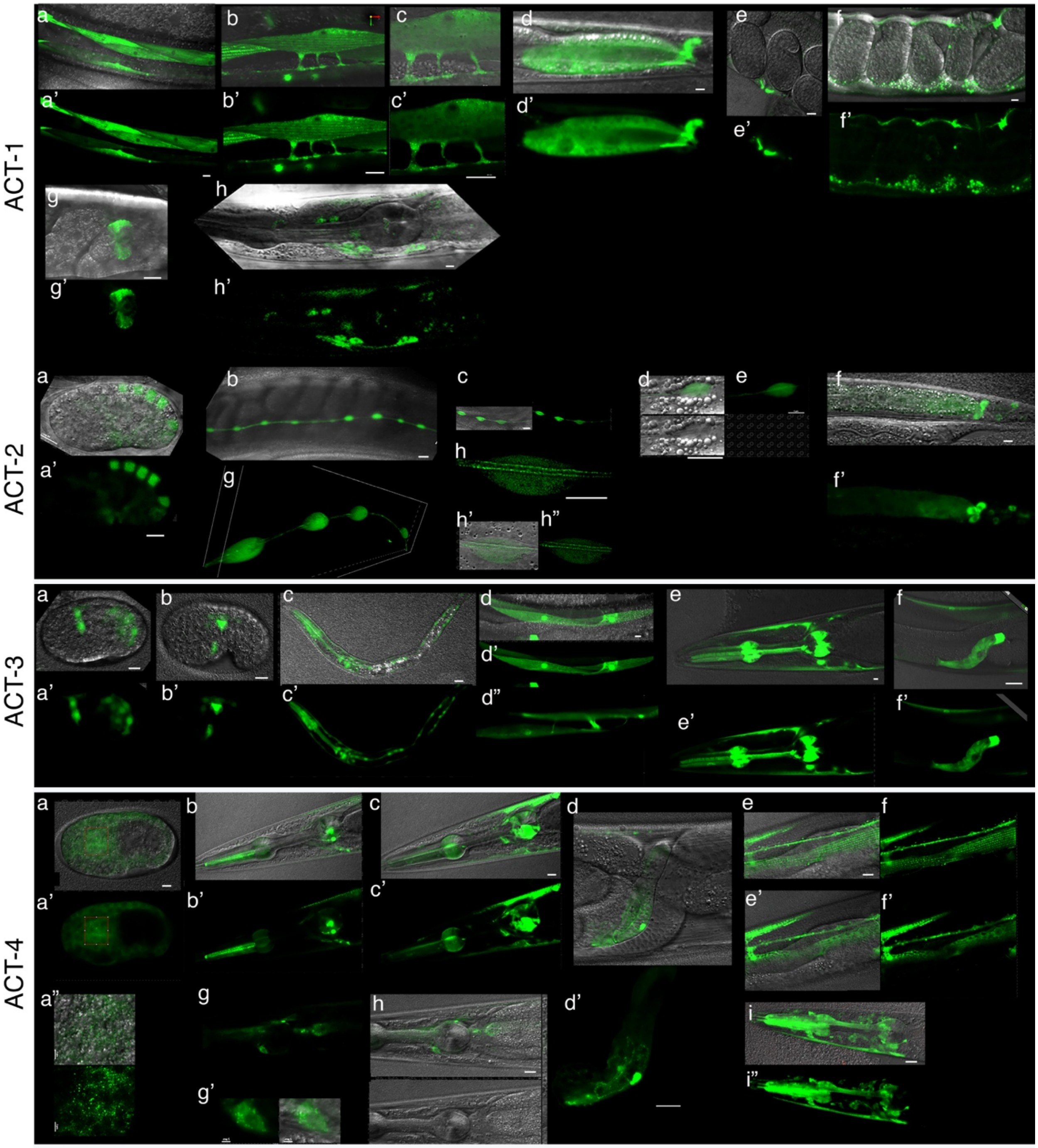

ACT-1
a
a'
b
b'
c
c'
d
d'
e
e'
f
f'
g
g'
h
h'
ACT-2
a
a'
b
g
c
h
h'
h"
d
e
f
f'
ACT-3
a
a'
b
b'
c
c'
d
d'
d"
e
e'
f
f'
ACT-4
a
a'
a"
b
b'
c
c'
d
d'
e
e'
f
f'
g
g'
h
i
i"

Figure S5. ACT-1, ACT-2, ACT-3, and ACT-4 are predominantly expressed outside of the intestine (related to Figure 7).

ACT-1::GFP: Expression in: (**A** and **A'**) body wall muscle; (**B, B', C, C'**) muscle extensions (muscle arms) that form *en passant* neuromuscular junctions (note ACT-1 puncta within these extensions); (**D** and **D'**) posterior intestine and rectal muscle cells; (**E** and **E'**) vulval muscles; (**F** and **F'**) intra-uterine ACT-1 puncta around eggshells; (**G** and **G'**) coelomocyte; (**H** and **H'**) head neurons.

ACT-2::GFP: Expression in: (**A** and **A'**) hypodermal precursors; (**B** and **C**) seam cells (magnified in **D**, **E**, and **G** [3D image] and **H** to **H"**); (**F** and **F'**) posterior intestine and rectal muscle cells. ACT-2 is also expressed in the excretory canal (not shown in these images).

ACT-3::GFP: Expression in: (**A, A', B,** and **B'**) pharyngeal and muscle cell precursors; (**C** and **C'**) pharyngeal, body wall muscle (whole L2 larva is shown); (**D** and **D")** muscles and muscle extensions (muscle arms); (**E** and **E'**) pharyngeal and body wall muscles; (**F** and **F'**) spermatheca.

ACT-4::GFP: Expression in: (**A** and **A'**) most embryonic tissues except the intestine (intracellular ACT-4 puncta magnified in **A"**); (**B, B', C,** and **C'**) pharynx, pharyngeal, and head neurons (magnified in G and G') and body wall muscles; (**D** and **D'**) spermatheca; (**E, E', F,** and **F'**) ventral nerve cord, muscle, and muscle arms; (**H**) head neurons; (**I** and **I'**) body wall muscles, pharynx, and head neurons.

Confocal and Nomarski/confocal overlay images of whole embryos and parts of larval or adult worms are shown, as indicated. Only a partial expression profile is documented.

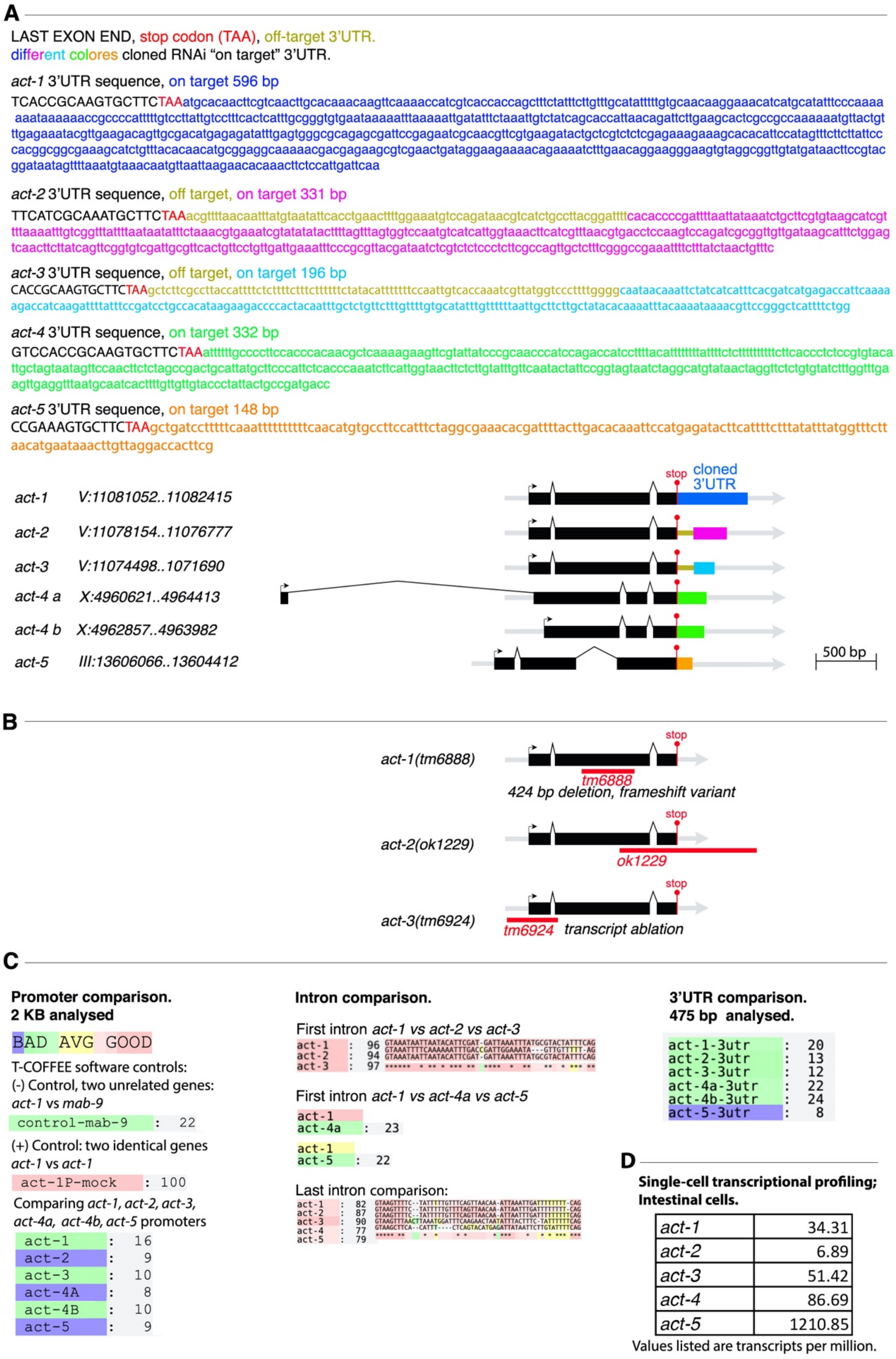

A
LAST EXON END, stop codon (TAA), off-target 3'UTR.
different colores cloned RNAi "on target" 3'UTR.
act-1 3'UTR sequence, on target 596 bp
TCACCGCAAGTGCTTCTAAatgcacaacttcgtcaacttgcacaaacaagttcaaaaccatcgtcaccaccagctttctatttcttgtttgcatatttttgtgcaacaaggaaacatcatgcatatttcccaaaa
aaataaaaaaaccgccccatttttgtccttattgtcctttcactcatttgcgggtgtgaataaaaatttaaaaattgatatttctaaattgtctatcagcaccattaacagattcttgaagcactcgccgccaaaaaatgttactgt
ttgagaaatacgttgaagacagttgcgacatgagagatatttgagtgggcgcagagcgattccgagaatcgcaacgttcgtgaagatactgctcgtctctcgagaaagaaagcacacattccatagtttcttcttattcc
cacggcggcgaaagcatctgtttacacaacatgcggaggcaaaaacgacgagaagcgtcgaactgataggaagaaaacagaaaatctttgaacaggaagggaagtgtaggcggttgtatgataacttccgtac
ggataatagttttaaatgtaaacaatgttaattaagaacacaaacttctccattgattcaa
act-2 3'UTR sequence, off target, on target 331 bp
TTCATCGCAAATGCTTCTAAacgttttaacaatttatgtaatattcacctgaacttttggaaatgtccagataacgtcatctgccttacggattttcacaccccgattttaattataaatctgcttcgtgtaagcatcgt
tttaaaatttgtcggtttattttaataatatttctaaacgtgaaatcgtatatatacttttagtttagtggtccaatgtcatcattggtaaacttcatcgtttaacgtgacctccaagtccagatcgcggttgttgataagcatttctggag
tcaacttcttatcagttcggtgtcgattgcgttcactgttcctgttgattgaaatttcccgcgttacgataatctcgtctctccctcttcgccagttgctctttcgggccgaaattttctttatctaactgtttc
act-3 3'UTR sequence, off target, on target 196 bp
CACCGCAAGTGCTTCTAAgctcttcgccttaccattttctcttttctttcttttttctatacatttttttccaattgtcaccaaatcgttatggtcccttttggggcaataacaaattctatcatcatttcacgatcatgagaccattcaaaa
agaccatcaagattttatttccgatcctgccacataagaagaccccactacaatttgctctgttctttgttttgtgcatatttgttttttaattgcttcttgctatacacaaaatttacaaaataaaacgttccgggctcattttctgg
act-4 3'UTR sequence, on target 332 bp
GTCCACCGCAAGTGCTTCTAAatttttgccccttccacccacaacgctcaaaagaagttcgtattatcccgcaacccatccagaccatccttttacattttttttatttctcttttttttttcttcaccctctccgtgtaca
ttgctagtaatagttccaacttctctagccgactgcattatgcttcccattctcacccaaatcttcattggtaacttctcttgtatttgttcaatactattccggtagtaatctaggcatgtataactaggttctctgtgtatctttggtttga
agttgaggtttaatgcaatcacttttgttgttgtaccctattactgccgatgacc
act-5 3'UTR sequence, on target 148 bp
CCGAAAGTGCTTCTAAgctgatcctttttcaaatttttttttttcaacatgtgccttccatttctaggcgaaacacgattttacttgacacaaattccatgagatacttcattttctttatatttatggtttctt
aacatgaataaacttgttaggaccacttcg
stop
cloned 3'UTR
act-1 V:11081052..11082415
act-2 V:11078154..11076777
act-3 V:11074498..1071690
act-4 a X:4960621..4964413
act-4 b X:4962857..4963982
act-5 III:13606066..13604412
500 bp
B
act-1(tm6888)
stop
tm6888
424 bp deletion, frameshift variant
act-2(ok1229)
stop
ok1229
act-3(tm6924)
stop
tm6924 transcript ablation
C
Promoter comparison.
2 KB analysed
BAD AVG GOOD
T-COFFEE software controls:
(-) Control, two unrelated genes:
act-1 vs mab-9
control-mab-9 : 22
(+) Control: two identical genes
act-1 vs act-1
act-1P-mock : 100
Comparing act-1, act-2, act-3, act-4a, act-4b, act-5 promoters
act-1 : 16
act-2 : 9
act-3 : 10
act-4A : 8
act-4B : 10
act-5 : 9
Intron comparison.
First intron act-1 vs act-2 vs act-3
act-1 : 96
act-2 : 94
act-3 : 97
First intron act-1 vs act-4a vs act-5
act-1
act-4a : 23
act-1
act-5 : 22
Last intron comparison:
act-1 : 82
act-2 : 87
act-3 : 90
act-4 : 77
act-5 : 79
3'UTR comparison.
475 bp analysed.
act-1-3utr : 20
act-2-3utr : 13
act-3-3utr : 12
act-4a-3utr : 22
act-4b-3utr : 24
act-5-3utr : 8
D
Single-cell transcriptional profiling; Intestinal cells.
act-1 34.31
act-2 6.89
act-3 51.42
act-4 86.69
act-5 1210.85
Values listed are transcripts per million.

Figure S6. ***act-1, -2, -3, -4, -5*** **sequences targeted by 3'UTR-directed double-stranded RNAs. Genomic organization of** ***act-1, -2, -3, -4, -5. act-1, -2, -3*** **germline deletions. Comparative analysis of non-coding regions of** ***act-1, -2, -3, -4, -5*** **(promoters, introns, 3'UTRs).** ***act-1, -2, -3, -4, -5*** **intestinal expression by single-cell RNA-seq (see Figure S2 for spliced sequence alignment of** ***act-1, -2, -3, -4, -5*****; Figure S7 for ACT-1, -2, -3, -4, -5 protein sequence alignment).**

(**A**) Targeted *act-1, -2, -3, -4, -5* 3'UTR sequences are highlighted in different colors. Plasmid vectors to generate the double-stranded RNAs (dsRNAs) for the bacterial RNAi feeding approach (Materials and Methods) were designed to exclude any possible off-target effects (off-target sequences are indicated in olive color). These *act-1, -2, -3, -4, -5* dsRNAs did not induce lethality or obvious phenotypes - in contrast to an *act-5* 3'UTR dsRNA used in the previous study by MacQueen *et al.* (*17*) - nor did the *act-1, -2, -3, -4* dsRNAs induce lethality as double- or triple RNAis - in contrast to the lethality reported for *act-1-* or *act-4* 3'UTR RNAi, *act-1+2-, act-2+3* 3'UTR double RNAi, *act-2[ok1229] act-1+3* 3'UTR double mutant/RNAi by Willis *et al*. (*92*).

The lower panels show intron/exon structures of *act-1, -2, -3, -4, -5* relative to the targeted 3'UTRs, and the genes' chromosomal locations. *act-1, 2,* and *3* are located on a cluster on chromosome V. Note that no alternative splice forms are predicted for *act-1, -2, -3* and *-5*, and only two alternative splice forms for *act-4*.

(**B**) Germline deletions in predicted null mutants of *act-1, -2, -3*, used in this study. These mutants are homozygous viable.

(**C**) T-COFFEE alignment of promoters, introns, and 3'UTR of the five actin isoforms. (-) and (+) controls, *mab-9* and *act-1p-mock*, respectively, are shown. Compare these to the spliced sequence alignment in Figure S2.

(**D**) Transcriptional profiling of actin isoforms in intestinal cells by single-cell RNA-seq (*123*).

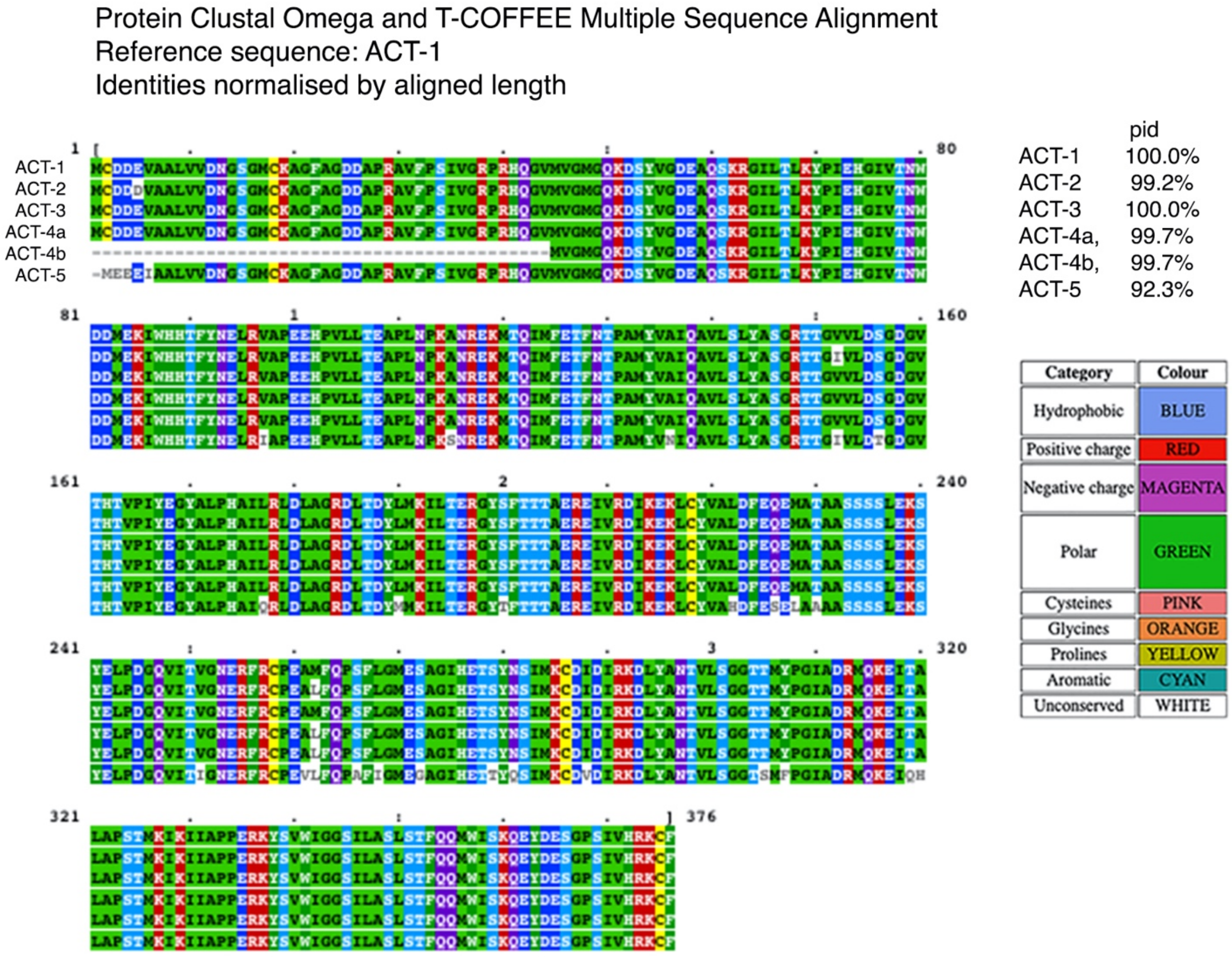

Figure S7. Sequence alignment of ACTIN proteins.

ACT-1 and ACT-3 are identical, distinguished by only two amino acids from ACT-2, by only one amino acid from ACT-4 (each of the two isoforms); by 24 amino acids from ACT-5. A comparison of Clustal Omega and T-COFFEE alignment is shown (compare to the spliced sequence alignment in Figure S2). Note that alignment is based on identity, not similarity.

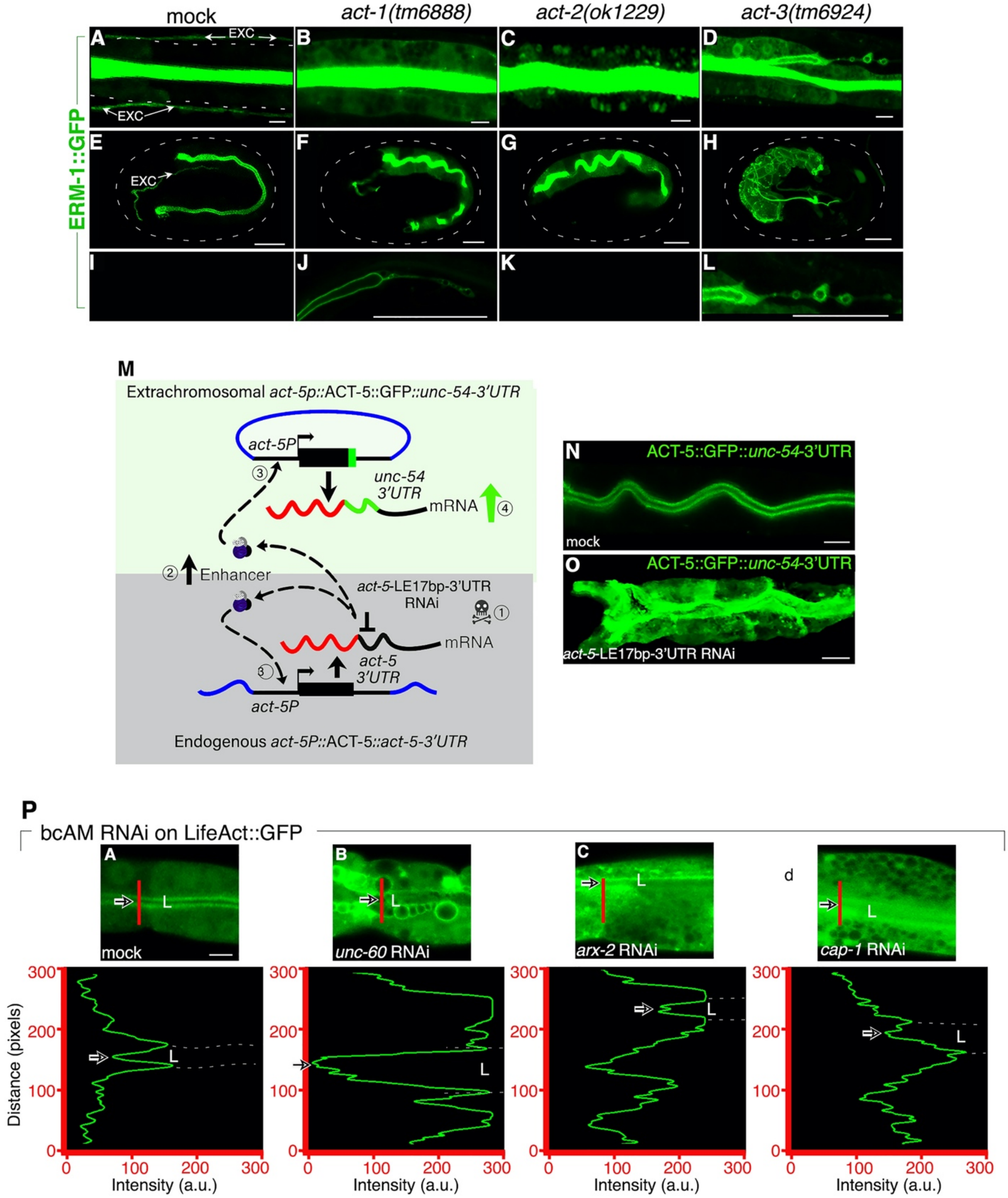

mock
act-1(tm6888)
act-2(ok1229)
act-3(tm6924)
ERM-1::GFP
A
B
C
D
E
F
G
H
I
J
K
L
EXC
M
Extrachromosomal act-5p::ACT-5::GFP::unc-54-3'UTR
act-5P
unc-54
3'UTR
mRNA
④
③
② Enhancer
act-5-LE17bp-3'UTR
RNAi
①
mRNA
act-5
3'UTR
act-5P
Endogenous act-5P::ACT-5::act-5-3'UTR
N
ACT-5::GFP::unc-54-3'UTR
mock
O
ACT-5::GFP::unc-54-3'UTR
act-5-LE17bp-3'UTR RNAi
P
bcAM RNAi on LifeAct::GFP
A
mock
B
unc-60 RNAi
C
arx-2 RNAi
d
cap-1 RNAi
L
Distance (pixels)
Intensity (a.u.)
0
100
200
300

Figure S8. Effect of *act-1, -2, -3* germline mutants on intestinal and excretory canal apical domain (lumen) biogenesis. Trans-activational increase of ACT-5::GFP-*unc-54*-3'UTR expression by *act-5-LE17bp* 3'UTR RNAi. Quantification of LifeACT displacement from the apical domain by *bcAM* RNAi.

(**A** to **L**) **Effect of *act-1, -2, -3* germline mutants on intestinal and excretory canal apical domain (lumen) biogenesis.**

(A) ERM-1::GFP at wild-type (mock) larval intestinal and excretory canal apical/lumenal membranes (one excretory canal arm each stretches alongside the basal side of the intestine; see Figure 1W1 for canal anatomy). Dashed lines mark the intestinal basal border, EXC marks the excretory canal arms.

(B to D) ERM-1::GFP displacement in mutant larval intestines: (B) *act-1(tm6888)*: faint homogenous cytoplasmic and perinuclear displacement (41% of animals); (C) *act-2(ok1229)*: homogenous and punctate cytoplasmic displacement (45% of animals); (D) *act-3(tm6924)*: cytoplasmic displacement (50% of animals; occasional canal lumen defects in B and D are enlarged in J and L). Mild intestinal lumenogenesis defects (e.g., apical membrane blebs) are present in all three mutants.

(E) ERM-1::GFP at wild-type (mock) late-embryonic intestinal and excretory canal apical/lumenal membranes (the canal [EXC] is visible as not yet fully extended H-shaped structure in E, F, and H).

(F to H) Cytoplasmic ERM-1::GFP displacement in the majority of mutant embryonic intestines and additional punctate and basolateral ERM-1 displacement in *act-3(tm6924)* embryo (penetrance <1%).

(J to L) Examples of occasional cystic excretory canal defects at higher magnification. Confocal projections of two sets of cells surrounding the lumen of larval intestines are shown in (A to D), whole embryos in (E to H) (eggshell is indicated by dashed line).

(**M** to **O**) **Trans-activational increase of ACT-5::GFP-*unc-54*-3'UTR expression by *act-5-LE17bp* 3'UTR RNAi.**

(M) Schematic for a proposed trans-activational increase in ACT-5::GFP-*unc-54*-3'UTR expression, as shown in panel (N) and (O), by *act-5-LE17bp* 3'UTR RNAi (see Figure S2B for construct). *act-5-LE17bp* 3'UTR RNAi is directed against the endogenous *act-5* 3'UTR that is missing in the exogenously expressed ACT-5::GFP-*unc-54*-3'UTR. Note that this mechanism is different from the intergenic transcriptional adaptation between *act-5* and *act-3* that results in (*124*) ectopic promoter activation and is mediated by a direct effect of a mutant RNA on transcription. Transcriptional adaption, on the other hand, could contribute to the comparatively minor effect of the *act-5(dt2017/dt2019*) mutant alleles on intestinal morphogenesis and viability (*17*).

(N to O) Increase in ACT-5::GFP-*unc-54*-3'UTR expression in the larval intestine by *act-5-LE17bp* 3'UTR RNAi, with concomitant cytoplasmic and basolateral ACT-5::GFP mislocalization, induced by interference with the endogenous *act-5*. Note that ACT-5::GFP overexpression by itself does not result in basolateral ERM-1 displacement. Also note that transgenic ACT-5::GFP-*unc-54*-3'UTR only partially rescues the germline *act-5* deletion. We suggest that either the GFP interferes with the bcAM-dependent filament modulation that drives the vectorial F-actin movement (Figures 9 and S9) or that the 3'UTR is required for this function. These are two possible explanation for the inability of ACT-5::GFP-*unc-54*-3'UTR to rescue the actin-dependent polarity defect yet support actin assembly at the apical domain (the larger fraction

of apical ACT-5 becomes enriched at the apical domain after membrane polarization is complete; compare Figure 4).

(P) Quantification of LifeACT displacement from the apical domain by bcAM RNAi.

Upper panel: LifeACT expression in a pair of cells surrounding the lumen (L) of wild-type (mock) (A) and of bcAM-depleted (B to D) larval intestines. The red lines indicate the measured area of expression intensity, reflected in the panels below. In the lower panel, two spikes of expression correspond to the lumen (L; dashed lines); the arrows indicate the middle of the lumen.

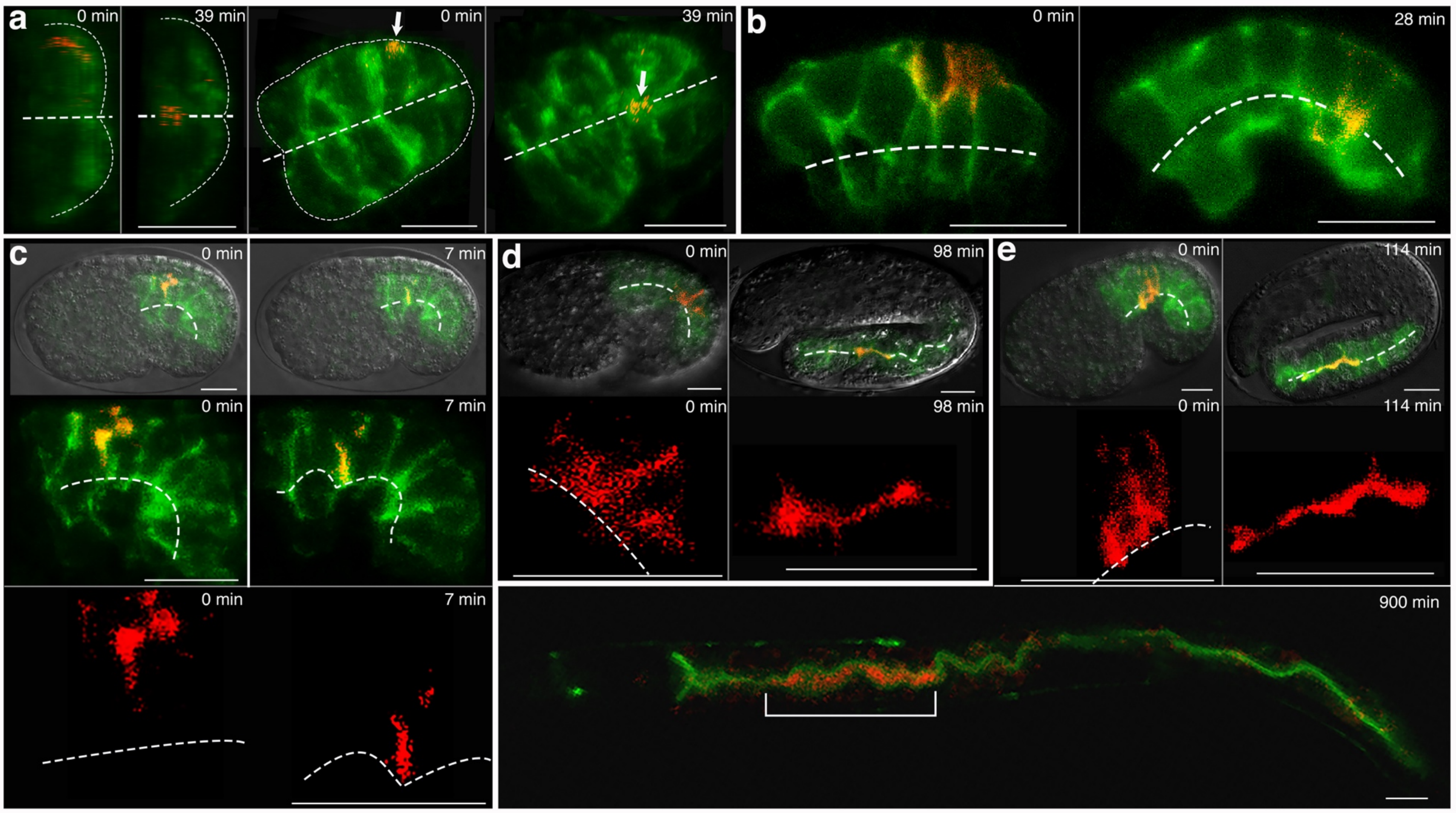

a
0 min
39 min
0 min
39 min
b
0 min
28 min
c
0 min
7 min
0 min
7 min
0 min
7 min
d
0 min
98 min
0 min
98 min
e
0 min
114 min
0 min
114 min
900 min

Figure S9. *In vivo* tracking of the basolateral-to-apical F-actin shift in single cells by photo-conversion using LifeACT::Dendra2 (compare to LifeACT::PA-GFP; Figure 9).

elt-2-driven intestinal LifeACT::Dendra2 can be photo-converted from green to red, allowing for concomitant tracking of converted LifeACT::Dendra2 (red; only once generated/converted from green) and non-converted LifeACT::Dendra2 (green = *elt-2*-driven, ongoing expression).

(**A**) Photo-conversion of the basal portion of the basolateral membrane of a single cell of the pre-intercalation intestine during polarity establishment (early pre-bean stage). In the first two panels, ventral is to the left and dorsal to the right; the third and fourth panels (from left) show a dorsal view, with anterior to the left and posterior to the right. Note that the converted LifeACT::Dendra2 (red; arrow) only shows previously generated, not newly-assembled, F-actin. Also note that photo-converted LifeACT::Dendra2 (red) does not dispers to other areas of F-actin, labeled by non-converted LifeACT::Dendra2 (green), validating photomodulated LifeACT as a tracer for F-actin movement (the same applies to LifeACT::PA-GFP (green) in a LifeACT::mCherry (red) background; Figure 9). Thus F-actin moves from basolateral (red, 0 min) to the midline (dashed line: future apical domain/lumen; red, 39 min). The 3D rotating view of this embryo is shown in Movie 1.

(**B**) Photo-conversion of basolateral membranes and the basal cytoplasm of two cells during late intercalation (comma stage), revealing F-actin movement to the apical domain (dashed line) after 28 min. Lateral view: anterior is to the left, and posterior is to the right.

(**C**) F-actin movement can be tracked from the basolateral membrane angle along the lateral membrane at a shorter time interval during intestinal intercalation in a late bean-stage embryo (7 minutes post photo-conversion). Converted LifeACT::Dendra2 (red) is shown beneath at higher magnification. The 3D rotating view of this embryo is shown in Movie 2.

(**D**) Photo-conversion of the lateral portion of the basolateral membranes and cytoplasm of two adjacent cells in the post-intercalation intestine (1.5-fold embryo) reveals the cytoplasmic-to-apical and lateral-to-apical F-actin shift after 98 minutes post photo-conversion (2-fold embryo).

(**E**) Tracking of LifeACT::Dendra2 (red), photo-converted at a later stage of intestinal intercalation (comma stage), from the apico-lateral angle between the E2 and E3 cell through an extended period of time (0-114-900 min). At the L1-larval stage, 900 minutes after post-photo-conversion, converted LifeACT::Dendra2 (red) is restricted to the apical domain (lumen; bracketed in the lower panel).

Table S1. ACT-5 cooperates with other actin isoforms in apical domain positioning in the *C. elegans* intestine.
Table S2. Strain list.
Table S3. Scaled-intensity RNAi. Annotation to Figures.
Movie 1. A 3D rotating view of the embryo from Figure S9A.
Movie 2. A 3D rotating view of the embryo from Figure S9C.

REFERENCES AND NOTES

1. E. Rodriguez-Boulan, I. G. Macara, Organization and execution of the epithelial polarity programme. *Nat. Rev. Mol. Cell Biol.* **15**, 225–242 (2014).

2. C. E. Buckley, D. St Johnston, Apical-basal polarity and the control of epithelial form and function. *Nat. Rev. Mol. Cell Biol.* **23**, 559–577 (2022).

3. I. Mellman, W. J. Nelson, Coordinated protein sorting, targeting and distribution in polarized cells. *Nat. Rev. Mol. Cell Biol.* **9**, 833–845 (2008).

4. D. C. Anderson, J. S. Gill, R. M. Cinalli, J. Nance, Polarization of the *C. elegans* embryo by RhoGAP-mediated exclusion of PAR-6 from cell contacts. *Science* **320**, 1771–1774 (2008).

5. J. Chen, A. C. Sayadian, N. Lowe, H. E. Lovegrove, D. St Johnston, An alternative mode of epithelial polarity in the *Drosophila* midgut. *PLOS Biol.* **16**, e3000041 (2018).

6. D. M. Bryant, A. Datta, A. E. Rodríguez-Fraticelli, J. Peränen, F. Martín-Belmonte, K. E. Mostov, A molecular network for de novo generation of the apical surface and lumen. *Nat. Cell Biol.* **12**, 1035–1045 (2010).

7. H. Zhang, N. Abraham, L. A. Khan, D. H. Hall, J. T. Fleming, V. Göbel, Apicobasal domain identities of expanding tubular membranes depend on glycosphingolipid biosynthesis. *Nat. Cell Biol.* **13**, 1189–1201 (2011).

8. M. Shafaq-Zadah, L. Brocard, F. Solari, G. Michaux, AP-1 is required for the maintenance of apico-basal polarity in the C. elegans intestine. *Development* **139**, 2061–2070 (2012).

9. J. M. Shivas, A. R. Skop, Arp2/3 mediates early endosome dynamics necessary for the maintenance of PAR asymmetry in *Caenorhabditis elegans*. *Mol. Biol. Cell* **23**, 1917–1927 (2012).

10. H. Zhang, A. Kim, N. Abraham, L. A. Khan, D. H. Hall, J. T. Fleming, V. Gobel, Clathrin and AP-1 regulate apical polarity and lumen formation during *C. elegans* tubulogenesis. *Development* **139**, 2071–2083 (2012).

11. S. Eaton, F. Martin-Belmonte, Cargo sorting in the endocytic pathway: A key regulator of cell polarity and tissue dynamics. *Cold Spring Harb. Perspect. Biol.* **6**, a016899 (2014).

12. C. E. Jewett, R. Prekeris, Insane in the apical membrane: Trafficking events mediating apicobasal epithelial polarity during tube morphogenesis. *Traffic* **19**, 666–678 (2018).

13. N. Zhang, H. Zhang, L. A. Khan, G. Jafari, Y. Eun, E. Membreno, V. Gobel, The biosynthetic-secretory pathway, supplemented by recycling routes, specifies epithelial membrane polarity. *Sci. Adv.* (2023).

14. K. Ono, M. Parast, C. Alberico, G. M. Benian, S. Ono, Specific requirement for two ADF/cofilin isoforms in distinct actin-dependent processes in Caenorhabditis elegans. *J. Cell Sci.* **116**, 2073–2085 (2003).

15. J. L. Feldman, J. R. Priess, A role for the centrosome and PAR-3 in the hand-off of MTOC function during epithelial polarization. *Curr. Biol.* **22**, 575–582 (2012).

16. M. A. Pickett, V. F. Naturale, J. L. Feldman, A polarizing issue: Diversity in the mechanisms underlying apico-basolateral polarization in vivo. *Annu. Rev. Cell Dev. Biol.* **35**, 285–308 (2019).

17. A. J. MacQueen, J. J. Baggett, N. Perumov, R. A. Bauer, T. Januszewski, L. Schriefer, J. A. Waddle, ACT-5 is an essential Caenorhabditis elegans actin required for intestinal microvilli formation. *Mol. Biol. Cell* **16**, 3247–3259 (2005).

18. E. D. Goley, M. D. Welch, The ARP2/3 complex: An actin nucleator comes of age. *Nat. Rev. Mol. Cell Biol.* **7**, 713–726 (2006).

19. K. Rottner, J. Faix, S. Bogdan, S. Linder, E. Kerkhoff, Actin assembly mechanisms at a glance. *J. Cell Sci.* **130**, 3427–3435 (2017).

20. L. Hu, G. A. Papoian, Mechano-chemical feedbacks regulate actin mesh growth in lamellipodial protrusions. *Biophys. J.* **98**, 1375–1384 (2010).

21. Y. Y. Bernadskaya, F. B. Patel, H. T. Hsu, M. C. Soto, Arp2/3 promotes junction formation and maintenance in the *Caenorhabditis elegans* intestine by regulating membrane association of apical proteins. *Mol. Biol. Cell* **22**, 2886–2899 (2011).

22. S. Sasidharan, S. Borinskaya, F. Patel, Y. Bernadskaya, S. Mandalapu, M. Agapito, M. C. Soto, WAVE regulates cadherin junction assembly and turnover during epithelial polarization. *Dev. Biol.* **434**, 133–148 (2018).

23. L. Cordova-Burgos, F. B. Patel, M. C. Soto, E-cadherin/HMR-1 membrane enrichment is polarized by WAVE-dependent branched actin. *J. Dev. Biol.* **9**, 19 (2021).

24. A. Achilleos, A. M. Wehman, J. Nance, PAR-3 mediates the initial clustering and apical localization of junction and polarity proteins during C. elegans intestinal epithelial cell polarization. *Development* **137**, 1833–1842 (2010).

25. R. Totong, A. Achilleos, J. Nance, PAR-6 is required for junction formation but not apicobasal polarization in C. elegans embryonic epithelial cells. *Development* **134**, 1259–1268 (2007).

26. B. Leung, G. J. Hermann, J. R. Priess, Organogenesis of the Caenorhabditis elegans intestine. *Dev. Biol.* **216**, 114–134 (1999).

27. K. R. Myers, J. E. Casanova, Regulation of actin cytoskeleton dynamics by Arf-family GTPases. *Trends Cell Biol.* **18**, 184–192 (2008).

28. J. N. Brandt, L. Voss, F. M. Rambo, K. Nicholson, J. R. Thein, L. Fairchild, L. Seabrook, D. Lewis, L. Guevara-Hernandez, M. L. White, L. Sax, V. Eichten, L. Harper, G. J. Hermann, Asymmetric organelle positioning during epithelial polarization of C. elegans intestinal cells. *Dev. Biol.* **481**, 75–94 (2022).

29. E. Rodriguez-Boulan, G. Kreitzer, A. Musch, Organization of vesicular trafficking in epithelia. *Nat. Rev. Mol. Cell Biol.* **6**, 233–247 (2005).

30. K. Sato, A. Norris, M. Sato, B. D. Grant, C. elegans as a model for membrane traffic. *WormBook*, 1–47 (2014).

31. G. J. Hermann, L. K. Schroeder, C. A. Hieb, A. M. Kershner, B. M. Rabbitts, P. Fonarev, B. D. Grant, J. R. Priess, Genetic analysis of lysosomal trafficking in *Caenorhabditis elegans*. *Mol. Biol. Cell* **16**, 3273–3288 (2005).

32. L. Cramer, Organelle transport: Dynamic actin tracks for myosin motors. *Curr. Biol.* **18**, R1066–R1068 (2008).

33. R. Massarwa, E. D. Schejter, B. Z. Shilo, Apical secretion in epithelial tubes of the drosophila embryo is directed by the Formin-family protein diaphanous. *Dev. Cell* **16**, 877–888 (2009).

34. E. Geron, E. D. Schejter, B. Z. Shilo, Directing exocrine secretory vesicles to the apical membrane by actin cables generated by the formin mDia1. *Proc. Natl. Acad. Sci. U.S.A.* **110**, 10652–10657 (2013).

35. A. Bidaud-Meynard, F. Demouchy, O. Nicolle, A. Pacquelet, S. K. Suman, C. N. Plancke, F. B. Robin, G. Michaux, High-resolution dynamic mapping of the C. elegans intestinal brush border. *Development* **148**, dev200029 (2021).

36. K. Sliogeryte, S. D. Thorpe, Z. Wang, C. L. Thompson, N. Gavara, M. M. Knight, Differential effects of LifeAct-GFP and actin-GFP on cell mechanics assessed using micropipette aspiration. *J. Biomech.* **49**, 310–317 (2016).

37. A. Rajan, A. C. Tien, C. M. Haueter, K. L. Schulze, H. J. Bellen, The Arp2/3 complex and WASp are required for apical trafficking of Delta into microvilli during cell fate specification of sensory organ precursors. *Nat. Cell Biol.* **11**, 815–824 (2009).

38. M. Schuh, An actin-dependent mechanism for long-range vesicle transport. *Nat. Cell Biol.* **13**, 1431–1436 (2011).

39. Z. Holubcova, G. Howard, M. Schuh, Vesicles modulate an actin network for asymmetric spindle positioning. *Nat. Cell Biol.* **15**, 937–947 (2013).

40. I. R. Boldogh, H. C. Yang, W. D. Nowakowski, S. L. Karmon, L. G. Hays, J. R. Yates III, L. A. Pon, Arp2/3 complex and actin dynamics are required for actin-based mitochondrial motility in yeast. *Proc. Natl. Acad. Sci. U.S.A.* **98**, 3162–3167 (2001).

41. A. S. Moore, S. M. Coscia, C. L. Simpson, F. E. Ortega, E. C. Wait, J. M. Heddleston, J. J. Nirschl, C. J. Obara, P. Guedes-Dias, C. A. Boecker, T. L. Chew, J. A. Theriot, J. Lippincott-Schwartz, E. L. F. Holzbaur, Actin cables and comet tails organize mitochondrial networks in mitosis. *Nature* **591**, 659–664 (2021).

42. J. Taunton, B. A. Rowning, M. L. Coughlin, M. Wu, R. T. Moon, T. J. Mitchison, C. A. Larabell, Actin-dependent propulsion of endosomes and lysosomes by recruitment of N-WASP. *J. Cell Biol.* **148**, 519–530 (2000).

43. T. D. Nightingale, D. F. Cutler, L. P. Cramer, Actin coats and rings promote regulated exocytosis. *Trends Cell Biol.* **22**, 329–337 (2012).

44. K. Dahlgaard, A. A. Raposo, T. Niccoli, D. St Johnston, Capu and spire assemble a cytoplasmic actin mesh that maintains microtubule organization in the *Drosophila* oocyte. *Dev. Cell* **13**, 539–553 (2007).

45. M. Mori, N. Monnier, N. Daigle, M. Bathe, J. Ellenberg, P. Lénárt, Intracellular transport by an anchored homogeneously contracting F-actin meshwork. *Curr. Biol.* **21**, 606–611 (2011).

46. P. Bun, S. Dmitrieff, J. M. Belmonte, F. J. Nedelec, P. Lenart, A disassembly-driven mechanism explains F-actin-mediated chromosome transport in starfish oocytes. *eLife* **7**, e31469 (2018).

47. C. Kocks, R. Hellio, P. Gounon, H. Ohayon, P. Cossart, Polarized distribution of listeria monocytogenes surface protein ActA at the site of directional actin assembly. *J. Cell Sci.* **105**, 699–710 (1993).

48. K. L. Fehrenbacher, I. R. Boldogh, L. A. Pon, Taking the A-train: Actin-based force generators and organelle targeting. *Trends Cell Biol.* **13**, 472–477 (2003).

49. D. Serwas, M. Akamatsu, A. Moayed, K. Vegesna, R. Vasan, J. M. Hill, J. Schöneberg, K. M. Davies, P. Rangamani, D. G. Drubin, Mechanistic insights into actin force generation during vesicle formation from cryo-electron tomography. *Dev. Cell* **57**, 1132–1145.e5 (2022).

50. R. Nagai, L. I. Rebhun, Cytoplasmic microfilaments in streaming Nitella cells. *J. Ultrastruct. Res.* **14**, 571–589 (1966).

51. L. Yolland, M. Burki, S. Marcotti, A. Luchici, F. N. Kenny, J. R. Davis, E. Serna-Morales, J. Müller, M. Sixt, A. Davidson, W. Wood, L. J. Schumacher, R. G. Endres, M. Miodownik, B. M. Stramer, Persistent and polarized global actin flow is essential for directionality during cell migration. *Nat. Cell Biol.* **21**, 1370–1381 (2019).

52. S. Shamipour, R. Kardos, S.L. Xue, B. Hof, E. Hannezo, C. P. Heisenberg, Bulk actin dynamics drive phase segregation in zebrafish oocytes. *Cell* **177**, 1463–1479.e18 (2019).

53. S. Shamipour, S. Caballero-Mancebo, C. P. Heisenberg, Cytoplasm's got moves. *Dev. Cell* **56**, 213–226 (2021).

54. M. Almonacid, W. W. Ahmed, M. Bussonnier, P. Mailly, T. Betz, R. Voituriez, N. S. Gov, M. H. Verlhac, Active diffusion positions the nucleus in mouse oocytes. *Nat. Cell Biol.* **17**, 470–479 (2015).

55. A. Colin, G. Letort, N. Razin, M. Almonacid, W. Ahmed, T. Betz, M. E. Terret, N. S. Gov, R. Voituriez, Z. Gueroui, M. H. Verlhac, Active diffusion in oocytes nonspecifically centers large objects during prophase I and meiosis I. *J. Cell Biol.* **219**, e201908195 (2020).

56. A. Chaigne, C. Campillo, R. Voituriez, N. S. Gov, C. Sykes, M. H. Verlhac, M. E. Terret, F-actin mechanics control spindle centring in the mouse zygote. *Nat. Commun.* **7**, 10253 (2016).

57. E. Munro, J. Nance, J. R. Priess, Cortical flows powered by asymmetrical contraction transport PAR proteins to establish and maintain anterior-posterior polarity in the early C. elegans embryo. *Dev. Cell* **7**, 413–424 (2004).

58. A. A. Cuenca, A. Schetter, D. Aceto, K. Kemphues, G. Seydoux, Polarization of the *C. elegans* zygote proceeds via distinct establishment and maintenance phases. *Development* **130**, 1255–1265 (2003).

59. M. Mayer, M. Depken, J. S. Bois, F. Julicher, S. W. Grill, Anisotropies in cortical tension reveal the physical basis of polarizing cortical flows. *Nature* **467**, 617–621 (2010).

60. D. J. Dickinson, F. Schwager, L. Pintard, M. Gotta, B. Goldstein, A single-cell biochemistry approach reveals PAR complex dynamics during cell polarization. *Dev. Cell* **42**, 416–434.e11 (2017).

61. Y. Li, E. Munro, Filament-guided filament assembly provides structural memory of filament alignment during cytokinesis. *Dev. Cell* **56**, 2486–2500.e6 (2021).

62. W. J. Nelson, Adaptation of core mechanisms to generate cell polarity. *Nature* **422**, 766–774 (2003).

63. B. Goldstein, I. G. Macara, The PAR proteins: Fundamental players in animal cell polarization. *Dev. Cell* **13**, 609–622 (2007).

64. C. Zihni, E. Vlassaks, S. Terry, J. Carlton, T. K. C. Leung, M. Olson, F. Pichaud, M. S. Balda, K. Matter, An apical MRCK-driven morphogenetic pathway controls epithelial polarity. *Nat. Cell Biol.* **19**, 1049–1060 (2017).

65. R. Andrews, J. Ahringer, Asymmetry of early endosome distribution in *C. elegans* embryos. *PLOS ONE* **2**, e493 (2007).

66. H. Xiong, W. A. Mohler, M. C. Soto, The branched actin nucleator Arp2/3 promotes nuclear migrations and cell polarity in the *C. elegans* zygote. *Dev. Biol.* **357**, 356–369 (2011).

67. M. A. Pickett, M. D. Sallee, L. Cote, V. F. Naturale, D. Akpinaroglu, J. Lee, K. Shen, J. L. Feldman, Separable mechanisms drive local and global polarity establishment in the Caenorhabditiselegans intestinal epithelium. *Development* **149**, dev200325 (2022).

68. N. Tamehiro, Z. Mujawar, S. Zhou, D. Z. Zhuang, T. Hornemann, A. von Eckardstein, M. L. Fitzgerald, Cell polarity factor Par3 binds SPTLC1 and modulates monocyte serine palmitoyltransferase activity and chemotaxis. *J. Biol. Chem.* **284**, 24881–24890 (2009).

69. J. P. ten Klooster, M. Jansen, J. Yuan, V. Oorschot, H. Begthel, V. di Giacomo, F. Colland, J. de Koning, M. M. Maurice, P. Hornbeck, H. Clevers, Mst4 and Ezrin induce brush borders downstream of the Lkb1/Strad/Mo25 polarization complex. *Dev. Cell* **16**, 551–562 (2009).

70. K. Ebnet, S. Iden, V. Gerke, A. Suzuki, Regulation of epithelial and endothelial junctions by PAR proteins. *Front. Biosci.* **13**, 6520–6536 (2008).

71. S. T. Armenti, E. Chan, J. Nance, Polarized exocyst-mediated vesicle fusion directs intracellular lumenogenesis within the C. elegans excretory cell. *Dev. Biol.* **394**, 110–121 (2014).

72. P. Novick, D. Botstein, Phenotypic analysis of temperature-sensitive yeast actin mutants. *Cell* **40**, 405–416 (1985).

73. J. H. Yu, A. H. Crevenna, M. Bettenbuhl, T. Freisinger, R. Wedlich-Soldner, Cortical actin dynamics driven by formins and myosin V. *J. Cell Sci.* **124**, 1533–1541 (2011).

74. R. Wedlich-Soldner, S. Altschuler, L. Wu, R. Li, Spontaneous cell polarization through actomyosin-based delivery of the Cdc42 GTPase. *Science* **299**, 1231–1235 (2003).

75. D. Ghose, D. Lew, Mechanistic insights into actin-driven polarity site movement in yeast. *Mol. Biol. Cell* **31**, 1085–1102 (2020).

76. K. Yi, J. R. Unruh, M. Deng, B. D. Slaughter, B. Rubinstein, R. Li, Dynamic maintenance of asymmetric meiotic spindle position through Arp2/3-complex-driven cytoplasmic streaming in mouse oocytes. *Nat. Cell Biol.* **13**, 1252–1258 (2011).

77. E. Peterman, R. Prekeris, The postmitotic midbody: Regulating polarity, stemness, and proliferation. *J. Cell Biol.* **218**, 3903–3911 (2019).

78. X. Bai, M. Melesse, C. G. Sorensen Turpin, D. E. Sloan, C.-Y. Chen, W.-C. Wang, P.-Y. Lee, J. R. Simmons, B. Nebenfuehr, D. Mitchell, L. R. Klebanow, N. Mattson, E. Betzig, B.-C. Chen, D. Cheerambathur, J. N. Bembenek, Aurora B functions at the apical surface after specialized cytokinesis during morphogenesis in *C. elegans*. *Development* **147**, dev181099 (2020).

79. A. Manninen, Epithelial polarity—Generating and integrating signals from the ECM with integrins. *Exp. Cell Res.* **334**, 337–349 (2015).

80. D. M. Bryant, J. Roignot, A. Datta, A. W. Overeem, M. Kim, W. Yu, X. Peng, D. J. Eastburn, A. J. Ewald, Z. Werb, K. E. Mostov, A molecular switch for the orientation of epithelial cell polarization. *Dev. Cell* **31**, 171–187 (2014).

81. J. P. Rasmussen, S. S. Reddy, J. R. Priess, Laminin is required to orient epithelial polarity in the C. elegans pharynx. *Development* **139**, 2050–2060 (2012).

82. D. P. Keeley, E. Hastie, R. Jayadev, L. C. Kelley, Q. Chi, S. G. Payne, J. L. Jeger, B. D. Hoffman, D. R. Sherwood, Comprehensive endogenous tagging of basement membrane components reveals dynamic movement within the matrix scaffolding. *Dev. Cell* **54**, 60–74.e7 (2020).

83. V. F. Naturale, M. A. Pickett, J. L. Feldman, E-cadherin/HMR-1 and PAR-3 break symmetry at stable cell contacts in a developing epithelium. bioRxiv 2022.08.10.503536 [**Preprint**]. 10 August 2022. https://doi.org/10.1101/2022.08.10.503536.

84. M. Zhu, C. Y. Leung, M. N. Shahbazi, M. Zernicka-Goetz, Actomyosin polarisation through PLC-PKC triggers symmetry breaking of the mouse embryo. *Nat. Commun.* **8**, 921 (2017).

85. M. Zhu, J. Cornwall-Scoones, P. Wang, C. E. Handford, J. Na, M. Thomson, M. Zernicka-Goetz, Developmental clock and mechanism of de novo polarization of the mouse embryo. *Science* **370**, eabd2703 (2020).

86. K. Taniguchi, Y. Shao, R. F. Townshend, C. L. Cortez, C. E. Harris, S. Meshinchi, S. Kalantry, J. Fu, K. S. O'Shea, D. L. Gumucio, An apicosome initiates self-organizing morphogenesis of human pluripotent stem cells. *J. Cell Biol.* **216**, 3981–3990 (2017).

87. N. Zhang, L. A. Khan, E. Membreno, G. Jafari, S. Yan, H. Zhang, V. Gobel, The *C. elegans* intestine as a model for intercellular lumen morphogenesis and in vivo polarized membrane biogenesis at the single-cell level: Labeling by antibody staining, RNAi loss-of-function analysis and imaging. *J. Vis. Exp.*, 56100 (2017).

88. N. Zhang, E. Membreno, S. Raj, H. Zhang, L. A. Khan, V. Gobel, The *C. elegans* excretory canal as a model for intracellular lumen morphogenesis and in vivo polarized membrane biogenesis in a single cell: Labeling by GFP-fusions, RNAi interaction screen and imaging. *J. Vis. Exp.*, 561001 (2017).

89. S. Brenner, The genetics of *Caenorhabditis elegans*. *Genetics* **77**, 71–94 (1974).

90. T. Stiernagle, Maintenance of *C. elegans* (February 11, 2006). WormBook, ed. The *C. elegans* Research Community, WormBook (2006).

91. L. Timmons, D. L. Court, A. Fire, Ingestion of bacterially expressed dsRNAs can produce specific and potent genetic interference in Caenorhabditis elegans. *Gene* **263**, 103–112 (2001).

92. J. H. Willis, E. Munro, R. Lyczak, B. Bowerman, Conditional dominant mutations in the Caenorhabditis elegans gene act-2 identify cytoplasmic and muscle roles for a redundant actin isoform. *Mol. Biol. Cell* **17**, 1051–1064 (2006).

93. N. Thakur, N. Pujol, L. Tichit, J. J. Ewbank, Clone mapper: An online suite of tools for RNAi experiments in *Caenorhabditis elegans*. *G3 (Bethesda)* **4**, 2137–2145 (2014).

94. D. G. Gibson, L. Young, R.Y. Chuang, J. C. Venter, C. A. Hutchison III, H. O. Smith, Enzymatic assembly of DNA molecules up to several hundred kilobases. *Nat. Methods* **6**, 343–345 (2009).

95. T. Fukushige, M. G. Hawkins, J. D. McGhee, The GATA-factor elt-2 is essential for formation of the Caenorhabditis elegans intestine. *Dev. Biol.* **198**, 286–302 (1998).

96. J. D. McGhee, T. Fukushige, M. W. Krause, S. E. Minnema, B. Goszczynski, J. Gaudet, Y. Kohara, O. Bossinger, Y. Zhao, J. Khattra, M. Hirst, S. J.M. Jones, M. A. Marra, P. Ruzanov, A. Warner, R. Zapf, D. G. Moerman, J. M. Kalb, ELT-2 is the predominant transcription factor controlling differentiation and function of the C. elegans intestine, from embryo to adult. *Dev. Biol.* **327**, 551–565 (2009).

97. M. F. Maduro, J. H. Rothman, Making worm guts: The gene regulatory network of the Caenorhabditis elegans endoderm. *Dev. Biol.* **246**, 68–85 (2002).

98. A. Fire, K. Kondo, R. Waterston, Vectors for low copy transformation of C. elegans. *Nucleic Acids Res.* **18**, 4269–4270 (1990).

99. O. Hobert, PCR fusion-based approach to create reporter gene constructs for expression analysis in transgenic C. elegans. *Biotechniques* **32**, 728–730 (2002).

100. P. Kadandale, I. Chatterjee, A. Singson, Germline transformation of Caenorhabditis elegans by injection. *Methods Mol. Biol.* **518**, 123–133 (2009).

101. C. C. Mello, J. M. Kramer, D. Stinchcomb, V. Ambros, Efficient gene transfer in C.elegans: Extrachromosomal maintenance and integration of transforming sequences. *EMBO J.* **10**, 3959–3970 (1991).

102. D. T. Stinchcomb, J. E. Shaw, S. H. Carr, D. Hirsh, Extrachromosomal DNA transformation of Caenorhabditis elegans. *Mol. Cell. Biol.* **5**, 3484–3496 (1985).

103. L. A. Khan, H. Zhang, N. Abraham, L. Sun, J. T. Fleming, M. Buechner, D. H. Hall, V. Gobel, Intracellular lumen extension requires ERM-1-dependent apical membrane expansion and AQP-8-mediated flux. *Nat. Cell Biol.* **15**, 143–156 (2013).

104. J. J. Ramalho, J. J. Sepers, O. Nicolle, R. Schmidt, J. Cravo, G. Michaux, M. Boxem, C-terminal phosphorylation modulates ERM-1 localization and dynamics to control cortical

actin organization and support lumen formation during *C. elegans* development. *Development* **147**, dev188011 (2020).

105. M. Melak, M. Plessner, R. Grosse, Actin visualization at a glance. *J. Cell Sci.* **130**, 525–530 (2017).

106. M. C. Mariol, L. Walter, S. Bellemin, K. Gieseler, A rapid protocol for integrating extrachromosomal arrays with high transmission rate into the *C. elegans* genome. *J. Vis. Exp.*, e50773 (2013).

107. E. E. Griffin, D. J. Odde, G. Seydoux, Regulation of the MEX-5 gradient by a spatially segregated kinase/phosphatase cycle. *Cell* **146**, 955–968 (2011).

108. G. H. Patterson, J. Lippincott-Schwartz, A photoactivatable GFP for selective photolabeling of proteins and cells. *Science* **297**, 1873–1877 (2002).

109. J. Mijalkovic, B. Prevo, F. Oswald, P. Mangeol, E. J. Peterman, Ensemble and single-molecule dynamics of IFT dynein in *Caenorhabditis elegans* cilia. *Nat. Commun.* **8**, 14591 (2017).

110. C. A. Schneider, W. S. Rasband, K. W. Eliceiri, NIH image to ImageJ: 25 years of image analysis. *Nat. Methods* **9**, 671–675 (2012).

111. Y. M. Sigal, R. Zhou, X. Zhuang, Visualizing and discovering cellular structures with super-resolution microscopy. *Science* **361**, 880–887 (2018).

112. C. Notredame, D. G. Higgins, J. Heringa, T-coffee: A novel method for fast and accurate multiple sequence alignment. *J. Mol. Biol.* **302**, 205–217 (2000).

113. F. Sievers, A. Wilm, D. Dineen, T. J. Gibson, K. Karplus, W. Li, R. Lopez, H. McWilliam, M. Remmert, J. Söding, J. D. Thompson, D. G. Higgins, Fast, scalable generation of high-quality protein multiple sequence alignments using Clustal Omega. *Mol. Syst. Biol.* **7**, 539 (2011).

114. R. S. Kamath, J. Ahringer, Genome-wide RNAi screening in Caenorhabditis elegans. *Methods* **30**, 313–321 (2003).

115. D. Wu, Y. Chai, Z. Zhu, W. Li, G. Ou, W. Li, CED-10-WASP-Arp2/3 signaling axis regulates apoptotic cell corpse engulfment in C. elegans. *Dev. Biol.* **428**, 215–223 (2017).

116. A. Asan, S. A. Raiders, J. R. Priess, Morphogenesis of the *C. elegans* intestine involves axon guidance genes. *PLOS Genet.* **12**, e1005950 (2016).

117. Y. Yan, S. Liu, C. Hu, C. Xie, L. Zhao, S. Wang, W. Zhang, Z. Cheng, J. Gao, X. Fu, Z. Yang, X. Wang, J. Zhang, L. Lin, A. Shi, RTKN-1/Rhotekin shields endosome-associated F-actin from disassembly to ensure endocytic recycling. *J. Cell Biol.* **220**, e202007149 (2021).

118. M. Labouesse, Epithelial junctions and attachments. *WormBook*, 1–21 (2006).

119. H. Liu, S. Wang, W. Hang, J. Gao, W. Zhang, Z. Cheng, C. Yang, J. He, J. Zhou, J. Chen, A. Shi, LET-413/Erbin acts as a RAB-5 effector to promote RAB-10 activation during endocytic recycling. *J. Cell Biol.* **217**, 299–314 (2018).

120. B. Madhu, A. Salazar, T. Gumienny, Caenorhabditis elegans egg-laying and brood-size changes upon exposure to Serratia marcescens and Staphylococcus epidermidis are independent of DBL-1 signaling. *MicroPubl. Biol.* (**2019**).

121. R. S. Kamath, A. G. Fraser, Y. Dong, G. Poulin, R. Durbin, M. Gotta, A. Kanapin, N. le Bot, S. Moreno, M. Sohrmann, D. P. Welchman, P. Zipperlen, J. Ahringer, Systematic functional analysis of the Caenorhabditis elegans genome using RNAi. *Nature* **421**, 231–237 (2003).

122. C. McKeown, V. Praitis, J. Austin, sma-1 encodes a betaH-spectrin homolog required for Caenorhabditis elegans morphogenesis. *Development* **125**, 2087–2098 (1998).

123. J. Cao, J. S. Packer, V. Ramani, D. A. Cusanovich, C. Huynh, R. Daza, X. Qiu, C. Lee, S. N. Furlan, F. J. Steemers, A. Adey, R. H. Waterston, C. Trapnell, J. Shendure,

Comprehensive single-cell transcriptional profiling of a multicellular organism. *Science* **357**, 661–667 (2017).

124. V. Serobyan, Z. Kontarakis, M. A. el-Brolosy, J. M. Welker, O. Tolstenkov, A. M. Saadeldein, N. Retzer, A. Gottschalk, A. M. Wehman, D. Y.R. Stainier, Transcriptional adaptation in *Caenorhabditis elegans*. *eLife* **9**, e50014 (2020).
